# Generic calculation of two-body partial decay widths at the full one-loop level

**DOI:** 10.1140/epjc/s10052-017-5259-x

**Published:** 2017-11-11

**Authors:** Mark D. Goodsell, Stefan Liebler, Florian Staub

**Affiliations:** 1 Sorbonne Universités, UPMC Univ Paris 06, UMR 7589, LPTHE, 75005 Paris, France; 2 CNRS, UMR 7589, LPTHE, 75005 Paris, France; 3 DESY, Notkestraße 85, 22607 Hamburg, Germany; 4 Institute for Theoretical Physics (ITP), Karlsruhe Institute of Technology, Engesserstraße 7, 76128 Karlsruhe, Germany; 5 Institute for Nuclear Physics (IKP), Karlsruhe Institute of Technology, Hermann-von-Helmholtz-Platz 1, 76344 Eggenstein-Leopoldshafen, Germany

## Abstract

We describe a fully generic implementation of two-body partial decay widths at the full one-loop level in the SARAH and SPheno framework compatible with most supported models. It incorporates fermionic decays to a fermion and a scalar or a gauge boson as well as scalar decays into two fermions, two gauge bosons, two scalars or a scalar and a gauge boson. We present the relevant generic expressions for virtual and real corrections. Whereas wave-function corrections are determined from on-shell conditions, the parameters of the underlying model are by default renormalised in a $\overline{\text {DR}}$ (or $\overline{\text {MS}}$) scheme. However, the user can also define model-specific counter-terms. As an example we discuss the renormalisation of the electric charge in the Thomson limit for top-quark decays in the standard model. One-loop-induced decays are also supported. The framework additionally allows the addition of mass and mixing corrections induced at higher orders for the involved external states. We explain our procedure to cancel infrared divergences for such cases, which is achieved through an infrared counter-term taking into account corrected Goldstone boson vertices. We compare our results for sfermion, gluino and Higgs decays in the minimal supersymmetric standard model (MSSM) against the public codes SFOLD, FVSFOLD and HFOLD and explain observed differences. Radiatively induced gluino and neutralino decays are compared against the original implementation in SPheno in the MSSM. We exactly reproduce the results of the code CNNDecays for decays of neutralinos and charginos in *R*-parity violating models. The new version SARAH 4.11.0 by default includes the calculation of two-body decay widths at the full one-loop level. Current limitations for certain model classes are described.

## Introduction

While the large hadron collider (LHC) has completed the standard model (SM) of particle physics with the discovery of a scalar which has all expected properties of the long searched for Higgs boson [1–3], there is no indication for new physics up to now. This has lead to impressive exclusion limits for particles predicted by either supersymmetry (SUSY) or other extensions of the SM which were proposed to resolve the open questions of the SM. However, these exclusion limits for beyond the standard model (BSM) particles depend strongly on the decay properties of these particles. For instance, it is well known that the often cited limits for SUSY squarks and gluinos of 1.8 TeV and more hold only in vanilla models where these states decay to 100 % into a given final state [4–7]. Once realistic decay patterns for the particles are used, the limits become much weaker [8–11]. Thus, a precise knowledge of the branching ratios of BSM states is necessary to be able to draw firm conclusions from the null results. On the other hand, once a new particle is discovered, precise calculations become especially important to extract the underlying parameters and compare against the predictions of many different models.

There has been a lot of effort to improve the predictions of the decay widths for new Higgs-like scalars not only in the minimal supersymmetric standard model (MSSM) [12–39] and the next-to-minimal supersymmetric standard (NMSSM) [40], but also in several singlet and doublet extensions of the SM [41–47]. These results are implemented in public tools such as HDECAY [48, 49], FeynHiggs [50–52] or NMSSMCALC [53]. However, for the plethora of other states, tree-level results are often used. Exceptions are the MSSM, where one-loop corrections to all sfermions and gauginos were discussed in Refs. [54–74]; and neutralino and chargino decays in the NMSSM [75, 76]. For other SUSY models with *R*-parity violation and $\mathcal {CP}$ violation, only a few selected decay modes were discussed so far in Refs. [77, 78]. The available codes to study decays at the one-loop level in the MSSM are SDECAY [79], SUSY_HIT [49] and SFOLD  [80] for sfermion decays, FVSFOLD for flavour violating squark as well as gluino decays, and SloopS [76] and CNNDecays  [75, 77] for neutralino and chargino decays without and with *R*-parity violation.

This limited number of codes and supported models has to be seen in contrast to the increasing number of models which are currently studied. With the increasing limits on the SUSY masses within the MSSM, other ideas for new physics are seeing more and more attention. In order to be able to also give more accurate predictions for the decays in non-minimal SUSY models or also in non-supersymmetric extensions of SM, a high-level of automatisation is needed. A very powerful ansatz to obtain robust results for BSM models has been established with the Mathematica package SARAH  [81–86]: SARAH derives from a short model file all analytical properties of a given model. This information together with generic expressions for various observables is then used to generate Fortran code for SPheno  [87, 88] which can be used to obtain numerical results. Up to now, one- and two-loop masses [89–91], one-loop flavour and precision observables [92], as well as two- and three-body tree-level decays could be obtained via this setup. We have now enhanced the decay calculation to the next level by a generic ansatz to calculate two-body decay widths at the full one-loop level. These extensions are now available with SARAH 4.11.0. In this paper we give all necessary details about the calculation, including the renormalisation scheme; the generic expressions for virtual and real corrections; and the handling of ultraviolet and infrared divergences.

While wave-function corrections are determined from on-shell conditions, the default settings use a $\overline{\text {DR}}$ (or $\overline{\text {MS}}$) renormalisation for the parameters of the underlying model. However, the user can also define model-specific counter-terms in SARAH to be used in the numerical evaluation in SPheno. For now the self-energies of all particles of the underlying model are available for this purpose. Since many particle species receive significant higher-order corrections to their masses and mixing beyond tree level, we also allow the inclusion of mass and mixing corrections for the involved external states. This needs a careful treatment of the infrared divergences, for which we add an infrared counter-term making use of modified Goldstone boson vertices. The setup also supports loop-induced decays. An extension to models with $\mathcal {CP}$ violation or additional charged and massive, coloured vector particles is left for future work. Higgs boson decays, which are very sensitive to corrections of the external states, will be more thoroughly addressed in the future.

The paper is organised as follows: In Sect. 2 we discuss the technical details of the implementation employing external tree-level masses. The incorporation of higher-order corrections for the external states is lined out in Sect. 3. In Sect. 4 we explain how the new features of SARAH and SPheno can be used. In Sect. 5 we present some results obtained with the new machinery: we first show the implementation of counter-terms for two SM examples and then compare our implementation in SARAH with other public codes as SFOLD, HFOLD, CNNDecays. We conclude in Sect. 6. The appendix contains all relevant generic expressions for virtual and real corrections as well as a derivation of the employed Goldstone boson vertices.

## Calculation of decay widths at the full one-loop level

In this section we discuss the technical details of the calculation of two-body decay widths at next-to-leading order for decays that are mediated through a tree-level diagram $X\rightarrow Y_1Y_2$. Our implementation can handle the decays $S\rightarrow SS$, $S\rightarrow SV$, $S\rightarrow VV$, $S\rightarrow FF$, $F\rightarrow FS$ and $F\rightarrow FV$, where *S* denotes a scalar, *F* a fermion and *V* a heavy gauge boson. For loop-induced processes *V* can also be a photon or gluon. At next-to-leading order we include full QCD and electroweak corrections. Thus, apart from ultraviolet divergences, which we need to address through the renormalisation of the parameters of the underlying model, infrared divergences due to massless photons and gluons have to be taken care of. For loop-induced decays the subsequent discussion simplifies substantially, since neither ultraviolet nor infrared divergences have to be tamed, i.e. also the detailed renormalisation of parameters is not of relevance. We continue as follows: we describe the generic form of unpolarised squared matrix elements for two-body decays in the subsequent subsection and thereafter present the various ingredients in terms of tree-level and one-loop amplitudes. This includes vertex and wave-function corrections as well as a discussion of counter-terms. Then in Sect. 2.6 we discuss the relevant real corrections being $1\rightarrow 3$ processes, before we combine the results in Sect. 2.7. Finally, we list the limitations of our implementation in Sect. 2.8.

### Generic unpolarised squared matrix elements

For any two-body decay we write its partial width in the form

2.1 $$\begin{aligned} \Gamma _{X\rightarrow Y_1Y_2}=\frac{1}{16\pi m_X^3}\lambda \left( m_X^2,m_{Y_1}^2,m_{Y_2}^2\right) C_\mathrm{S} C_\mathrm{C} \sum _{h,p} |\mathcal {M}|^2, \end{aligned}$$

where $m_X, m_{Y_1}$ and $m_{Y_2}$ are the masses of the mother and daughter particles in the initial and final state, respectively. We denote their momenta with $p_0,p_1$ and $p_2$, respectively. The sum runs over all helicities (*h*) and polarisations (*p*) in the initial and final state. A symmetry factor $C_\mathrm{S}$ and colour factor $C_\mathrm{C}$ have to be employed. The symmetry factor is $C_\mathrm{S}=1$ by default. For $X=F$ we have $C_\mathrm{S}=\frac{1}{2}$, if $\overline{Y}_1=Y_1$ and $\overline{Y}_2=Y_2$. For $X=S$ it is $C_\mathrm{S}=\frac{1}{2}$, if $\overline{Y}_1=Y_1$ and $\overline{Y}_2=Y_2$ and $Y_1=Y_2$. Therein $\overline{Y}$ denotes the antiparticle of *Y*. The colour factor $C_\mathrm{C}$ for a decaying colour singlet is equal to the dimension of the final states under SU$(3)_C$. For example, for a colour octet decaying into triplets, it yields $C_\mathrm{C}=\frac{1}{2}$, while for more complicated colour configurations $C_\mathrm{C}$ can easily be extracted from the colour-dependent part of the vertex triggering the decay: the colour of the initial state is fixed and a sum over all possible colour combinations in the final state is performed. The Källén function $\lambda$ is given by

2.2 $$\begin{aligned}&\lambda (p_0^2,p_1^2,p_2^2)\nonumber \\&\quad =\sqrt{p_0^4+p_1^4+p_2^4-2p_0^2p_1^2-2p_1^2p_2^2-2p_0^2p_2^2}. \end{aligned}$$

For decay modes with fermions and gauge bosons in the initial and/or final state the matrix elements are a sum over Lorentz structures; we label these with a lower index as $M_i$ and therefore split the total squared amplitude in sums of contributions $M_iM_j^*$, which are multiplied with different kinematic dependences obtained from helicity and polarisation sums. The structures and their sums are given by

2.3 $$\begin{aligned} F \rightarrow FS:&\nonumber \\ \mathcal {M}&\equiv M_1 \bar{v} (p_0) P_L v (p_1) + M_2 \bar{v} (p_0) P_R v (p_1) ,\nonumber \\ \sum _{h,p} |\mathcal {M}|^2&= \frac{1}{2}\left( m_X^2 + m_{Y_1}^2 -\,m_{Y_2}^2\right) \left( M_{1}M^{*}_{1} + M_{2}M^{*}_{2}\right) \nonumber \\&\quad +m_{Y_1}m_{Y_2}(M_{1}M^{*}_{2} + M_{2}M^{*}_{1}) ,\end{aligned}$$

2.4 $$\begin{aligned} S \rightarrow FF:&\nonumber \\ \mathcal {M}&\equiv M_1 \bar{u} (p_1) P_L v (p_2) + M_2 \bar{u} (p_1) P_R v (p_2) ,\nonumber \\ \sum _{h,p} |\mathcal {M}|^2&= \left( m_X^2 -\,m_{Y_1}^2 -\,m_{Y_2}^2\right) \left( M_{1}M^{*}_{1} + M_{2}M^{*}_{2}\right) \nonumber \\&\quad -2m_{Y_1}m_{Y_2}\left( M_{1}M^{*}_{2} + M_{2}M^{*}_{1}\right) ,\end{aligned}$$

2.5 $$\begin{aligned} S \rightarrow SV:&\nonumber \\ \mathcal {M}&\equiv \epsilon _\mu ^*(p_2) (p_0^\mu + p_1^\mu ) M ,\nonumber \\ \sum _{h,p} |\mathcal {M}|^2&= \frac{1}{4m_{Y_2}^2}\left[ m_X^4 + \left( m_{Y_1}^2 -\,m_{Y_2}^2\right) ^2 \right. \nonumber \\&\quad \left. -\,2m_X^2\left( m_{Y_1}^2+m_{Y_2}^2\right) \right] MM^{*} ,\end{aligned}$$

2.6 $$\begin{aligned} S \rightarrow SS:&\nonumber \\ \mathcal {M}&\equiv M ,\nonumber \\ \sum _{h,p} |\mathcal {M}|^2&= MM^*. \end{aligned}$$

For $S\rightarrow VV$ we split the squared amplitude as follows:

2.7 $$\begin{aligned} \nonumber \mathcal {M}\equiv&\, \, \epsilon _\mu ^*(p_1) \epsilon _\nu ^* (p_2) \bigg ( M_1 \eta ^{\mu \nu } + M_2 p_0^\mu p_0^\nu \bigg ),\nonumber \\ \sum _{h,p} |\mathcal {M}|^2 =\,&\frac{1}{2m_{Y_2}^2m_{Y_3}^2}\left[ m_X^4 + m_{Y_1}^4 + 10 m_{Y_1}^2m_{Y_2}^2 + m_{Y_2}^4 \right. \nonumber \\&\left. -\,2 m_X^2\left( m_{Y_2}^2+m_{Y_3}^2\right) \right] M_1M^*_1\nonumber \\&+ \frac{1}{8m_{Y_2}^2m_{Y_3}^2}\left[ m_X^4 + (m_{Y_1}^2-m_{Y_2}^2)^2 \right. \nonumber \\&\left. -\,2 m_X^2(m_{Y_2}^2+m_{Y_3}^2)\right] ^2M_2M^*_2\nonumber \\&+ \, \frac{1}{4m_{Y_2}^2m_{Y_3}^2}\left[ m_X^6 -\,3m_X^4\left( m_{Y_1}^2+ m_{Y_2}^2\right) \right. \nonumber \\&-\,\left( m_{Y_1}^2 -\,m_{Y_2}^2\right) ^2\left( m_{Y_1}^2 + m_{Y_2}^2\right) \nonumber \\&\left. +\,\, m_{Y_1}^2\left( 3m_{Y_1}^4 + 2m_{Y_1}^2m_{Y_2}^2 + 3m_{Y_2}^4\right) \right] \nonumber \\&\times \left( M_{1}M^{*}_{2} + M_{2}M^{*}_{1}\right) . \end{aligned}$$

Lastly the squared amplitude for $F\rightarrow FV$ is given by

2.8 $$\begin{aligned} \mathcal {M}&\equiv \epsilon _\mu ^* (p_2) \bigg ( M_1 \bar{v} (p_0) \gamma ^\mu P_L v (p_1) \nonumber \\&\quad + M_2 \bar{v} (p_0)\gamma ^\mu P_R v (p_1)+ M_3 p_0^\mu \bar{v} (p_0) P_L v (p_1)\nonumber \\&\quad + M_4 p_0^\mu \bar{v} (p_0) P_R v (p_1)\bigg ) ,\nonumber \\ \nonumber \sum _{h,p} |\mathcal {M}|^2&= \frac{1}{2m_{Y_2}^2}\left[ m_X^4 + m_{Y_1}^4 + m_{Y_1}^2m_{Y_2}^2 -\,2m_{Y_2}^4 \nonumber \right. \\&\quad \left. +\, \, m_X^2\left( -2m_{Y_1}^2 + m_{Y_2}^2\right) \right] \left( M_1M^*_1+M_2M^*_2\right) \nonumber \\&\quad \nonumber + \frac{1}{8m_{Y_2}^2}\left[ \left( m_X^2 + m_{Y_1}^2 -\,m_{Y_2}^2\right) \left( m_X^4 + \left( m_{Y_1}^2 -\,m_{Y_2}^2\right) ^2\right. \right. \nonumber \\&\quad \left. \left. -\,2m_X^2\left( m_{Y_1}^2 + m_{Y_2}^2\right) \right) \right] (M_3M^*_3+M_4M^*_4)\nonumber \\&\quad \nonumber -\,3m_Xm_{Y_1}(M_1M^*_2+M_2M^*_1)\nonumber \\&\quad \nonumber -\,\frac{1}{4m_{Y_2}^2}\left[ m_{Y_1}\left( m_X^4 + \left( m_{Y_1}^2 -\,m_{Y_2}^2\right) ^2\right. \right. \nonumber \\&\quad \left. \left. -\,2m_X^2\left( m_{Y_1}^2 +m_{Y_2}^2\right) \right) \right] \nonumber \\&\quad \times \left( M_1M^*_3+M_3M^*_1 + M_2M^*_4+M_4M^*_2\right) \nonumber \\&\quad \nonumber -\,\frac{1}{4m_{Y_2}^2}\left[ m_X\left( m_X^4 + \left( m_{Y_1}^2 -\,m_{Y_2}^2\right) ^2 \right. \right. \nonumber \\&\quad \left. \left. -\,2m_X^2\left( m_{Y_1}^2 + m_{Y_2}^2\right) \right) \right] \nonumber \\&\quad \times \left( M_1M^*_4+M_4M^*_1 + M_2M^*_3+M_3M^*_2\right) \nonumber \\&\quad + \frac{1}{4m_{Y_2}^2}\left[ m_Xm_{Y_1}\left( m_X^4 + \left( m_{Y_1}^2 -\,m_{Y_2}^2\right) ^2 \right. \right. \nonumber \\&\quad \left. \left. -\,2m_X^2\left( m_{Y_1}^2 + m_{Y_2}^2\right) \right) \right] \left( M_3M^*_4+M_4M^*_3\right) . \end{aligned}$$

We implemented special cases for final states with vanishing masses, which are not given here.^1^

### Tree-level amplitudes

For the two-body decays at tree level the contributions to the matrix elements $M^T_i$ can be directly identified with the (left- and right-handed) couplings as follows:

2.9 $$\begin{aligned}&F\rightarrow FV:\quad M^T_1=ic_\mathrm{R},\quad M^T_2=ic_\mathrm{L}, \nonumber \\&\qquad F\rightarrow FS:\quad M^T_1=-ic_\mathrm{R},\quad M^T_2=-ic_\mathrm{L} ,\end{aligned}$$

2.10 $$\begin{aligned}&S\rightarrow FF:\quad M^T_1=-ic_\mathrm{R},\quad M^T_2=-ic_\mathrm{L}, \nonumber \\&\qquad S\rightarrow SS:\quad M^T=ic ,\end{aligned}$$

2.11 $$\begin{aligned}&S\rightarrow SV:\quad M^T=-2ic, \nonumber \\&\qquad S\rightarrow VV:\quad M^T_1=ic. \end{aligned}$$

The conventions for the parametrisation of the vertices are summarised in Appendix A. For $F\rightarrow FV$ $M^T_3$ and $M^T_4$ and for $S\rightarrow VV$ $M^T_2$ vanish at tree level, but contributions are generated at the one-loop level.

### One-loop amplitudes

Before discussing the detailed form of vertex and wave-function corrections, we show their combination with the previously presented results. Once the amplitude due to vertex corrections $M^V$ and due to wave-function corrections $M^W$ are split into $M^V_i$ and $M^W_i$, which encode the various contributions to different combinations of helicities and polarisations, they can be added to the tree-level amplitudes as follows:

2.12 $$\begin{aligned} M_i = M^T_i + 2M^V_i + 2M^W_i. \end{aligned}$$

For an exact next-to-leading order calculation the complex-conjugated part of this amplitude $M_i^*$ is inserted in the complex-conjugated matrix elements of Eqs. (2.3)–(2.8), whereas $M^T_i$ is used for the non-conjugated ones. The total partial width is obtained from the real part of the full expressions in Eqs. (2.3)–(2.8). When squaring the amplitude one needs to be careful if external coloured particles are involved. For these cases, SARAH calculates individual colour factors for tree- and loop-level contributions and sums them up congruently. For loop-induced decays the sum of the amplitudes of the vertex corrections $M^V_i$ and wave-function corrections $M^W_i$ is inserted into all occurrences of matrix elements in Eqs. (2.3)–(2.8).

**Fig. 1:**
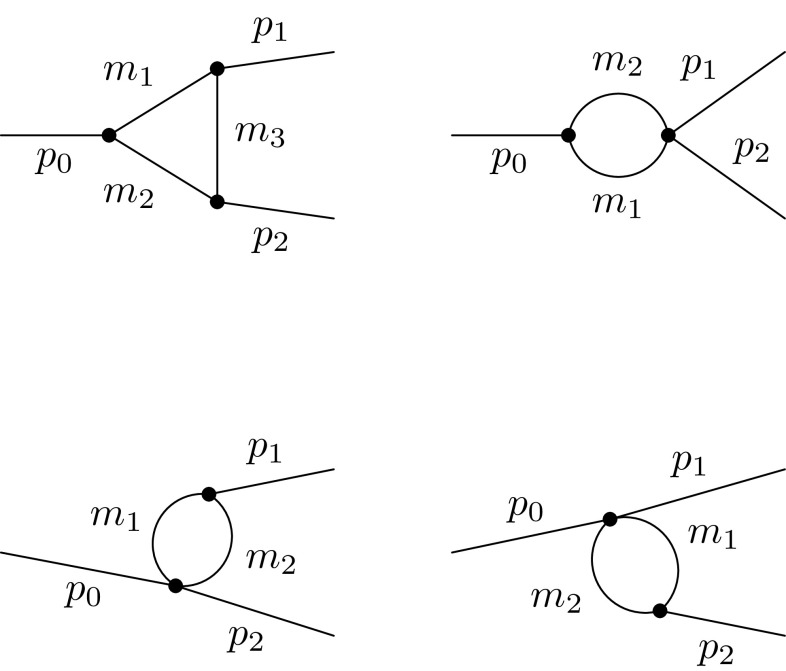
Possible topologies contributing to the vertex corrections

### Vertex corrections

In general, there are four different topologies contributing to the vertex corrections $M^V$, which are shown in Fig. 1. For decays involving fermions only the first topology is of relevance. Depending on the considered decay, different generic diagrams are associated with these topologies. They are depicted for the different decays in Appendix A in Figs. 15, 16, 17, 18, 19 and 20. The results are a function of internal masses $m_1, m_2$ and $m_3$ entering the loop diagrams, but also of the external momenta $p_0, p_1$ and $p_2$. In this section, Sect. 2, their squared values correspond to the squared external masses of the particles, i.e. $m_X^2$, $m_{Y_1}^2$ and $m_{Y_2}^2$, respectively. The calculation of the generic amplitudes $M^{(k)}_i$ for each diagram is straightforward and all results are given in Appendix A. They are obtained with FeynArts  [93] and FormCalc  [94] in Feynman–’t Hooft gauge, i.e. charged and neutral Goldstone bosons are included in the calculation and cancel the unphysical contributions from heavy gauge bosons.

Our results are expressed in terms of Passarino–Veltman integrals obtained through dimensional reduction ($\overline{\text {DR}}$).^2^ Thus, the ultraviolet divergences can be split off in terms of $\Delta =\tfrac{1}{\epsilon }-\gamma _\mathrm{E}+\ln (4\pi )$, where $\epsilon$ regularises the divergence and equals the difference with four dimensions $d=4-2\epsilon$ and $\gamma _\mathrm{E}$ is the Euler–Mascheroni constant. Terms denoted by *r* need to be set to zero in dimensional reduction and correspond to the difference with respect to dimensional regularisation, i.e. it yields $r=1$ for calculations performed in the minimal subtraction scheme ($\overline{\text {MS}}$). By default SARAH sets $r=0$ in SUSY models and $r=1$ in non-SUSY models. In order to match mass dimensions correctly in less than 4 dimensions, dimensional reduction also introduces a new scale *Q*, the renormalisation scale. The generic particle *U* denotes a Faddeev–Popov ghost. In Feynman–’t Hooft gauge their masses are identical masses to the gauge bosons masses, i.e. also ghosts obtain the subsequently discussed regulator mass. Infrared divergences due to massless photons and gluons are regularised through a finite regulator mass. There are no diagrams that contain both photons and gluons. The cancellation of infrared divergences will be addressed in Sect. 2.6, whereas the cancellation of ultraviolet divergences is obtained by adding the subsequently discussed corrections $M^W$.

The combinatoric part to populate the generic diagrams with all possible field insertions in a given model is done by SARAH. SARAH also checks for possible symmetry factors which appear if in the topologies 2–4 in Fig. 1 two real and identical particles are in the loop. In addition, it calculates relevant colour factors to be multiplied with the interference terms $M^T(M^V)^*$.

### Wave-function corrections

The amplitude $M^W$ contains the corrections due to wave-function normalisation as well as the counter-term for the tree-level coupling. They cancel the ultraviolet divergences of the vertex corrections $M^V$ and are mostly determined through renormalisation prescriptions, in contrast to $M^V$. Omitting the complication of fermions and gauge bosons for a moment the amplitude $M^W_{ijk}$ for a vertex of the form $c_{ijk}X_iY_{1j}Y_{2k}$ for the process $X_i\rightarrow Y_{1j}Y_{2k}$ yields

2.13 $$\begin{aligned} M^W_{ijk}&=i\left( \delta c_{ijk} + \frac{1}{2} c_{ljk}\delta Z_{X_l X_i} \right. \nonumber \\&\quad \left. + \frac{1}{2} c_{ilk}\delta Z_{Y_{1l} Y_{1j}} + \frac{1}{2} c_{ijl}\delta Z_{Y_{2l} Y_{2k}}\right) \end{aligned}$$

with the counter-term $\delta c$ of the tree-level coupling *c* and the wave-function corrections $\delta Z$ for the three particles involved. In the last three terms a sum over *l* has to be performed. In the following we will first describe the derivation of the wave-function corrections and then comment on the counter-term for the tree-level coupling.

For the wave-function corrections we employ an on-shell scheme for the three fields *S*, *V* and *F*. For the fermions we distinguish left- and right-handed components $F^L$ and $F^R$. In all cases we allow for mixing among particles induced through loop effects, such that the wave-function corrections are generally matrices. We have

2.14 $$\begin{aligned} V^{\mu ,0}_{i}&\rightarrow Z_{V_i V_j} V_{\mu ,j} =\left( \delta _{ij}+\frac{1}{2} \delta Z_{V_i V_j}\right) V^{\mu }_{j} ,\end{aligned}$$

2.15 $$\begin{aligned} S^0_i&\rightarrow Z_{S_i S_j}S_j = \left( \delta _{ij} + \frac{1}{2}\delta Z_{S_i S_j}\right) S_j ,\end{aligned}$$

2.16 $$\begin{aligned} F^{L0}_i&\rightarrow Z_{F_i F_j}^LF_j = \left( \delta _{ij} + \frac{1}{2}\delta Z^L_{F_i F_j}\right) F^L_j ,\end{aligned}$$

2.17 $$\begin{aligned} F^{R0}_i&\rightarrow Z_{F_i F_j}^RF_j = \left( \delta _{ij} + \frac{1}{2}\delta Z^R_{F_i F_j}\right) F^R_j. \end{aligned}$$

In order to determine the wave-function corrections $\delta Z$ from on-shell conditions, we need the self-energies for our three particle species. Their notation can be read off from the inverse propagators at the one-loop level, which we write as follows:

2.18 $$\begin{aligned} \Gamma _{S_i S_j}(p^2)&= i(p^2 -\,m_S^2)\delta _{ij} + i\hat{\Pi }_{S_i S_j}(p^2) ,\end{aligned}$$

2.19 $$\begin{aligned} \Gamma ^{\mu \nu }_{V_i V_j}(p^2)&= -i g^{\mu \nu }(p^2-m_V^2)\delta _{ij} -\,i\left( g^{\mu \nu } -\,\frac{p^\mu p^\nu }{p^2}\right) \nonumber \\&\quad \times \hat{\Pi }_{V_i V_j}(p^2) -\,i\frac{p^{\mu } p^{\nu }}{p^2} \hat{\Pi }^L_{V_i V_j}(p^2), \end{aligned}$$

**Fig. 2:**
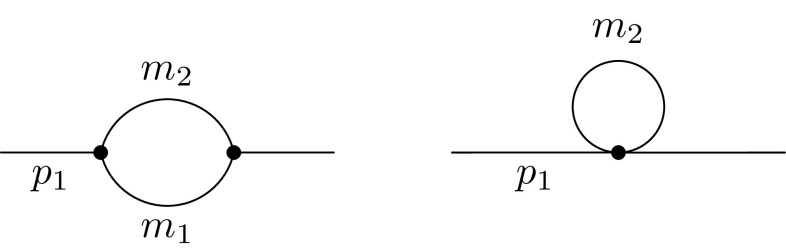
Possible topologies contributing to the wave-function renormalisation

2.20The renormalised self-energies are indicated through $\hat{\Pi }$ and $\hat{\Sigma }$ compared to the unrenormalised ones $\Pi$ and $\Sigma$, which are of relevance for the subsequent discussion. $\Pi _{VV}$ and $\Pi _{SS}$ are the self-energies of the gauge bosons and scalars, respectively. For the gauge bosons we are only interested in the transverse part $\Pi _{VV}$. The only mixing induced between the gauge bosons of the SM is among the photon and the *Z* boson. $P_L$ and $P_R$ are the left- and right-handed projection operators, which split the self-energies of the fermions in $\Sigma ^L$, $\Sigma ^R$, $\Sigma ^{SL}$ and $\Sigma ^{SR}$. The topologies which can contribute are shown in Fig. 2. Moreover, all possible generic diagrams contributing to the fermion, scalar and vector bosons self-energies are shown in Appendix A in Figs. 12, 13 and 14. We give also in Appendix A the expressions for the generic amplitudes for the self-energies and their derivatives. Note that the above structure for the gauge bosons implies the usage of Feynman–’t Hooft gauge. The derivatives of the wave-function corrections, denoted with $\dot{\Pi }$ and $\dot{\Sigma }$, are defined as follows:

2.21 $$\begin{aligned} \dot{\Pi }(k^2)&= \left. \frac{\partial }{\partial p^2}\Pi (p^2)\right| _{p^2=k^2}\quad \text {and}\nonumber \\ \dot{\Sigma }(k^2)&= \left. \frac{\partial }{\partial p^2}\Sigma (p^2)\right| _{p^2=k^2}. \end{aligned}$$

Demanding on-shell conditions for the external states fixes the wave-function corrections. Their derivation can for example be found in Refs. [75, 96] and results in similar expressions for scalars and gauge bosons:

2.22 $$\begin{aligned} \delta Z_{S_i S_i} =\,&-\,\widetilde{\mathrm{Re}} \dot{\Pi }_{S_i S_i}(m_{S_i}^2), \nonumber \\ \delta Z_{V_i V_i} =&-\,\widetilde{\mathrm{Re}} \dot{\Pi }_{V_i V_i}(m_{V_i}^2) ,\end{aligned}$$

2.23 $$\begin{aligned} \delta Z_{S_i S_j} =\,&\frac{2}{m_{S_i}^2 -\,m_{S_j}^2} \widetilde{\mathrm{Re}}\Pi _{S_i S_j}(m_{S_j}^2), \nonumber \\ \delta Z_{V_i V_j} =&\frac{2}{m_{V_i}^2 -\,m_{V_j}^2} \widetilde{\mathrm{Re}}\Pi _{V_i V_j}(m_{V_j}^2). \end{aligned}$$

For the fermions we need to distinguish four cases:

2.24 $$\begin{aligned} \nonumber \delta Z^L_{F_i F_i} =\,&-\widetilde{\mathrm{Re}}\left[ \Sigma ^L_{ii}\left( m_{F_i}^2\right) + m_{F_i}^2 \left( \dot{\Sigma }^L_{ii}\left( m_{F_1}^2\right) + \dot{\Sigma }^R_{ii}(m_{F_i}^2)\right) \right. \nonumber \\&\left. + m_{F_i} \left( \dot{\Sigma }^{SL}_{ii}\left( m_{F_i}^2\right) + \dot{\Sigma }^{SR}_{ii}\left( m_{F_i}^2\right) \right) \right] \nonumber \\ \nonumber \delta Z^R_{F_i F_i} =\,&-\,\widetilde{\mathrm{Re}}\left[ \Sigma ^R_{ii}\left( m_{F_i}^2\right) + m_{F_i}^2 \left( \dot{\Sigma }^L_{ii}\left( m_{F_1}^2\right) + \dot{\Sigma }^R_{ii}\left( m_{F_i}^2\right) \right) \right. \\&\left. + m_{F_i} \left( \dot{\Sigma }^{SL}_{ii}\left( m_{F_i}^2\right) + \dot{\Sigma }^{SR}_{ii}\left( m_{F_i}^2\right) \right) \right] \nonumber \\ \nonumber \delta Z^L_{F_i F_j} =\,&\frac{2m_{F_j}}{m_{F_i}^2 -\,m_{F_j}^2} \widetilde{\mathrm{Re}}\left[ m_{F_j} \Sigma ^L_{ij}\left( m_{F_j}^2\right) + m_{F_i} \Sigma ^R_{ij}\left( m_{F_j}^2\right) \right. \\&\left. +\,\frac{m_{F_i}}{m_{F_j}} \Sigma ^{SL}_{ij}\left( m_{F_j}^2\right) + \Sigma ^{SR}_{ij}\left( m_{F_j}^2\right) \right] \nonumber \\ \delta Z^R_{F_i F_j} =\,&\frac{2m_{F_j}}{m_{F_i}^2 -\,m_{F_j}^2} \widetilde{\mathrm{Re}}\left[ m_{F_i} \Sigma ^L_{ij}\left( m_{F_j}^2\right) + m_{F_j} \Sigma ^R_{ij}\left( m_{F_j}^2\right) \right. \nonumber \\&\left. + \Sigma ^{SL}_{ij}\left( m_{F_j}^2\right) + \frac{m_{F_i}}{m_{F_j}} \Sigma ^{SR}_{ij}\left( m_{F_j}^2\right) \right] . \end{aligned}$$

By $\widetilde{\mathrm{Re}}$ we indicate that $\Pi$ and $\Sigma$ entering $\delta Z$ include only the real parts of the loop functions, whereas couplings enter with real and imaginary components. In case of $\mathcal {CP}$ violation the definition of wave-function corrections usually distinguishes between in- and outgoing particles in order to correctly multiply absorptive parts of self-energies with complex couplings, see the appendix of Ref. [66]. This is beyond our implementation. We note that despite the fact that we employ on-shell conditions to determine the wave-function corrections our external particles are not necessarily on-shell particles, see the discussion at the end of this section.

With this setup at hand we can also define counter-terms to be used for tree-level rotation matrices, which at lowest order transform gauge into mass eigenstates. Those counter-terms enter the counter-term of the tree-level coupling. For any particle species $\Phi$ in mass eigenstates, which is obtained from gauge eigenstates $\Phi '$ through $\Phi _i=R^\Phi _{ij}\Phi '_j$, the counter-term is given by

2.25 $$\begin{aligned} \delta R^\Phi _{ij} = \frac{1}{4} \sum _k \left( \delta Z_{\Phi _i\Phi _k} -\,(\delta Z_{\Phi _k\Phi _i})^*\right) R^\Phi _{kj}. \end{aligned}$$

For fermions left- and right-handed states are rotated with two matrices, such that two counter-terms employing left- and right-handed wave-function corrections also need to be defined. For Majorana fermions we refer to Ref. [75]. It is well known that the definition of such counter-terms for mixing matrices based on the wave-function corrections needs a proper treatment of Goldstone boson tadpole contributions in order to achieve gauge invariance; see Ref. [75] for a more detailed discussion. Since we work in Feynman–’t Hooft gauge we can completely omit these Goldstone boson tadpole contributions, since they ultimately cancel between the wave-function corrections and the counter-term of the mixing matrices. As for the vertex corrections, SARAH inserts all combination of particle species in the generated code, and includes colour as well as symmetry factors.

The non-trivial and mostly non-automatisable part of the calculation of two-body partial decay width is the renormalisation prescription used for the bare parameters of the underlying theory, which enter the tree-level coupling counter-term of the two-body decay under consideration. The counter-terms are usually chosen depending on the model and process. However, a simple $\overline{\text {DR}}$ (or $\overline{\text {MS}}$) prescription for the renormalisation of the parameters of the underlying theory is always easily applicable: from the $\beta$ functions and anomalous dimensions used for the renormalisation group equations implemented in SARAH  [97–104] we can define all counter-terms of the parameters of the underlying theory to be just proportional to the pure ultraviolet divergence only. We will refer to this scheme as $\overline{\text {DR}}$ (or $\overline{\text {MS}}$) scheme in the following. It is well known that this scheme will not perform well in various cases. Therefore, the user of SARAH can define their own counter-terms; see Sect. 4.2 for a more detailed discussion. We also add an example of a proper renormalisation of the electric charge in Sect. 5.

A consequence of the application of the $\overline{\text {DR}}$ (or $\overline{\text {MS}}$) scheme is that our partial decay widths are left with a dependence on the renormalisation scale *Q* introduced through the regularisation of ultraviolet divergences. This is most prominent in the running of the parameters that enter the tree-level coupling obtained from the renormalisation group equations, which is not cancelled at the one-loop level. In the generated code the scale *Q* is by default set to the average stop mass $\sqrt{m_{\tilde{t}_1}m_{\tilde{t}_2}}$ in supersymmetric models and the top-quark mass $m_t$ in non-supersymmetric models. However, the user can control the scale *Q* in the input file, either throughout SPheno or only for the calculation of the decays at one-loop level; see Sect. 4.2. A common choice for the renormalisation scale is also $Q\sim m_X$ close to the mass of the decaying particle *X*. We refrain from making it the default option, since $Q\sim m_X$ slows down the numerical evaluation substantially. In this case loop contributions need to be evaluated multiple times. We recommend to vary the scale to check the stability of the partial decay width calculation, as we demonstrate in Sect. 5 for the decay of the SM Higgs boson into bottom quarks. If the scale is changed throughout SPheno keep in mind that also masses and thus kinematics can change. For a full on-shell calculation the scale dependence also completely vanishes. We demonstrate this for the decay of the top-quark in Sect. 5. In order to achieve a renormalisation-scale-independent result, external states have to have fixed masses and mixing, which for gauge bosons and fermions can be achieved through the settings explained in Sect. 4.2.

Until now we ignored the fact that particles receive higher-order mass corrections.^3^ By construction we have to employ the mass values at lowest order throughout the calculation. We will discuss in Sect. 3 how for the external states mass corrections and mixing beyond tree level can be incorporated into our calculation. If we allow for mass corrections we limit the discussion to the inclusion of $\overline{\text {DR}}$ ($\overline{\text {MS}}$) corrections to the masses, whereas full on-shell prescriptions for BSM particles (as it would be appropriate) are left for future work. With the outlined procedure in the previous subsections, we obtain a gauge-independent and ultraviolet finite result for the partial width $X\rightarrow Y_1Y_2$, which in the most general case, however, is scale dependent. As mentioned, the cancellation of infrared divergences is addressed in the next section.

### Real corrections

In the previous calculation of vertex and wave-function corrections we regularised infrared divergences through the introduction of a finite, but small regulator mass for the photon and/or gluon. The artificial dependence of the cross section calculation on that mass is cancelled by adding the real emission of a photon and/or gluon to the two-body decay, i.e. by adding three-body decays. For a soft photon and/or gluon a divergence is induced, which can again be regularised through a mass and cancels the mass dependence from the vertex and wave-function corrections. With the help of FeynArts [93] and FormCalc [94] we generated generic results for the emission of one additional photon $\gamma$ or gluon *g* for the previously discussed two-body processes $S\rightarrow SS$, $S\rightarrow SV$, $S\rightarrow VV$, $S\rightarrow FF$, $F\rightarrow FS$ and $F\rightarrow FV$. We denote the real corrections for $X\rightarrow Y_1Y_2+\gamma /g$ by

2.26 $$\begin{aligned} \Gamma _{X\rightarrow Y_1Y_2+\gamma /g}&= \frac{1}{(4\pi )^3m_X}\frac{1}{\pi ^2}\int \frac{\mathrm{d}^3p_1}{2p_1^0}\frac{\mathrm{d}^3p_2}{2p_2^0}\frac{\mathrm{d}^3k}{2k^{0}}\delta ^{4}\nonumber \\&\quad \times \left( p_0-p_1-p_2-k\right) C'_\mathrm{S}\sum _{h,p,c}|\mathcal {M}|^2, \end{aligned}$$

where *k* denotes the momentum of the photon or gluon and momenta with upper index 0 equal the zeroth component of the corresponding four vector. External momenta are set to $p_0^2=m_X^2$, $p_1^2=m_{Y_1}^2$, $p_2^2=m_{Y_2}^2$ and $k^2=0$. We then have $C'_\mathrm{S}=C_\mathrm{S}$ for $S\rightarrow VV$ and $S\rightarrow SV$, otherwise $C'_\mathrm{S}=\tfrac{1}{2}C_\mathrm{S}$. The charge and colour structure is encoded in matrices $C_{ij}$ explained in Appendix B, which is why Eq. (2.26) only contains an additional *c* for colour to be summed over. It is clear that the real corrections due to photon emission and gluon emission can be calculated individually and summed up afterwards. By rewriting denominators in terms of eikonal factors, the above integrals can be mapped onto

2.27 $$\begin{aligned}&I^{j_1j_2}_{i_1i_2}(m_X,m_{Y_1},m_{Y_2}) =\frac{1}{\pi ^2}\int \frac{\mathrm{d}^3p_1}{2p_1^0}\frac{\mathrm{d}^3p_2}{2p_2^0}\frac{\mathrm{d}^3k}{2k^{0}}\delta ^4\nonumber \\&\quad \times \left( p_0-p_1-p_2-k\right) \frac{(\pm 2p_{j_1}\cdot k)(\pm 2p_{j_2}\cdot k)}{(\pm 2p_{i_1}\cdot k)(\pm 2p_{i_2}\cdot k)}, \end{aligned}$$

where $p_{i,j}\in \lbrace p_0,p_1,p_2\rbrace$ and the minus signs refer to cases where $p_{i,j}$ equals the momentum $p_0$ of the initial particle *X*. The notation follows Ref. [96], where also results for the relevant integrals are shown. Only integrals with double lower indices are infrared divergent and thus dependent on the regulator mass in addition. We present our results in Appendix B. Through our procedure we calculate the full soft- and hard emission of such photons and gluons and thus for the three-body decay $S\rightarrow SV+\gamma /g$ also include the four-point interaction, which does not diverge as the regulator mass approaches zero. The correct charge and colour factor assignments in the real corrections are done by SARAH as explained in Appendix B. Where possible we compared to the analytic results for real corrections implemented in SFOLD [80] and HFOLD [107] as well as the result presented in Ref. [75]. Apart from finite contributions in $S\rightarrow SV$ we found complete agreement.

We avoid additional collinear divergences by keeping finite masses for all three particles in the initial and final state of our two-body decay calculation, if they interact with photons or gluons. Thus, this problem does not arise for e.g. final-state neutrinos. For fully massless charge- and colour neutral particles in the final state we implemented dedicated routines for $F\rightarrow F'S\gamma$ and $S\rightarrow F'F\gamma$, where one final-state fermion $F'$ can be massless. Keep in mind that if final-state charged or coloured particles are very light, large collinear logarithms can induce a bad numerical behaviour of our routines. This should not cause problems in practical applications unless charged or coloured states with very small masses ($\ll$keV) are present.

Lastly note that since the real correction decay widths are gauge independent, we performed the calculation in unitary gauge for simplicity. This ensures that the results depend only on the gauge couplings and the original tree-level vertex, and we are not obliged to include would-be Goldstone boson vertices as we do for the corresponding loop corrections. The exception is the decay $S\rightarrow SV+\gamma /g$, where gauge invariance fixes the form of the four-point vertex in terms of the three-point one, and we implicitly assume this relation.

### Combination of results

The partial width at next-to-leading order is thus obtained as follows:

2.28 $$\begin{aligned} \Gamma ^{\text {NLO}}_{X\rightarrow Y_1Y_2} = \Gamma _{X\rightarrow Y_1Y_2} + \Gamma _{X\rightarrow Y_1Y_2+\gamma /g}, \end{aligned}$$

where $\Gamma _{X\rightarrow Y_1Y_2}$ is obtained from Eq. (2.1) with the squared amplitudes given in Eqs. (2.3)–(2.8). The individual parts $M_i$ are taken from Eqs. (2.12) and (2.9) for the complex conjugated and non-complex conjugated squared amplitudes, respectively. $\Gamma _{X\rightarrow Y_1Y_2+\gamma /g}$ are the real corrections from Eq. (2.26), which are calculated individually for photons and gluons and summed up. For loop-induced decays the virtual contributions in $M^V$ are by definition ultraviolet finite, still we also include the described wave-function corrections. We note that ultraviolet finiteness can be checked through a variation of $\Delta$ defined in Sect. 2.4 and infrared finiteness through a variation of the regulator mass for the photon and/or gluon, see Sect. 4 for a description how to access them.

### Current limitations

In the approach described so far, we made some assumptions which make the results not applicable to all models which are currently supported by SARAH.

- While complex parameters in all calculation can be handled in principle, the setup is not yet supposed to be used for $\mathcal {CP}$ violation. The reason is that, for decays of real particles into complex final states, only the decay mode $Y_1 Y_2$ is calculated, while $\overline{Y}_1 \overline{Y}_2$ is assumed to have the same partial width. Also note that in the case of $\mathcal {CP}$ violation a common approach is to define extended wave-function corrections as discussed in the appendix of Ref. [66].
- The calculation of the real divergences has neglected the possibility of massive, coloured vector bosons as for instance in Pati–Salam, deconstructed, or trinification models.
- When using loop-corrected external masses as described in the next section, we need to cure all infrared divergences through a proper treatment of Goldstone boson vertices. Currently we assume that the *W* boson is the only massive, charged vector boson, such that models with a $W'$ cannot be used with loop-corrected external masses.
- Gauge boson decays are not implemented yet. This is partially due to the previously two mentioned limitations. On the other hand for decays of the gauge bosons of the SM our framework can easily be extended, which we leave for future work.

**Fig. 3:**
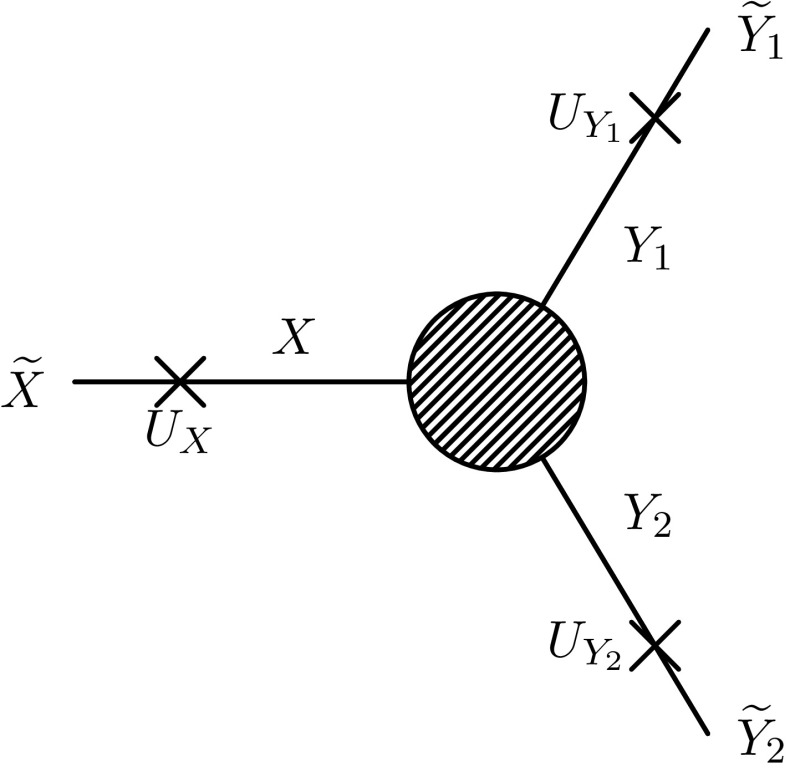
Schematic picture of our method to include higher-order mass and mixing corrections. The calculation presented in Sect. 2 is combined with external normalisation factors and external loop-corrected masses

## Higher-order corrections to the external states

Our previous discussion was based on the usage of tree-level masses for internal as well as external particles. However, the masses of various particle species receive significant higher-order contributions. One way to address this is to adopt an on-shell scheme throughout the calculation, but pure on-shell schemes are not always the best choice for such calculations, as is well known from the Higgs sector of the MSSM. Also even if the calculation is performed in terms of on-shell states in particular in supersymmetric models the limited number of renormalisable parameters in the Lagrangian does not allow for a renormalisation procedure where all on-shell masses correspond to their tree-level values.

Instead, if we do not want to change our renormalisation prescription, we should use the LSZ reduction formula to connect S-matrix elements of on-shell states with Feynman diagrams in our scheme. This results in external normalisation factors and external loop-corrected masses (which need to be distinguished from the previously discussed tree-level masses and mixing matrices). A schematic picture is shown in Fig. 3. For Higgs bosons the approach is discussed in detail in Ref. [108], where such wave-function normalisation factors are denoted *Z*-factors. In particular, as noted there, since we are working at only one loop, there are different truncations of the perturbative series that we can make and different approximations can also be made for expediency. Here we outline the choice(s) that we have made.

Firstly, in the gauge boson sector we recommend to use on-shell values for gauge bosons, see the discussion in Sect. 4. For scalars and fermions, however, we introduce matrices that we denote *U*. Let us introduce our notation for the example of *n* scalars $S_i$, which are mass eigenstates obtained from gauge eigenstates $S_i=R^S_{ij}S'_j$ at tree level and mix at higher orders: following Eq. (2.18) the mass matrices beyond tree level take the form

3.1 $$\begin{aligned} \mathcal {M}_{ij}(p^2)=m_i^2\delta _{ij}-\hat{\Pi }_{ij}(p^2) \end{aligned}$$

with the tree-level masses $m_i$. Let us suppose that for the calculation of the external masses a $\overline{\text {MS}}$ or $\overline{\text {DR}}$ scheme is preferred, i.e. the self-energies $\hat{\Pi }$ and $\hat{\Sigma }$ are renormalised such that only the corresponding ultraviolet divergent part is omitted. In a first approximation we set $p^2=0$ and diagonalise the obtained mass matrix $\mathcal {M}(0)$ through a unitary $(n\times n)$ matrix $U^0$. The tree-level mass eigenstates $S_i$ are thus rotated into states $\tilde{S}_i=U^0_{ij}S_j$ with masses $\widetilde{m}_i$. This matrix $U^0$ incorporates the additional mixing induced at higher orders and in principle corresponds to the *Z*-factors in the $p^2=0$ approximation of Ref. [108].^4^ It is used to rotate the tree-level, vertex and counter-term corrections uniformly by applying it at the amplitude level. For the decay $\tilde{S}_i\rightarrow \tilde{S}_j\tilde{S}_k$ we for example shift the amplitudes by

3.2 $$\begin{aligned} \widetilde{M}_{ijk} = \sum _{s,t,u} U^0_{is}U^0_{jt}U^0_{ku}M_{stu} \end{aligned}$$

for $M=M^T,M^V$ and $M^W$. Also we define rotated tree-level couplings $\widetilde{c}_{ijk}$ in the same manner to be used in the calculation of tree-level ampltiude and real corrections as discussed subsequently. This concept can be very similarly employed for fermions, where again left- and right-handed mixing matrices *U* have to be introduced. Before we discuss the cancellation of ultraviolet and infrared divergences let us note that we also implemented two more methods to obtain the mixing matrix *U*: instead of setting $p^2=0$ an alternative choice is to use $p^2=m_i^2$. This results in the mass eigenvalue $\widetilde{m}_i$, which is used to repeat the procedure iteratively with $p^2=\widetilde{m}_i$ until the mass determination stabilises. Our default choice is that the relative change between the masses of two iterations should be below $10^{-6}$. This procedure needs to be performed for each mass eigenstate $\tilde{S}_i$ separately and the matrix $U^p$ is determined row by row and is thus not unitary any more, as is also well known from the general form of *Z*-factors. Lastly a possible choice is $p^2=m_1^2$, i.e. the external momenta is chosen to be equal to the lightest mass eigenstate. In this case $U^{m_1}$ is again a unitary matrix. We note that the outlined procedure to determine $\widetilde{m}$ and *U* can be performed beyond one-loop level, i.e. for supersymmetric Higgs boson masses corrections at the two-loop level can be incorporated.

### Ultraviolet and infrared divergences

The application of external masses $\widetilde{m}$ different from the tree-level values *m* and mixing matrices *U* in addition to tree-level mixing induces a problem with the cancellation of ultraviolet and infrared divergences. The first problem can be solved easily. We employ tree-level masses *m* for all propagators of loop functions as well as external momenta entering loop functions. This applies to vertex and counter-term corrections and guarantees the cancellation of ultraviolet divergences.

The infrared problem is more demanding. In order to achieve the cancellation of infrared divergences we define infrared counter-terms. These counter-terms encode the mismatch between the masses and mixings of internal and external states and are formally of higher order. These counter-terms are used to shift the wave-function and vertex corrections:

3.3 $$\begin{aligned} M^V&\rightarrow M^V + \delta M^V ,\end{aligned}$$

3.4 $$\begin{aligned} M^{W}&\rightarrow M^{W} + \delta M^{W}. \end{aligned}$$

The aim is to cancel the infrared divergences stemming from $2 M^{T} (M^V + \delta M^V + M^W + \delta M^W)^*$ against the ones from the real emission calculated with loop-corrected masses. The counter-terms $\delta M^V$, $\delta M^W$ are defined to be the difference of the infrared divergences of our default scheme and the one with loop-corrected masses $\widetilde{m}$

3.5 $$\begin{aligned} \delta M^{V,W} = \text {IR}\left( \widetilde{M}^{V,W}\right) - \text {IR}\left( M^{V,W}\right) \end{aligned}$$

where $\text {IR}(M)$ takes only the infrared divergent part of the amplitude *M*. The definition of such counter-terms in supersymmetric models is a common strategy: Ref. [66] introduces an infrared counter-term for the decays $\tilde{t}_1\rightarrow \tilde{b}_iW^+$, see Eq. (191) in Ref. [66], which exactly encodes the difference between on-shell, i.e. loop-corrected, and tree-level masses due to the limited number of renormalisable parameters in the stop and sbottom sectors. Reference [109] discusses the introduction of such counter-terms for the heavy Higgs boson decay $H\rightarrow W^+W^-$. Reference [110] discusses these aspects in detail in the context of a generalised narrow-width approximation, where also infrared divergent parts in the loop contributions are sorted out and evaluated at a common mass scale.

It is clear that the subtraction and re-addition of infrared divergent logarithms as discussed before induces a spurious dependence on other masses, namely on the masses being the counterpart of the regulator mass(es) in the logarithms. This is unavoidable, however, numerically of minor relevance.

In practice, the following procedure is applied:

1. We calculate the virtual corrections using tree-level masses.
2. We extract the infrared divergences of all two- and three-point functions using the results given in Appendix C. These result are used to obtain $\text {IR}\left( M^{V,W}\right)$.
3. We use loop-corrected masses $\widetilde{m}$ throughout all infrared divergent diagrams for the external legs and the particles in the loop. We take again the infrared divergent parts of these amplitudes to obtain $\text {IR}\left( \widetilde{M}^{V,W}\right)$.
4. The calculation of the kinematics as well as of the helicity and polarisations sums for both, the virtual and real corrections, is done with loop-corrected masses.
5. Lastly, the usage of an additional external mixing applied through the mixing matrices *U*, named $U$-factors, works as follows: we rotate the amplitudes of the tree-level, wave-function and virtual corrections according to Eq. (3.2). Instead for the contribution of the infrared counter-term we use rotated tree-level couplings $\widetilde{c}$ rather than rotated amplitudes $\widetilde{M}$. Those rotated couplings also enter the calculation of the real corrections. In this context we note that by construction the infrared counter-term always contains exactly one occurrence of the coupling *c* of the tree-level two-body decay.

These steps give for most cases infrared finite results. However, there is one complication: if the infrared counter-term contains loops with massive gauge bosons, then there will necessarily also be related diagrams with charged Goldstone bosons, and the gauge symmetries require several relationships between the couplings—and masses—of the internal and external particles in order for the infrared divergences to cancel. If we were to apply the above procedure then the infrared counter-term would not be gauge invariant; for these diagrams we therefore use loop-corrected masses and couplings. Note that if we used unitary gauge we could avoid a discussion of corrected couplings in Goldstone boson vertices. Denoting a would-be Goldstone boson by *G*, massive gauge bosons by $V^G$ with masses $m_V^G$ and massless ones by $\gamma ^a$, real scalars as $S_i$ with masses $m_i$ and Dirac fermions as $F_I$ with masses $m_I$, the relevant couplings are

3.6 $$\begin{aligned} \mathcal {L}\supset&\frac{1}{2} c_{ij}^G V^{G\, \mu } (S_j \partial _\mu S_i - S_i \partial _\mu S_j) + \frac{1}{2} c_{ij}^a \gamma ^{a\, \mu } (S_j \partial _\mu S_i - S_i \partial _\mu S_j) \nonumber \\&+ \frac{1}{2} c_{GG'}^{G''} V^{G''\, \mu } (G' \partial _\mu G - G \partial _\mu G') \nonumber \\&+ \frac{1}{2} c_{GG'}^{a} \gamma ^{a\, \mu } (G' \partial _\mu G - G \partial _\mu G') + c_{G}^{aG'} G \gamma _\mu ^a V^{G'\,\mu } \nonumber \\&+ \frac{1}{2} c_{ijG} S_i S_j G +\frac{1}{2} c_{iGG'} S_i G G' \nonumber \\&+ \frac{1}{2} c_{iG}^{G'} V^{G'\, \mu } (G \partial _\mu S_i - S_i \partial _\mu G) + c_i^{GG'} S_i V_{\mu }^G V^{G'\mu } \nonumber \\&+ c^{aGG'} \bigg (\partial ^\mu \gamma _\nu ^a V^{G}_\mu V^{G'\,\nu } + \gamma ^{a\,\mu } \partial _\nu V_\mu ^G V^{G'\,\nu }\nonumber \\&+ \gamma ^{a}_\mu V^{G}_\nu \partial ^\mu V^{G'\,\nu } \bigg ) + \left( c_{IJ}^{a,L} \gamma ^a_\mu + c_{IJ}^{G,L} V^G_\mu \right) \overline{F}_I \gamma ^\mu P_L F_J \nonumber \\&+ \left( c_{IJ}^{a,R} \gamma ^a_\mu + c_{IJ}^{G,R} V^G_\mu \right) \overline{F}_I \gamma ^\mu P_R F_J \nonumber \\&+ c_{IJG}^L G \overline{F}_I P_L F_J + c_{IJG}^R G \overline{F}_I P_R F_J . \end{aligned}$$

The couplings $$c^a_{ij}, c_{GG'}^a, c_{IJ}^{a,L/R} $$ are just generators of the unbroken gauge group in the appropriate representation multiplied by the unbroken gauge coupling. We find that we must enforce the following relations, which we derive in Appendix D:

3.7 $$\begin{aligned} c^{aGG'} =&\, c^a_{GG'} = \frac{1}{m_V^{G'}} c_{G}^{aG'} ,\end{aligned}$$

3.8 $$\begin{aligned} c_{ijG} =&\frac{1}{m_V^G} (m_i^2 -\,m_j^2) c_{ij}^G ,\end{aligned}$$

3.9 $$\begin{aligned} c_{iGG'} =&\frac{m_i^2}{m_V^G m_V^{G'}} c_{i}^{GG'}, \quad c_{iG}^{G'} = \frac{1}{m_V^G} c_i^{GG'} ,\end{aligned}$$

3.10 $$\begin{aligned} c_{IJG}^{L} =&\frac{1}{m_V^G} \left[ m_I c_{IJ}^{G, L} -\,m_J c_{IJ}^{G, R} \right] , \nonumber \\ c_{IJG}^{R} =&-\frac{1}{m_V^G} \left[ m_J^* c_{IJ}^{G, L} -\,m_I^* c_{IJ}^{G, R} \right] . \end{aligned}$$

The implementation in SARAH currently assumes that the gauge sector is that of the SM; so there are no infrared divergent diagrams with neutral Goldstone bosons, and we do not shift their couplings to loop-corrected masses. In practice, a new set of Goldstone vertices is derived by the following relations which is then used in the calculation of the IR shifts:

3.11 $$\begin{aligned} c^L_{F_1 F_2 G^+}&= \frac{m_{F_1} c^L_{F_1 F_2 W} -\,m_{F_2} c^R_{F_1 F_2 W} }{m_W},\nonumber \\ c^R_{F_1 F_2 G^+}&= \frac{m_{F_1} c^R_{F_1 F_2 W} -\,m_{F_2} c^L_{F_1 F_2 W} }{m_W} ,\end{aligned}$$

3.12 $$\begin{aligned} c_{S_1 S_2 G^+}&= \frac{m_{S_1}^2-m_{S_2}^2}{m_W} c_{S_1 S_2 W}, \nonumber \\ c_{S G^+ W}&= \frac{1}{2 m_W} c_{S W W} ,\end{aligned}$$

3.13 $$\begin{aligned} c_{G^+ W \gamma }&= -\,m_W c_{W W \gamma }. \end{aligned}$$

Note that in Eq. (3.11) we explicitly assume no $\mathcal {CP}$ violation.

Employing the outlined procedure we obtain partial decay widths at next-to-leading order with full cancellation of ultraviolet and infrared divergences. Though the application of loop-corrected masses in the infrared counter-term can induce a spurious higher-order gauge dependence, for phenomenological purposes this is, however, small, see e.g. Refs. [75, 77]. Note that for external heavy gauge bosons of the SM we give the option to put the heavy gauge bosons on-shell, such that the cancellation of a gauge dependence in the real corrections among internal gauge bosons and Goldstone bosons is always guaranteed.

### Mixing of species

Particular attention is needed in the calculation of processes where self-energy diagrams allow for the mixing between different particle species beyond tree level. As an example ($\mathcal {CP}$-odd) Higgs bosons including the neutral Goldstone boson can mix with the *Z* boson and even the photon. Then wave-function corrections to the two-body decay come with an internal propagator with a state different from the external state. Such diagrams potentially need to sum up correctly to ensure a gauge-independent partial width. For this purpose, in order to avoid unphysical poles the momenta flowing through the propagators have to match. As an example, Ref. [36] keeps tree-level masses in diagrams mixing Higgs bosons and the *Z* boson/Goldstone boson in the calculation of Higgs decays to Higgs bosons. The Slavnov–Taylor identities then ensure that the sum of the *Z* and Goldstone contributions give zero; see also Refs. [46, 111]. Generally such diagrams are beyond our generic implementation described here and require a process-dependent treatment, i.e. they are not included. Still, in our setup they can easily be added.

### Loop-induced decays

We finish with a remark about loop-induced decays like $F_i \rightarrow F_j \gamma$. Since infrared divergences do not appear for these processes at the one-loop level, there are fewer restrictions on which masses should be used to calculate the vertex one-loop diagrams. As a default setting we therefore use loop-corrected masses everywhere. The reason is that these decays are of particular importance in regions of kinematical thresholds. Thus, the mass splitting between the two massive states should be taken properly into account in the one-loop calculation.

## Implementation in SARAH

### SARAH–SPheno interface

The possibility to calculate one-loop decay widths is available from SARAH 4.11.0. This is a new feature of the SARAH interface to SPheno which was established with SARAH 3.0.0: SARAH generates Fortran code which can be compiled together with the standard SPheno package to obtain a spectrum generator for a given model. The main features of a spectrum generator obtained in that way are a precise mass spectrum calculation including two-loop corrections to real scalars [89–91], a prediction for many precision and flavour observables [92] and up to now the calculation of two- and three-body decays mainly at tree level.

The general procedure to obtain the SPheno code for a given model starts with the download of the most recent SARAH version from HepForge: 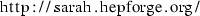 Then the user should copy the tar-file into a directory called $PATH in the following and extract it through: 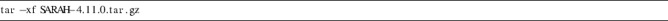 Afterwards, start Mathematica, load SARAH, run a model $MODEL and generate a SPheno version through 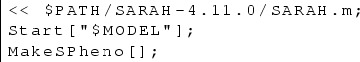 The last command initialises all necessary calculations and writes all Fortran files into the output directory of the considered model. These files can be compiled together with SPheno version 3.3.8 or later. SPheno is also available at HepForge: 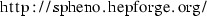 The necessary steps to compile the new files are:


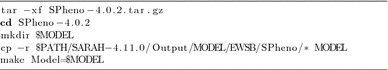


This creates a new binary bin/SPheno$MODEL which reads all input parameters from an external file. SARAH writes a template for this input file which can be used after filling it with numbers by typing:


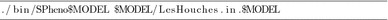


The output is written to


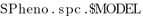


and contains all running parameters at the renormalisation scale, the loop-corrected mass spectrum, as well as all other observables calculated for the given model and parameter point.

The time for generating the Fortran code for the one-loop two-body decays as well as the compilation time of SPheno are extended by these new routines. Therefore, in the case that the user is not interested in the loop-corrected two-body decays, they can be turned off via:


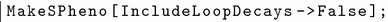


They can be permanently turned off for a given model by adding 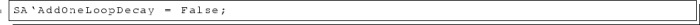 to SPheno.m. Usually, the calculation of the one-loop decays triggers also the calculation of the RGEs even when using the option “OnlyLowEnergySPheno = True;” to generate the SPheno code. The reason is that the $\beta$-functions are used to check the cancellation of ultraviolet divergences. However, for non-supersymmetric models, in particular in the presence of many quartic couplings in the scalar potential, the RGE calculation can be very time-consuming. In this case the option 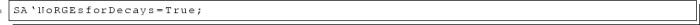 skips the RGE calculation. Of course, the verification of the cancellation of ultraviolet divergences will not be performed with this setting.

### Definition of counter-terms

We included the possibility to define counter-terms to be used in the calculation of the one-loop decays. This is done in SPheno.m via the new array RenConditionsDecays. For instance, the standard renormalisation conditions for the electroweak gauge couplings are set via:


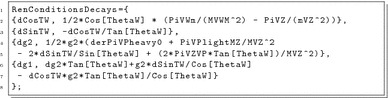


We give an example for the application of the above electroweak counter-terms and their derivation in Sect. 5.1. If RenConditionsDecays is not defined, a pure $\overline{\text {MS}}$/$\overline{\text {DR}}$ renormalisation for the bare parameters of the underlying model is performed. The counter-terms can also be turned on/off in the numerical session via new flags in the SPheno input file as explained in the next subsection. The conventions are:

- The names for the counter-terms are the names of the corresponding parameter starting with d.
- For a rotation angle X, no counter-term for the angle itself is introduced, but for the trigonometric functions involving that angle. Those are called dCosX, dSinX and dTanX.

The following objects can be used to define the counter-terms:

- Parameters of the model: the internal SARAH names must be used.
- Masses of particles in the model: those are called MX where X is the name of the particles in SARAH.
- Self-energies for scalars and vector bosons: those are called PiX where X is the name of the particles in SARAH.
- The derivatives of self-energies of scalars and vector bosons: those are called derPiX where X is the name of the particles in SARAH.
- Self-energies and their derivatives mixing vector bosons: those are called PiXY, respectively, derPiXY, where X and Y are the names of the particles in SARAH and $p^2=m_Y^2$.
- Special self-energies for vector bosons containing only light/heavy states:
  - PiVPlight0/derPiVPlight0: only light degrees of freedom are included in the loops; external momentum $p^2=0$.
  - PiVPlightMZ/derPiVPlightMZ: only light degrees of freedom are included in the loops; external momentum $p^2=m_Z^2$.
  - PiVPheavy0/derPiVPheavy0: only heavy degrees of freedom are included in the loops; external momentum $p^2=0$.
  - PiVPheavyMZ/ derPiVPheavyMZ: only heavy degrees of freedom are included in the loops; external momentum $p^2=m_Z^2$.
- The different parts of the fermion self-energies and their derivatives: those are called SigmaLX, SigmaRX, SigmaSLX, SigmaSR, respectively, DerSigmaLX, DerSigmaRX, DerSigmaSLX, DerSigmaSR, where X is the name of the particles in SARAH.

When SARAH is finished generating the SPheno output, a list of all self-energies and their derivatives which are available in SPheno is stored in SA‘SelfEnergieNames, and the names for all counter-terms are saved in SA‘ListCounterTerms.

One needs to be careful when using self-energies or their derivatives for particles which come with several generations. In this case, the objects defined above are arrays with three indices. The last two indices give the involved generations, the first one the external momentum, e.g.

4.1 $$\begin{aligned} \Pi _{ij}(m^2_{S_k})&\rightarrow \quad \mathtt{PiS(k,i,j)} ,\end{aligned}$$

4.2 $$\begin{aligned} \Sigma ^L_{ij}(m^2_{F_k})&\rightarrow \quad \mathtt{SigmaLF(k,i,j)}. \end{aligned}$$

When defining the counter-terms, commands for matrix or tensor operators should already have been evaluated in Mathematica. Although we offer the possibility to the user to define counter-terms in that way, we want to stress that it has not been tested in practice beyond the examples given in this paper. Thus, this option should be used carefully and the results should be tested throughout, e.g. the ultraviolet finiteness of the partial decay widths is a first test to be performed. Again we emphasise that such counter-terms are for now only applied in the calculation of decay widths. Thus, on-shell prescriptions for the calculation of masses as e.g. known from the neutralino and chargino sector, see Refs. [65, 75, 105, 106], cannot yet be incorporated.

### Options for the evaluation with SPheno

There are several options to steer the performed one-loop calculations which can be controlled via the block DECAYOPTIONS in the Les Houches input file for SPheno. In practice the most important options are:


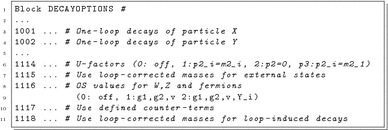


The following settings are possible:

- DECAYOPTIONS[10XY]: the one-loop decays for each particle can individually be turned on (1) or off (0) via these flags. The particle to which a given flag corresponds to is written as comment by SARAH. The default value is 1.
- DECAYOPTIONS[1114]: this defines the choice for the external $U$-factors:
  - 0: no $U$-factors are applied.
  - 1: the $U$-factors including the full $p^2$ dependence are used ($U^p$).
  - 2: the $U$-factors calculated for $p^2=0$ are used ($U^0$).
  - 3: the $U$-factors are calculated from the loop-corrected rotation matrix for the lightest mass eigenstate ($U^{m_1}$).
  The default value is 1.
- DECAYOPTIONS[1115]:
  - 0: the kinematics is done with tree-level masses.
  - 1: the kinematics is done with loop-corrected masses.
  The default value is 1.
- DECAYOPTIONS[1116]:
  - 0: $\overline{\text {MS}}$/$\overline{\text {DR}}$ parameters are used for gauge couplings, *v* and Yukawa couplings.
  - 1: $g_1$, $g_2$ and *v* are set to reproduce the measured values of $M_Z$, $\alpha _{ew}(M_Z)$ and $\sin \Theta _W$.
  - 2: same as 1, but in addition the Yukawa couplings are set to reproduce the measured values of SM fermions.
  The default value is 0.
- DECAYOPTIONS[1117]:
  - 0: the counter-terms defined in RenConditionsDecays are not used.
  - 1: the counter-terms defined in RenConditionsDecays are used.
  The default value is 0.
- DECAYOPTIONS[1118]:
  - 0: for loop-induced decays tree-level masses are used.
  - 1: for loop-induced decays loop-corrected masses are used.
  The default value is 1.

In addition, the following options exist which are mainly supposed to be used for testing and validation of the virtual and real corrections:


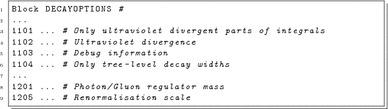


The following settings are possible:

- DECAYOPTIONS[1101]: this option can be used to check the cancellation of ultraviolet divergences.
  - 0: One-loop functions employed in the calculation of one-loop decay widths return the finite part and the ultraviolet divergence defined in DECAYOPTIONS[1102].
  - 1: Only the ultraviolet divergence defined in DECAYOPTIONS[1102] is returned.
  The default value is 0 .
- DECAYOPTIONS[1102]: this option can be used to check the cancellation of ultraviolet divergences. X sets the value used for the ultraviolet divergence $\Delta$ defined in Sect. 2.4. The default value is 0.
- DECAYOPTIONS[1103]:
  - 0: No debug information is shown.
  - 1: Additional information is shown on the screen. This includes the individual contributions from vertex, wave-function and real corrections, which are useful to check the cancellation of ultraviolet and infrared divergences.
  The default value is 0.
- DECAYOPTIONS[1104]: this option can be used to check the consistency between the tree-level and one-loop calculation of decay widths.
  - 0: The one-loop routines return decay widths at NLO.
  - 1: The one-loop routines only return the tree-level decay widths, which can be compared to the tree-level results contained in Block DECAY.
  The default value is 0.
- DECAYOPTIONS[1201]: this option can be used to check the cancellation of infrared divergences. X defines the value (in GeV) used for the photon/gluon mass. The default value is 1.0E-5. Note that this option does not work with loop-corrected masses. The user should ensure that the regulator-mass dependence of vertex and wave-function corrections cancels against the one of the real corrections and yields a regulator-mass-independent decay width. In order to show the individual contributions DECAYOPTIONS[1103] should be set to 1.
- DECAYOPTIONS[1205]: this option can be used to check the renormalisation scale dependence. If defined, X sets the value (in GeV) used for the renormalisation scale *Q* in all one-loop functions employed in the calculation of decay widths. The default option is to use the same renormalisation scale as used in the calculation of masses; see Sect. 2.5.

### Output of SPheno

The results of the one-loop calculation of decay widths are written in the SPheno output. For this purpose, we introduced the keyword DECAY1L beside the standard Block DECAY which lists the results of the ‘old’, i.e. leading order, calculation. Thus, for an arbitrary MSSM point, the output file contains:


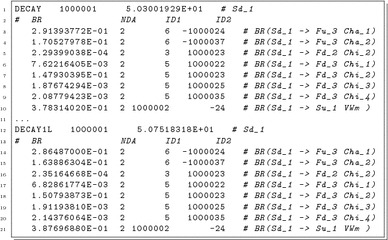


Although this block DECAY1L is not officially supported by the Les Houches conventions, there are the following reasons not to overwrite the results of the ‘old’ calculation:

- The sizes of the one-loop corrections are immediately apparent.
- The results given in DECAY are not only pure tree-level decay widths, but include in particular for the Higgs decays crucial higher-order corrections adapted from literature. Those are beyond the one-loop corrections which we can provide in the new automatised framework at the moment.
- The ‘old’ calculations also include tree-level three-body decays. We leave the choice of how to combine them with the two-body decay widths obtained at the one-loop level to the user.

**Table 1:** Partial decay width $t\rightarrow Wb$ in different schemes, see text for details

Scheme	$\Gamma ^{\text {LO}}_{t\rightarrow Wb}$ (GeV)	$\Gamma ^{\text {NLO,EW}}_{t\rightarrow Wb}$ (GeV)	$\Gamma ^{\text {NLO,EW+QCD}}_{t\rightarrow Wb}$ (GeV)
(1), $\alpha (0)$, $Q=173$ GeV	1.443	1.487 $[+3.0\%]$	1.352 $[-0.135][-9.1\%]$
(1), $\alpha (m_Z)$, $Q=173$ GeV	1.546	1.596 $[+3.2\%]$	1.452 $[-0.144][-9.0\%]$
(2), $\alpha (0)$	1.443	1.519 $[+5.3\%]$	1.384 $[-0.135][-8.9\%]$
(3), $\alpha (m_Z)$	1.546	1.522 $[-1.6\%]$	1.378 $[-0.144][-9.5\%]$

**Table 2:** Partial decay width $H\rightarrow b\bar{b}$ for different values of *Q*, see text for details

Scale	$\Gamma ^{\text {LO}}_{H\rightarrow b\bar{b}}$ (MeV)	$\Gamma ^{\text {NLO,EW}}_{H\rightarrow b\bar{b}}$ (MeV)	$\Gamma ^{\text {NLO,EW+QCD}}_{H\rightarrow b\bar{b}}$ (MeV)
$Q=125$ GeV	1.959	1.972 $[+0.6\%]$	2.376 $[+20.5\%]$
$Q=62.5$ GeV	2.188	2.215 $[+1.5\%]$	2.473 $[+11.6\%]$
$Q=250$ GeV	1.778	1.783 $[+0.3\%]$	2.280 $[+27.8\%]$

## Numerical results

We start this section with two examples for the calculation of two-body decay width in the SM, where we demonstrate the relevance of model- and process-dependent counter-terms. Our default implementation makes use of an $\overline{\text {MS}}$ or $\overline{\text {DR}}$ renormalisation of all parameters of the underlying theory. However, for many processes different schemes are actually better suited. This is particularly true for the calculation of electroweak corrections. For this purpose the user of SARAH can define their own counter-terms, as outlined in Sect. 4.2. We show two simple examples in the SM, namely the calculation of the partial decay width $t\rightarrow Wb$ and $H\rightarrow b\bar{b}$. In the first example we discuss different schemes for the renormalisation of the electric charge, in the second example we show that our $\overline{\text {MS}}$ renormalisation for the bottom-quark Yukawa coupling is actually sufficient. After these examples we continue with a detailed comparison of our implementation with existing codes, among them SFOLD, HFOLD and CNNDecays. Whereas SFOLD and HFOLD are also based on a $\overline{\text {DR}}$ renormalisation of the parameters of the MSSM, the code CNNDecays calculates neutralino and chargino decays in the MSSM, NMSSM and in models with *R*-parity violation again renormalising the electric charge in the Thomson limit. Thereafter, we compare loop-induced decays with the original implementation of SPheno and lastly show the effect of $U$-factors in the calculation of two-body decay widths. A more thorough comparison for Higgs boson decays is left for future work.

### Renormalisation of $\alpha$ and the top-quark width

First we perform a calculation of the top-quark partial width in the decay $t\rightarrow Wb$ including electroweak and QCD corrections using a SM version of SPheno. Since this process is mediated through the gauge coupling $g_2$ of SU$(2)_L$ at tree level, we will discuss the renormalisation of $g_2$ in this context. We choose the following input parameters:

5.1 $$\begin{aligned} m_t&=\!173.3\,\mathrm{GeV},\, m_b\!=\!4.75\,\mathrm{GeV},\, m_W\!=\!80.350\,\mathrm{GeV},\end{aligned}$$

5.2 $$\begin{aligned} \alpha _s(m_Z)&=0.1187,\quad \alpha (m_Z)=1/127.9,\quad V_{tb}=1. \end{aligned}$$

We neglect quark mixing (i.e. the CKM matrix is approximated by the identity matrix). Note that in a more general approach the renormalisation prescription introduced in Eq. (2.25) can be applied to quark mixing. Subsequently we employ external tree-level masses without running, i.e. we effectively calculate with on-shell masses for all three involved particles (setting flag SPHENOINPUT[61] $=$0 to disable the RGE running for the parameters and flag DECAYOPTIONS[1116] $=$2 to use on-shell mass values). This also fixes $g_1, g_2$ and $v, m_W$ from $G_F, m_Z$ and $\alpha (m_Z)$. Our simple $\overline{\text {MS}}$ scheme for the renormalisation of $g_2$ [named scheme (1)] yields

5.3 $$\begin{aligned} (1) \quad \delta g_2 = -\frac{1}{16\pi ^2}\frac{19}{12}g_2^3\Delta . \end{aligned}$$

Next, we provide the decay width for the renormalisation of the electric charge in the Thomson limit of the $ff\gamma$-vertex, i.e. at zero momentum transfer [96]. The counter-terms for the electric charge are given by (see Ref. [112] for an overview)

5.4 $$\begin{aligned}&(2) \quad \alpha (0) \quad \text {and}\quad \delta Z_e(0)=\frac{1}{2} \dot{\Pi }_{\gamma \gamma }(0)-\frac{\tan \theta _W}{m_Z^2}\Pi _{Z\gamma }(0) ,\end{aligned}$$

5.5 $$\begin{aligned}&(3) \quad \alpha (m_Z) \quad \text {and}\quad \delta Z_e(m_Z)=\delta Z_e(0)-\frac{1}{2} \dot{\Pi }_{\gamma \gamma ,\text {light}}(0)\nonumber \\&\qquad \qquad \qquad \qquad \qquad \qquad \qquad \qquad +\frac{1}{2m_Z^2}\widetilde{\mathrm{Re}}\Pi _{\gamma \gamma ,\text {light}}(m_Z^2), \end{aligned}$$

where we distinguish two schemes: At NLO we can make use of the very precise value of $\alpha (0)$ together with the corresponding counter-term $\delta Z_e(0)$ or we employ $\alpha (m_Z)$ and compensate for the shift through the additional terms in $\delta Z_e(m_Z)$. The relevant self-energies include only contributions from light fermions. Ultimately we also need the renormalisation of the weak mixing angle, which is given by

5.6 $$\begin{aligned} \delta \cos \theta _W&=\frac{1}{2} \cos \theta _W\left( \frac{1}{m_W^2}\Pi _{WW}(m_W^2)-\frac{1}{m_Z^2}\Pi _{ZZ}(m_Z^2)\right) \nonumber \\&=-\tan \theta _W\delta \sin \theta _W, \end{aligned}$$

such that in schemes (2) and (3) we obtain

5.7 $$\begin{aligned} \delta g_2 = \left( \delta Z_e-\frac{\delta \sin \theta _W}{\sin \theta _W}\right) g_2. \end{aligned}$$

In this section we keep $\alpha$ and $\alpha _s$ fixed as a function of the renormalisation scale *Q* and therefore schemes (2) and (3) lead to a scale-independent partial decay width, whereas scheme (1) develops a scale dependence. Also, in scheme (1) it is a priori not clear whether $\alpha (0)$ or $\alpha (m_Z)$ is the better choice, so we provide both values. The results are shown in Table 1. We include the LO partial width as well as the NLO partial width only including electroweak and including electroweak and QCD corrections. We also provide (absolute and) relative corrections in brackets. For $\Gamma ^{\text {NLO,EW+QCD}}_{t\rightarrow Wb}$ they are with respect to the partial width $\Gamma ^{\text {NLO,EW}}_{t\rightarrow Wb}$.

For scheme (1) evaluated with $\alpha (m_Z)$ we obtain $\Gamma _{t\rightarrow Wb}^{\text {NLO,EW+QCD}} = 1.434~\mathrm{GeV}$ at $Q=90$ GeV, which demonstrates that the renormalisation scheme dependence is not very pronounced. The absolute QCD correction remains constant (for fixed values of $\alpha$ and $\alpha _s$) and yields $\sim -9$% as expected [113, 114]. It is apparent that schemes (2) and (3) yield very comparable results at NLO despite the different input values for $\alpha$. This is due to the compensation through the shift in the counter-term, which guarantees that the electric charge is renormalised in the Thomson limit. In contrast scheme (1) shows a significant dependence on the input value, where not surprisingly the choice $\alpha (0)$ comes closer to the results in schemes (2) and (3).

We conclude that for precision predictions the proper renormalisation of certain parameters is rather important. The relevant counter-terms for scheme (3) can be defined by the user in the SARAH framework as discussed in Sect. 4.2 through RenConditionsDecays. Note that such counter-terms will only apply at the moment to the calculation of decay widths, not to the calculation of masses.

### Renormalisation of Yukawa couplings and fermionic Higgs decays

We also briefly discuss the calculation of $H\rightarrow b\bar{b}$ in the SM, which is mediated through the bottom-quark Yukawa coupling $Y_b$, and it turns out that the $\overline{\text {MS}}$ renormalisation of $Y_b$ is the preferred choice. A priori, we would expect that the calculation of NLO electroweak corrections [115–117] would again be optimally performed using an on-shell renormalisation of all parameters involved. The counter-term of the bottom-quark Yukawa coupling in the on-shell case is given by

5.8 $$\begin{aligned} \delta Y_b^\mathrm{os}=\frac{1}{v}\left( \sqrt{2}\delta m_b - Y_b\delta v\right) , \end{aligned}$$

such that renormalisation prescriptions for $\delta m_b$ and $\delta v$ are needed. Whereas $\delta m_b$ can be obtained from the self-energies of the down-type quarks, the on-shell renormalisation of the vacuum expectation value depends on other parameters: one requires that renormalised tadpoles vanish as well as the on-shell renormalisation of the Higgs mass and the Higgs self-coupling, see e.g. Ref. [117]. Also, such counter-terms can be implemented in principle through RenConditionsDecays. However, it turns out that electroweak corrections are small ($\sim 1\%$) for a Higgs mass of $m_H=125$ GeV. In contrast QCD corrections are much larger and for them the renormalisation of the Yukawa coupling in the $\overline{\text {MS}}$ scheme is more convenient, since it resums large logarithms [118, 119]. We demonstrate this effect in Table 2, which is obtained with the SM version of SPheno setting flag DECAYOPTIONS[1116] $=$1 and flag SPHENOINPUT[61] $=$1. Through these settings $Y_b$ as well as the gauge couplings, in particular $\alpha _s$, are evaluated at the renormalisation scale *Q* and for $Y_b$ the $\overline{\text {MS}}$ scheme is employed. We again depict the LO as well as the NLO partial width with only electroweak as well as electroweak and QCD corrections including relative corrections. The most relevant parameters are $m_H=125$ GeV, $m_b(m_b)=4.18$ GeV and $\alpha _s(m_Z)=0.1187$. The running to $Q=m_H$ yields $Y_b\propto m_b(m_H)=2.781$ GeV and $\alpha _s(m_Z)=0.1133$.

We see from Table 2 that the electroweak corrections are indeed small. The QCD corrections coincide with the term found in the literature, being $5.667\alpha _s(m_H)/\pi \sim 20.4\%$ [120]. The depicted scale dependence can be used to estimate the remaining uncertainties, which can be significantly reduced by including higher-order QCD corrections beyond one-loop level.

### Comparison with other codes

In order to further validate our calculations and implementations, we compared the obtained results for the MSSM and in *R*-parity violating models against other public tools.

Our comparison is twofold: we compared neutralino and chargino decays into neutralinos and charginos and heavy gauge bosons with CNNDecays, where we employ a full on-shell scheme for the gauge couplings, but work with tree-level neutralino and chargino masses. We use the counter-terms for the electroweak sector as outlined in Sect. 4.2. Given that we adjust all input parameters to be identical, we can therefore exactly reproduce the results of CNNDecays.

Secondly, we made use of the three codes SFOLD, HFOLD and FVSFOLD which also use a $\overline{\text {DR}}$ scheme for the renormalisation of the parameters of the MSSM to calculate one-loop corrections to two-body decays. Since these codes do not make use of external $U$-factors, we have turned them off in our evaluation with SPheno. In addition, we forced all codes to use tree-level ($\overline{\text {DR}}$) masses in all loops and for the kinematics. Thus, the partial widths and the size of the loop corrections presented in the following might be of limited physical interest since the inclusion of $U$-factors and external loop-corrected masses can change the results substantially, see also Sect. 5.4. Thus in this section our aim is to only demonstrate the agreement (and disagreement) between the codes. For the comparison with SFOLD, HFOLD and FVSFOLD we have chosen a parametrisation for the general MSSM which depends only on one dimensionful parameter *m* as follows:

5.9 $$\begin{aligned} M_1&= 0.3 m, \quad M_2 = 0.75 m,\quad M_3 = 2.5 m ,&\nonumber \\ \mu&= 0.5 m, \quad M_A^2 = 3 m^2 ,&\nonumber \\ m_{\tilde{d},11}^2&= m^2,\quad m_{\tilde{d},22}^2 = m^2,\quad m_{\tilde{d},33}^2 = 0.5 m^2 ,&\nonumber \\ m_{\tilde{u},11}^2&= m^2,\quad m_{\tilde{u},22}^2 = m^2,\quad m_{\tilde{u},33}^2 = 0.5 m^2 ,&\nonumber \\ m_{\tilde{q},11}^2&= m_{\tilde{q},22}^2 = m^2,\quad m_{\tilde{q},33}^2 = 2 m^2 ,&\nonumber \\ m_{\tilde{e},11}^2&= m_{\tilde{e},22}^2 = m_{\tilde{e},33}^2 = 0.25 m^2 ,&\nonumber \\ m_{\tilde{l},11}^2&= m_{\tilde{l},22}^2 = 0.25 m^2,\quad m_{\tilde{l},33}^2 = m^2 ,&\nonumber \\ T_{u,33}&= m,\quad T_{e,33} = 0.5 m.&\end{aligned}$$

All other soft-terms are set to zero. This parametrisation has no physical motivation but was chosen in a way to open many different decay channels to be compared among the codes. In addition, we fixed $\tan \beta =10$. We show results for the predicted partial widths when varying *m* from 300 to 2500 GeV.

We also performed a comparison for the loop-induced neutralino and gluino decays which were already implemented in SPheno. The details of this comparison and the outcome are summarised in Sect. 5.3.5.

#### Neutralino and chargino decays in the MSSM and in bilinear *R*-parity violation: CNNDecays vs. SARAH

We compared the decay modes $\tilde{\chi }_i^\pm \rightarrow \tilde{\chi }_j^0 W^\pm$ and $\tilde{\chi }_i^0\rightarrow \tilde{\chi }_j^\mp W^\pm$ as well as $\tilde{\chi }_i^0\rightarrow \tilde{\chi }_j^0 Z$ and $\tilde{\chi }_i^\pm \rightarrow \tilde{\chi }_j^\pm Z$ in the *R*-parity conserving MSSM and adding bilinear *R*-parity violation. Bilinear *R*-parity violation allows an explanation of neutrino masses, but makes the lightest supersymmetric particle (LSP) unstable. Since its decay modes are related to the *R*-parity violating parameters, which are small in order to explain the size of neutrino masses, the decay width of the LSP is also small. We remain with real parameters, both in the MSSM as well as for the *R*-parity breaking parameters. We adjust the tree-level masses and mixing as well as the gauge couplings $g_1$ and $g_2$ to be exactly identical in both codes and also ensure to choose the same renormalisation scale (through DECAYOPTIONS[1205]), namely $Q=m_Z$. Since we employ the renormalisation of the electric charge in the Thomson limit as outlined in Sect. 4.2, the partial decay widths are in principle all renormalisation-scale independent, however, we also want to compare wave-function and vertex corrections individually. We find full agreement between both codes, i.e. numerically identical results beyond 8 digits in the MSSM. In particular this is also true for the vertex and wave-function corrections individually as well as the individual pieces to the counter-terms. Also for the *R*-parity violating decays $\tilde{\chi }_4^0\rightarrow l^\mp W^\pm$ in bilinear *R*-parity violation we find agreement at the per mille level; the smallness of couplings and masses makes those decay modes more sensitive to numerical errors (factors too small or large for the precision of the code). Decay modes into light neutrinos and a gauge boson or a scalar like e.g. $\tilde{\chi }_4^0\rightarrow \nu Z$ or $\nu S$, which are of relevance for *R*-parity violating scenarios, suffer from bad numerical errors. Therefore, neutrino masses, which are analytically zero at tree level, are set to zero in the calculation of one-loop decays. In contrast, the mixing matrix of neutrinos (and neutralinos) remains exact, such that the associated error is small. As we already explained a detailed check of $\mathcal {CP}$-violating scenarios as discussed in Refs. [67, 70, 78] is left for future work for the reasons explained in Sect. 2.8.

#### Sfermion decays in the MSSM: SARAH vs. SFOLD

Next we turn to the comparison of the two-body decays of sfermions in the MSSM. For this purpose, we compared our results against the public code SFOLD 1.2. We have applied several modifications to the code SFOLD:

- The variable to use loop masses either in the loops or in the kinematics are set to 0 in SFOLD.F: 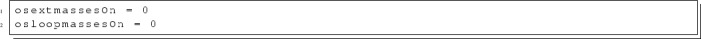 This is done to ensure that the two codes use exactly the same masses everywhere.
- We find a disagreement for the bremsstrahlung routines for $S\rightarrow S V$ decays. Therefore, we add to line 440 in Bremsstrahlung.F of SFOLD the terms: 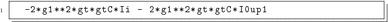

- We find for $S\rightarrow SS$ and $S\rightarrow SV$ decays huge numerical loop-corrections that could even cause a negative width. We could trace back the problem to diagrams with two massive vector bosons in the loop.^5^ The problem is avoided by setting 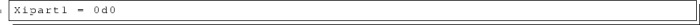 in Decay.F of SFOLD. With this choice, it is no longer possible to change the gauge in SFOLD, but the results are only valid in Feynman-’t Hooft gauge, sufficient for our comparison.
- We find that SFOLD uses a different renormalisation prescription for the rotation matrices: it includes only the divergent parts for the counter-terms, while SARAH calculates the counter-terms from the wave-function renormalisation constants using Eq. (2.25). In particular for $S\rightarrow SV$ decays this can induce large differences in the one-loop corrections. If the finite parts for the counter-terms of the rotation matrices are included, a cancellation between the wave-function corrections and the counter-term correction appears which in sum gives much smaller one-loop corrections. Therefore, we added at the end of the file CalcRenConst99.F of SFOLD the following re-definitions of the counter-terms: 
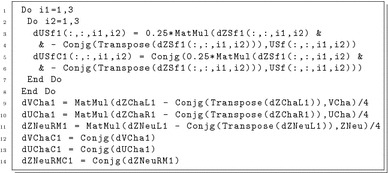

**Fig. 4:**
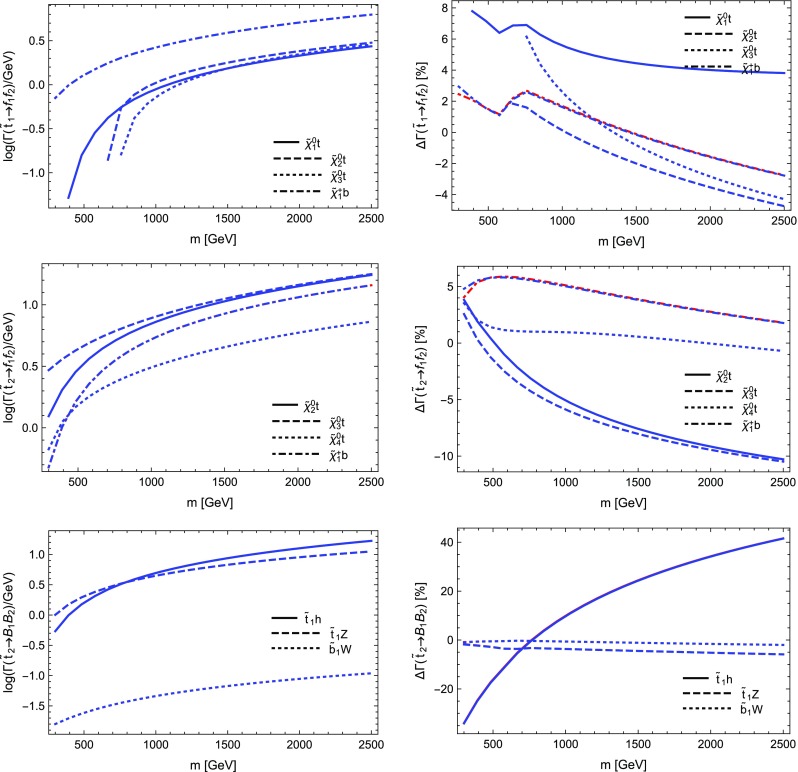
Comparison between SARAH and SFOLD for selected stop decays. On the left, the loop-corrected partial widths are shown. On the right, the relative size of the loop correction is given. Blue lines are obtained with SARAH, red lines with SFOLD

**Fig. 5:**
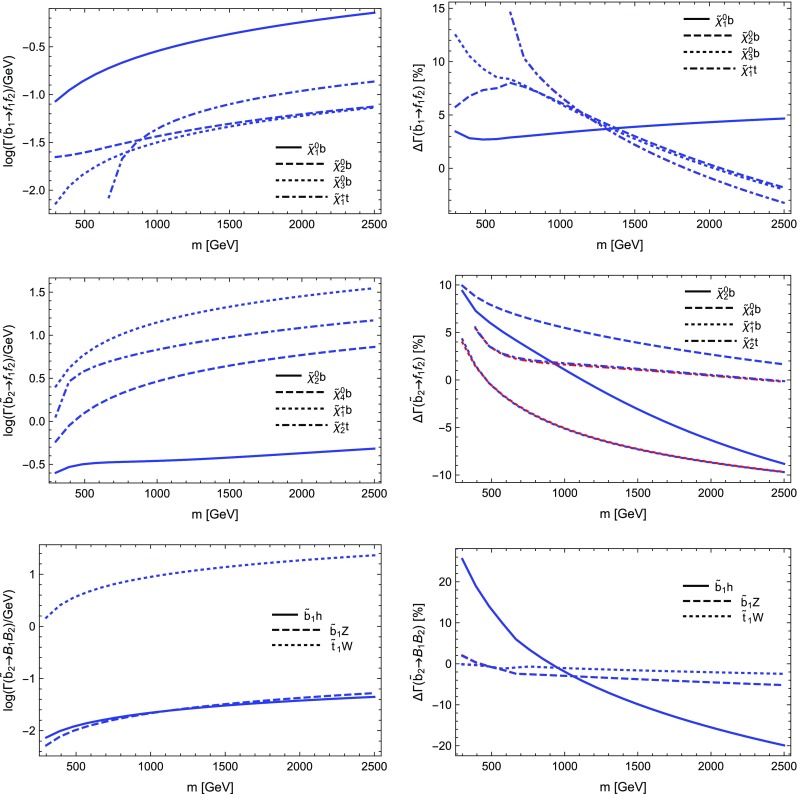
The same as Fig. 4 for sbottom decays

The results for some representative decays for the light and heavy stop are shown in Fig. 4. Here and in the following we give the partial widths at LO and NLO as well as the relative size of the one-loop corrections defined as

5.10 $$\begin{aligned} \Delta \Gamma = \frac{ \Gamma ^{\text {NLO}} - \Gamma ^{\text {LO}} }{ \Gamma ^{\text {LO}}}. \end{aligned}$$

With our described adjustments we find an excellent agreement for the heavy stop decays into a Higgs or a gauge boson and a stop or sbottom. While the corrections for the decays into gauge bosons are comparably small and only of order of a few per-cent, the situation changes if the finite parts for the counter-terms described above are not included. In that case, i.e. when using SFOLD out of the box, the corrections for the decays with a *Z* or *W* boson in the final state can be a factor of 10 larger. For the decays into a pair of fermions we also find very good agreement with only very small differences for small values of *m*. Similarly we show the results for the light and heavy sbottom decays in Fig. 5. Here, the results are very similar to those of the stop decays. We do not add figures for stau or $\tau$-sneutrino decays, or the decays of first and second generation sfermions; they would look very similar to the ones for stop and sbottoms, only the overall size of the loop corrections being smaller. Thus, in total we found a very good agreement between SARAH and SFOLD for all kinds of two-body decays of sfermions.

**Fig. 6:**
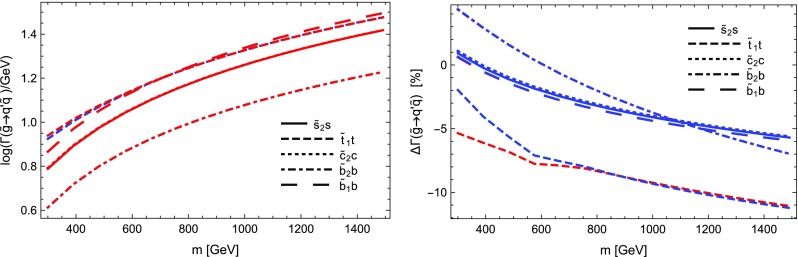
Comparison between SARAH and FVSFOLD for gluino decays. On the left, the loop-corrected partial widths are shown. On the right, the relative size of the one-loop correction is given. Blue lines are obtained with SARAH, red lines with FVSFOLD

#### Gluino decays in the MSSM: SARAH vs. FVSFOLD

In this section we compare the decays of gluinos in the MSSM obtained with SARAH and SPheno against the results generated with the code FVSFOLD. We also performed similar adjustments in FVSFOLD as done for SFOLD for our comparison. However, FVSFOLD already includes the finite parts of the counter-terms of the squark rotation matrices, i.e. it was not necessary to add those. Therefore without any larger adjustments, we find a very good agreement between SARAH and FVSFOLD as shown in Fig. 6 Thus, SARAH reproduces also the result of Ref. [121], namely that the one-loop corrections to gluino decays reduce the decay width by about 10%.

**Fig. 7:**
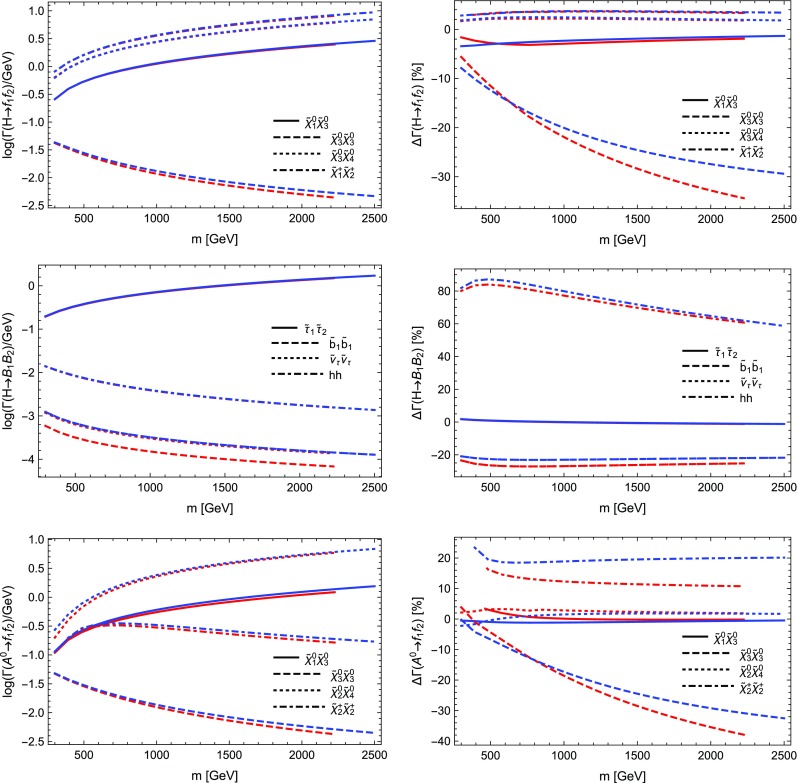
Comparison between SARAH and HFOLD for the heavy $\mathcal {CP}$-even and -odd Higgs. On the left, the loop-corrected partial widths are shown. On the right, the relative size of the loop correction is given. Blue lines are obtained with SARAH, red lines with HFOLD

#### Heavy Higgs decays in the MSSM: SARAH vs. HFOLD

SARAH also makes predictions for the one-loop corrections of Higgs boson decays. However, it must be clearly stated that those predictions have to be interpreted with some care: the automatised calculations are not yet optimised for the calculation of Higgs boson decays, in particular for the SM-like Higgs boson. For such decays, we leave an appropriate definition of counter-terms, following our explanations in Sect. 4.2, to future work. One reason is that for consistency it will be necessary to use the counter-terms in the calculation of the mass spectrum as well. This is, however, not yet possible. We want to stress that SARAH already calculates the light Higgs into SM particle decays by adapting higher-order corrections (even beyond NLO) for the SM and MSSM from literature. Thus, the ‘old’ results obtained with SARAH are expected to be more accurate.

On the other hand, for the decays of heavy Higgs bosons, whose mass corrections are usually much smaller, and/or for decays into BSM states the applied NLO corrections are expected to work well, and the obtained results supersede the pure tree-level calculations often done for these decay modes. In order to validate these results, we compared them against the code HFOLD which also makes predictions for the one-loop corrections of Higgs decays in the MSSM. Here, we made the same adjustments as for FVSFOLD: on-shell masses in loops and kinematics have been turned off. In addition, we needed to turn off all improvements for the ‘old’ calculation in SPheno to obtain equivalent LO results to HFOLD. The results are summarised in Fig. 7 where we compare our results for the decays of the neutral heavy Higgs states *H* and $A^0$ into SUSY particles and SM-like Higgs bosons. Without further modifications we find that the predictions of the size of the one-loop corrections of both codes agree rather well in particular in the dominant decay modes. However, we find that for decay channels with small partial widths also sizeable differences can be present. A detailed investigation of the remaining differences and also a comparison with other Higgs boson decay widths calculations is left for a dedicated work. Such a future investigation should also focus on the detailed derivation and incorporation of the $U$-factors, which admix the Higgs bosons beyond tree level. This is particularly crucial when comparing to codes such as NMSSMCalc or FeynHiggs, where the definition of their *Z*-factors is different [40, 108]. We briefly discuss the relevance of $U$-factors for Higgs boson decays in Sect. 5.4.

**Fig. 8:**
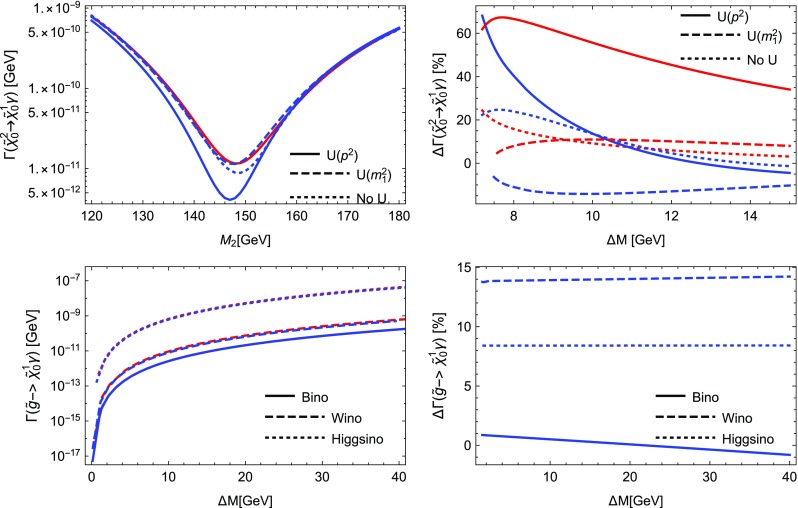
Comparison between SARAH (blue) and SPheno (red) for the loop-induced decays of the neutralino and gluino. On the left, the partial widths are shown. Blue lines are obtained with SARAH, red lines with SPheno. On the right, the relative difference between the codes as a function of the mass splitting is shown (not $\Delta \Gamma$ from Eq. (5.10)). In the case of the neutralino decay we show the impact of the $U$-factors, while for the gluino decays we compare the different kind of neutralinos. The colour code for the upper right figure is: blue for a bino LSP and red for a wino LSP

#### Radiative neutralino and gluino decays: SARAH vs. SPheno

SARAH does not only calculate the one-loop corrections to tree-level two-body decays, but also calculates the LO result for loop-induced decay widths.^6^ The most important applications for these routines are radiatively induced decays of BSM particles. The main candidates for such decays in the MSSM are $\tilde{\chi }^0_2 \rightarrow \tilde{\chi }^0_1 \gamma$ and $\tilde{g} \rightarrow \tilde{\chi }^0_1 g$. Those decays were already implemented in SPheno based on the results of Ref. [123]. For our comparison, we choose parameter points with a light mass splitting between: (i) a bino and wino LSP and NLSP, respectively; (ii) the gluino and all three kinds of neutralinos. For the case of the neutralino decay, the result for the obtained width as a function of the wino mass parameter $M_2$ as well as the relative difference between SPheno and SARAH as a function of the mass splitting are shown in Fig. 8. We show the SARAH results for three different choices of the $U$-factors: (i) without $U$-factors, (ii) using the rotation matrices obtained with the momentum being the mass of the lightest neutralino in all vertices, (iii) using $p^2$-dependent $U$-factors. The second option corresponds to the procedure applied in SPheno and thus we find a reasonable agreement within 10%. The results without $U$-factors are very similar and only very close to the level crossing visible differences occur. However, when using the $p^2$-dependent $U$-factors, the obtained width is significantly smaller. This is due to a cancellation between the vertex and wave-function corrections, which is most efficient when including the $p^2$ dependence in the $U$-factors. For the decays of the gluino into a neutralino and gluon, we find very good agreement between SPheno and SARAH for all three kinds of neutralinos, see again Fig. 8. Note that throughout the calculation of loop-induced decays loop-corrected masses are inserted.

**Fig. 9:**
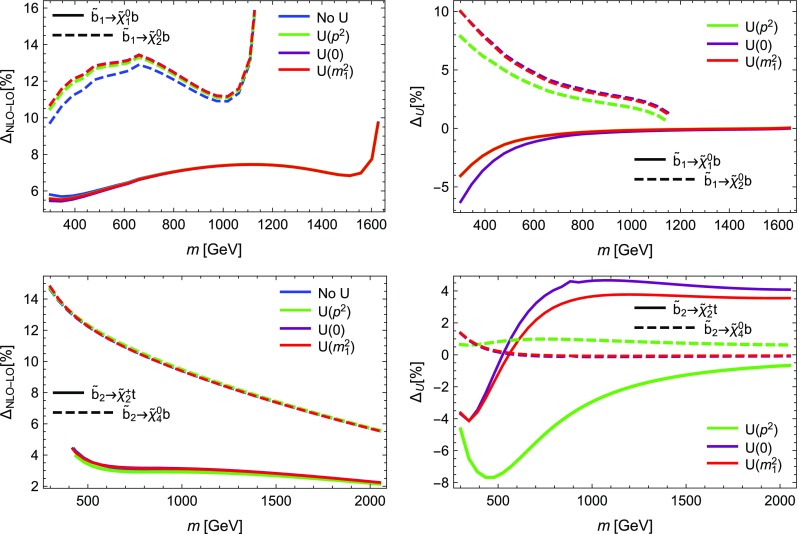
Impact of the external $U$-factors for sbottom decays. On the left side, we show the relative NLO correction when equal $U$-factors are applied at LO and NLO. On the right side, we show the relative NLO correction for different $U$-factors normalised to the relative NLO correction without $U$-factors defined in Eq. (5.11)

**Fig. 10:**
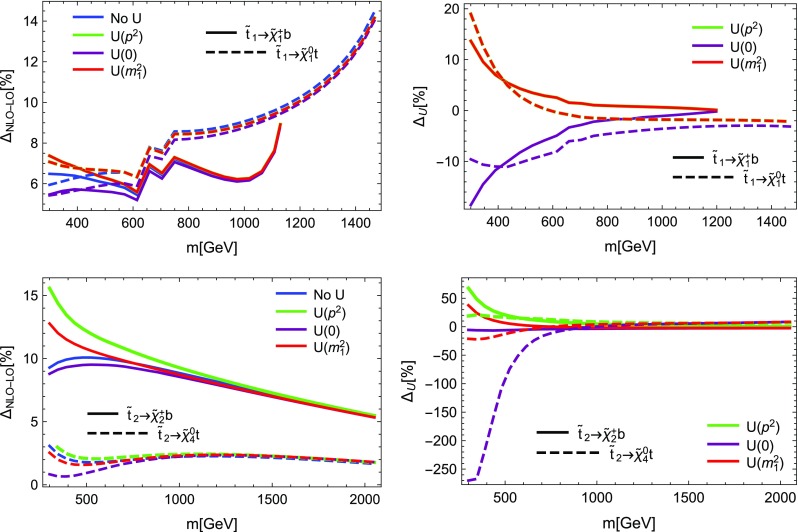
Same as Fig. 9 for decays of stops

**Fig. 11:**
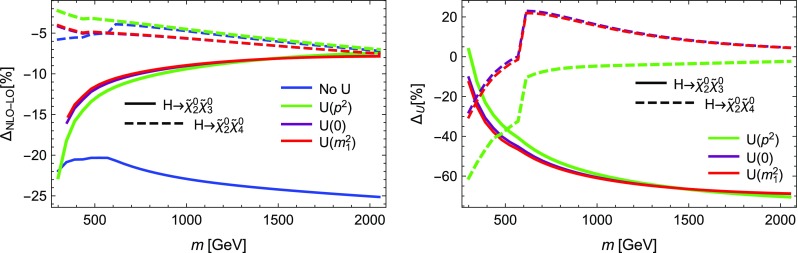
Same as Fig. 9 for decays of the heavy $\mathcal {CP}$-even Higgs boson

### Impact of external $U$-factors

Before we conclude, we want to give some impression of the numerical impact induced by the inclusion of $U$-factors. For this purpose, we show in Figs. 9, 10 and 11 the size of the one-loop corrections for selected stop, sbottom and Higgs decays, respectively, using the three available options to calculate the $U$-factors. We apply loop-corrected $\overline{\text {DR}}$ masses for all cases and particles, i.e. DECAYOPTIONS[1115] $=$1. We focus on two effects: First we apply $U$-factors both at LO and NLO equally and, in the left figures, show the relative correction induced by the NLO corrections. Second, in the right figures, we show the effect of including the $U$-factors in the NLO calculation compared to the NLO decay width calculation without external $U$-factors. More precisely, the value $\Delta _U$ shown is defined as

5.11 $$\begin{aligned} \Delta _U \!= \!\left( \frac{ \Gamma _0^{\text {NLO}}\! - \! \Gamma _0^{\text {LO}} }{ \Gamma _0^{\text {LO}}}\right) ^{-1} \left( \frac{ \Gamma _U^{\text {NLO}} \!-\! \Gamma _U^{\text {LO}} }{ \Gamma _U^{\text {LO}}} - \frac{ \Gamma _0^{\text {NLO}} - \Gamma _0^{\text {LO}} }{ \Gamma _0^{{LO}}}\right) . \end{aligned}$$

Here, $\Gamma _0$ are the decay widths without applying $U$-factors. $\Delta _U$ encodes the difference in the relative correction factor from LO to NLO when applying *U*-factors in contrast to not applying *U*-factors. It thus encodes the effect of *U*-factors in the one-loop correction, factoring out their effect already present at tree level. Depending on the particle species the effect at tree level can already be pronounced, and thus was already included in previous SARAH and SPheno versions. We therefore focus on the effect of the *U*-factors in the relative NLO correction.

For the sbottom decays into gauginos depicted in Fig. 9 the changes due to the inclusion of $U$-factors are moderate. From the left figures it is apparent that the size of NLO corrections is mostly independent of the inclusion of $U$-factors. From the right figures we deduce that the effect of $U$-factors on the relative NLO correction remains below 10% for all choices. The reason is that the left–right mixing in the sbottom sector is in general small and nearly identical at tree and loop level. Thus the *U* matrices are almost diagonal. This is different for the decays of stops shown in Fig. 10 where the left–right mixing is more pronounced. This mixing receives also a sizeable radiative correction which is encoded in the $U$-factors. Consequently, there is also a larger sensitivity on how this matrix is calculated and incorporated as shown in the right two figures. We find that the results without momentum-dependence can differ from the other two options by 30% for the considered decays. For the heavy stop, this effect is even more pronounced. However, for the decay width $\tilde{t}_2\rightarrow \tilde{\chi }_4^0t$, where the relative NLO corrections encoded in $\Delta U$ differ by more than 100%, the absolute NLO correction almost vanishes, as can be seen from the left figure. Thus, in all examples for stop and sbottom decays, the inclusion of $U$-factors gives only a moderate change in the relative NLO corrections once (momentum-dependent) $U$-factors are taken into account compared to the calculation without $U$-factors.

This is slightly different for the heavy Higgs decays shown in Fig. 11. As shown in the left figures all three options for the $U$-factors can alter the size of the relative NLO corrections significantly. In the right figures it is apparent that even for the relative NLO correction differences of 50% and more compared to the calculations without $U$-factors are easily possible, a fact which is well known for Higgs bosons. This shows the need to properly include these factors for Higgs boson decays even if the radiative corrections to the masses are moderate and the particles are clearly separated in their masses. Further studies for Higgs boson decays and a comparison of the $U$-factors to *Z*-factors as discussed in Refs. [40, 108] are in order in future work.

## Conclusions

In this paper we described a fully generic implementation of the calculation of two-body decay widths at the full one-loop level in the SARAH and SPheno framework, which can be used in a wide class of supported models. We presented the necessary generic expressions for virtual and real corrections. Wave-function corrections are determined from on-shell conditions. On the other hand, the parameters of the underlying model are by default renormalised in a $\overline{\text {DR}}$ (or $\overline{\text {MS}}$) scheme. We described how higher-order corrections for the external states can be taken into account. We also explained how we restore gauge invariance as well as ultraviolet and infrared finiteness when setting the external masses to their loop-corrected values. We commented on the drawbacks compared to a full on-shell approach which is model and process dependent.

We have shown how the new features of SARAH and SPheno can be used and how the user can implement own counter-terms to be used for the calculation of two-body decay widths. We studied the impact and relevance of such counter-terms for two examples in the SM, namely the decay to the top-quark and the SM Higgs boson decay into bottom quarks. In addition, we compared our implementation for sfermion and gluino decays within the MSSM against other available codes, namely SFOLD, HFOLD and FVSFOLD, which also employ a $\overline{\text {DR}}$ renormalisation for the MSSM parameters. After a few described adjustments in those codes we found an overall excellent agreement. For the MSSM and *R*-parity violating models we also compared chargino and neutralino decays against CNNDecays, which uses a full on-shell scheme for masses and couplings and found numerically identical results.

The new extension is included in SARAH 4.11.0 and makes it possible to study radiative corrections to two-body decay modes in many different supersymmetric and non-supersymmetric models. However, models with $\mathcal {CP}$ violation and/or (additional) massive gauge bosons charged under U$(1)_\mathrm{em} \times \text {SU}(3)_c$ are not yet supported. This is left for future work. Other future extensions aim at necessary improvements to better handle Higgs boson decays, in particular for the decays of the SM-like Higgs boson to SM particles and the inclusion of external higher-order mass and mixing corrections. Lastly the inclusion of decays of gauge bosons is in order, but it is left for future work.

**Fig. 12:**
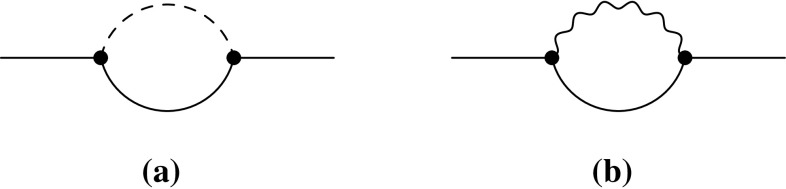
Generic one-loop diagrams for the fermion self-energy

**Fig. 13:**
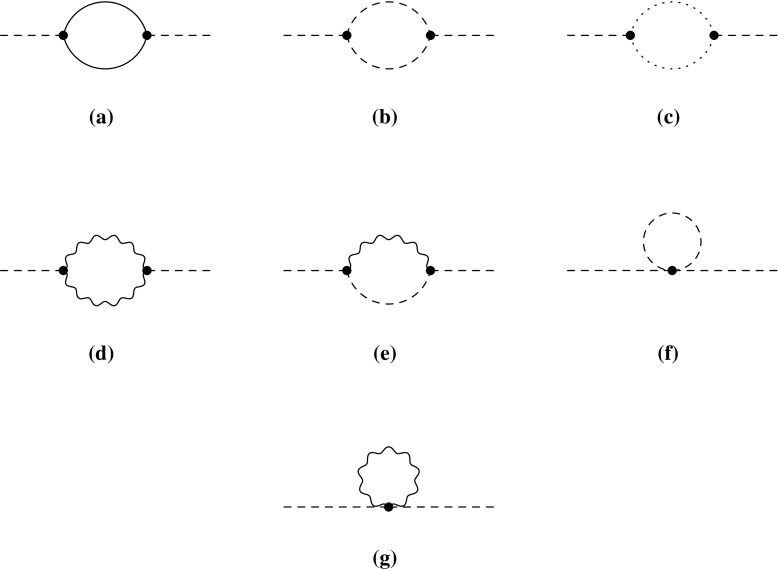
Generic one-loop diagrams for the scalar self-energy

**Fig. 14:**
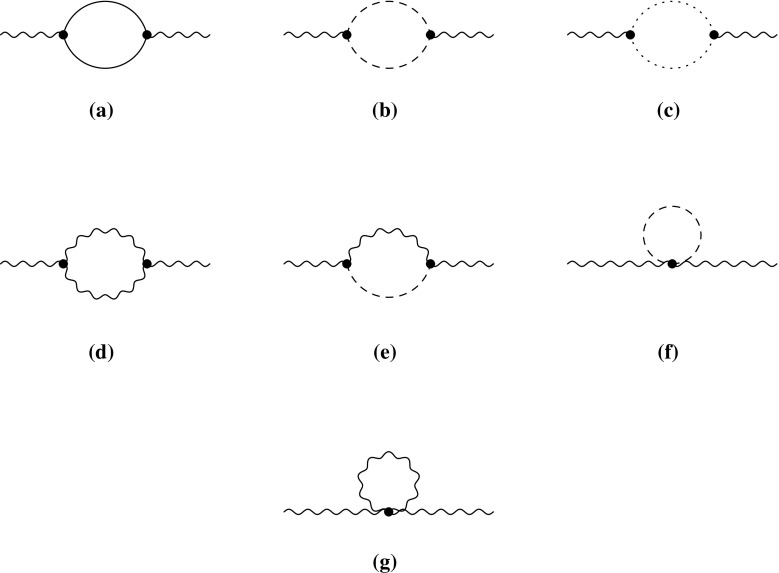
Generic one-loop diagrams for the gauge boson self-energy

## Figures and Tables

**Fig. 15:**
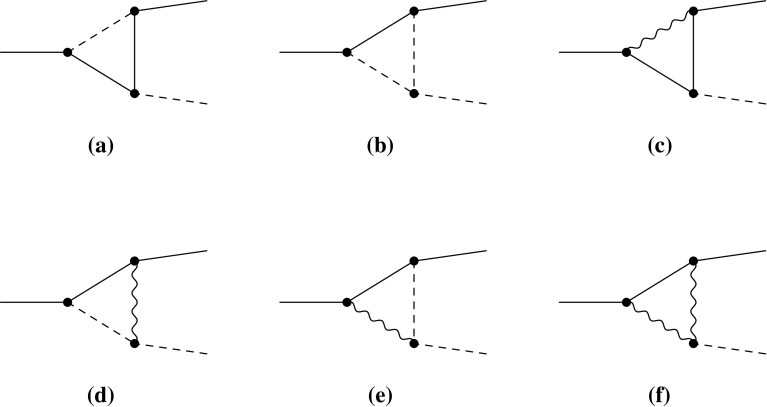
Generic diagrams contributing to $F\rightarrow FS$ decays

**Fig. 16:**
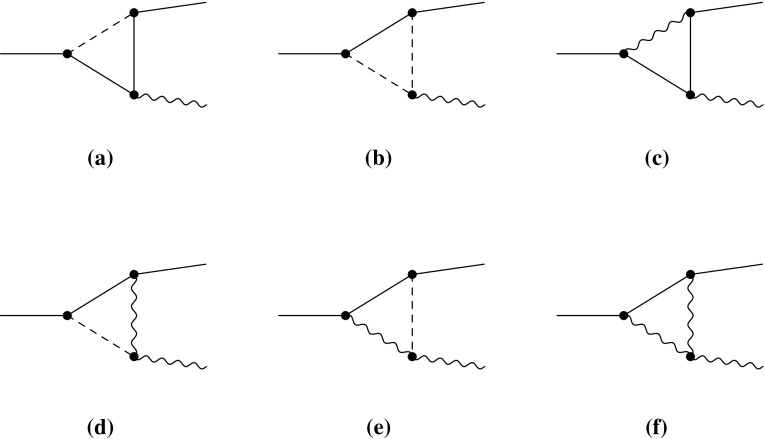
Generic diagrams contributing to $F\rightarrow FV$ decays

**Fig. 17:**
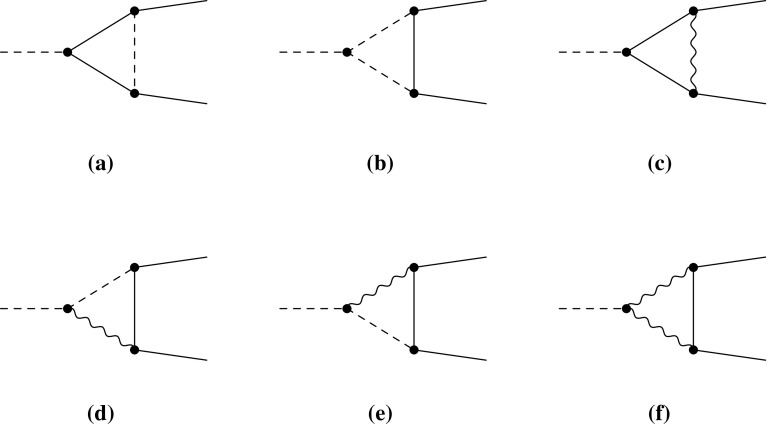
Generic diagrams contributing to $S\rightarrow FF$ decays

**Fig. 18:**
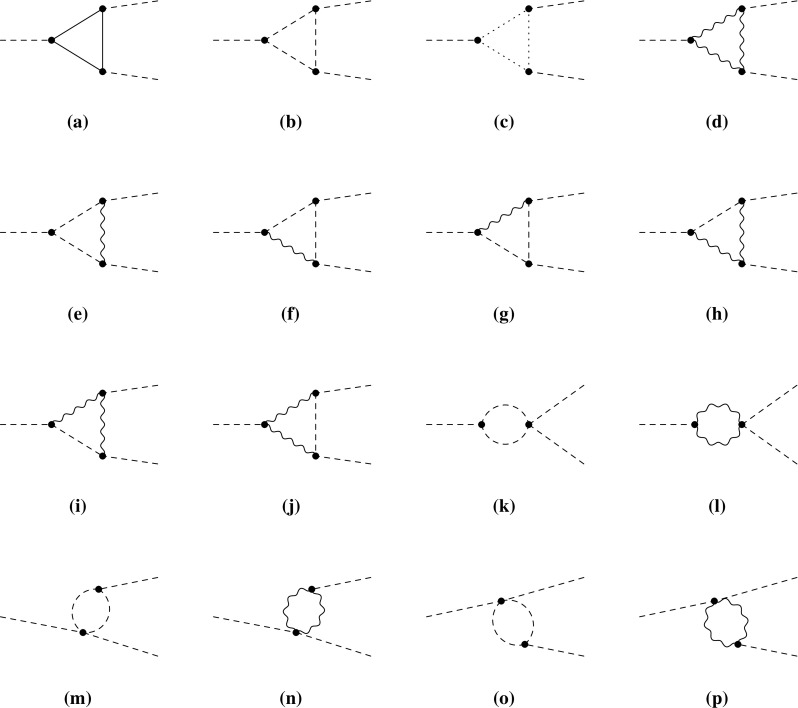
Generic diagrams contributing to $S\rightarrow SS$ decays

**Fig. 19:**
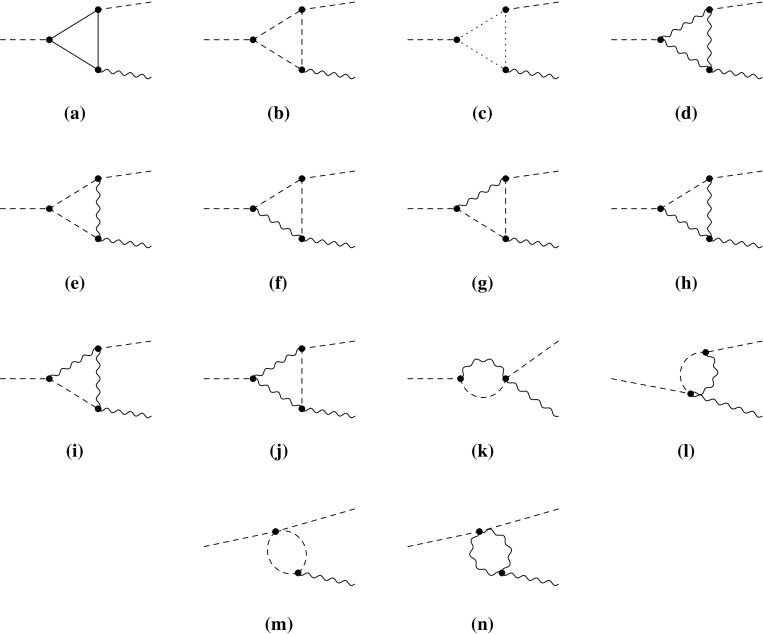
Generic diagrams contributing to $S\rightarrow SV$ decays

**Fig. 20:**
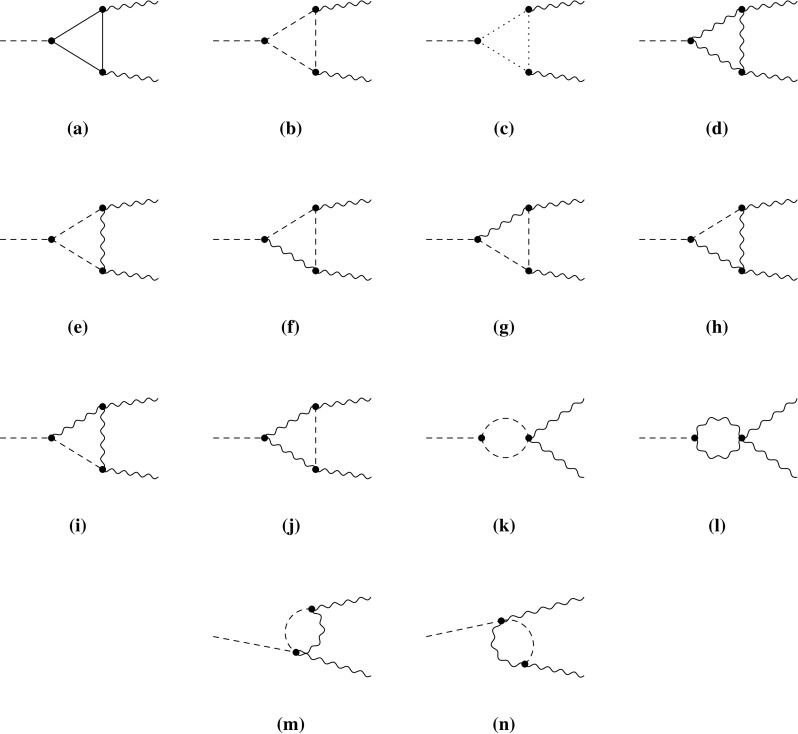
Generic diagrams contributing to $S\rightarrow VV$ decays

**Fig. 21:**
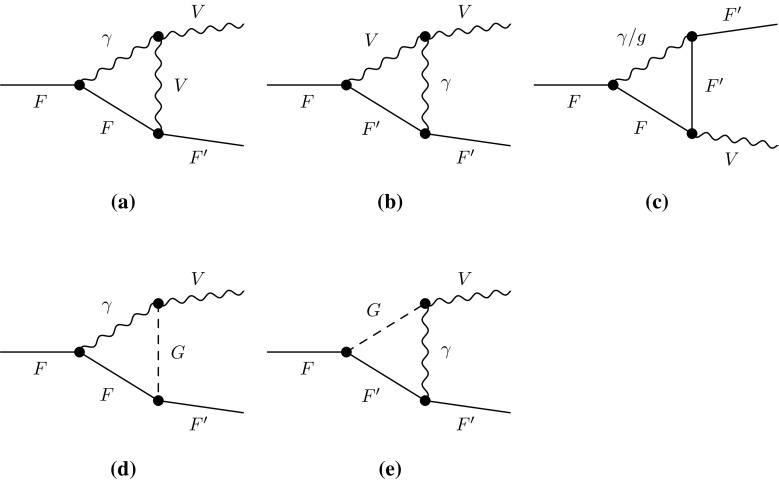
Diagrams in the decay $F \rightarrow F'V$ that are potentially infrared-divergent. Clearly **a** and **d** must combine in a gauge-invariant way, so that when we put the masses of the external states to their loop-corrected values we must also put the masses of the internal legs to those masses and adjust the couplings accordingly. The same is true of the pair **b** and **e**

**Fig. 22:**
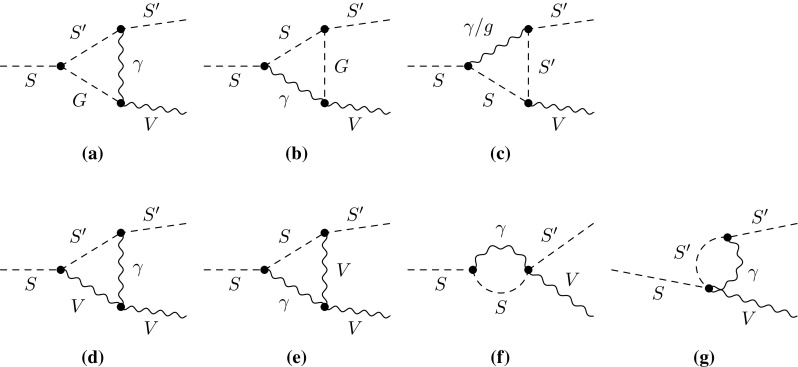
Diagrams in the decay $S \rightarrow S'V$ containing a massless gauge boson propagator. The picture is almost identical to the fermion case, except that we have more “benign” diagrams (**f**) and (**g**)

**Fig. 23:**
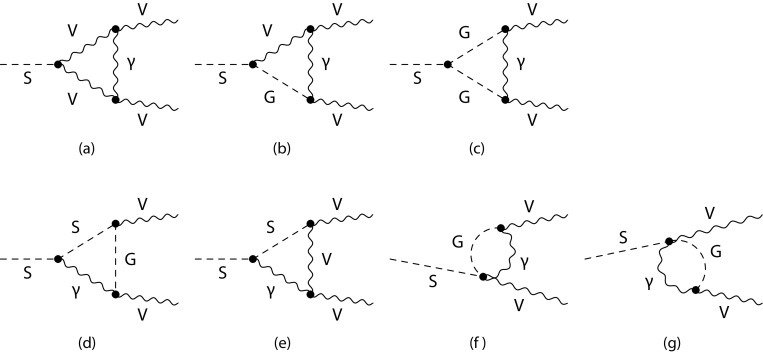
Diagrams in the decay $S \rightarrow VV$ (for two heavy vectors) containing a massless gauge boson propagator. Diagrams (**a**), (**b**) and (**c**) must combine in a gauge-invariant way; as do (**d**) and (**e**); diagrams (**f**) and (**g**) are “benign”

## Appendix A: Conventions and expressions for loop contributions

In this section we present our conventions for vertices and the generic expressions for the loop contributions to the wave-function corrections and the vertex corrections (Figs. 12, 13, 14, 15, 16, 17, 18, 19, 20). We factor out the Lorentz-dependent part of the vertices and work with the following conventions.

- *FFS* vertex ($$\bar{F}_1 F_2 S$$)
  A.1 $$\begin{aligned} c_L P_L + c_R P_R. \end{aligned}$$
- *FFV* vertex ($$\bar{F}_1 F_2 V^\mu $$)
  A.2 $$\begin{aligned} \gamma _\mu (c_L P_L + c_R P_R). \end{aligned}$$
- *SSV* vertex ($$S^*_1 S_2 V^\mu $$)
  A.3 $$\begin{aligned} c (p^{S_1}_{\mu } - p^{S_2}_\mu ). \end{aligned}$$
- *SVV* vertex ($$S_1 V_1^\nu V_2^\mu $$)
  A.4 $$\begin{aligned} c g_{\mu \nu }. \end{aligned}$$
- *VVV* vertex ($$V^1_\mu V^2_\nu V^3_\sigma $$)
  A.5 $$\begin{aligned} c \left[ g_{\mu \nu } (p^{V_2}_\sigma \!- \!p^{V_1}_\sigma ) \!+\! g_{\nu \sigma } (p^{V_3}_\mu \!-\! p^{V_2}_\mu ) \!+\!g_{\mu \sigma }(p^{V_1}_\nu \!-\! p^{V_3}_\nu ) \right] \!. \end{aligned}$$
- *VVVV* vertex ($$V^1_\mu V^2_\nu V^3_\sigma V^4_\rho $$)
  A.6 $$\begin{aligned} c_1 g_{\mu \nu } g_{\sigma \rho } + c_2 g_{\mu \sigma } g_{\nu \rho } + c_3 g_{\mu \rho } g_{\nu \sigma }. \end{aligned}$$

For the loop corrections SARAH inserts the various particle species of the model under consideration also taking into account additional symmetry and colour factors, which are not depicted here. The various contributions are then summed up using

A.7 $$\begin{aligned} M^V_i=\sum _k \frac{1}{16\pi ^2} M^{(k)}_i \end{aligned}$$

and

A.8 $$\begin{aligned} \Pi =&\sum _k \frac{1}{16\pi ^2}\Pi ^{(k)},\quad \dot{\Pi }=\sum _k \frac{1}{16\pi ^2}\dot{\Pi }^{(k)},\end{aligned}$$

A.9 $$\begin{aligned} \Sigma _X=&\sum _k \frac{1}{16\pi ^2}\Sigma _X^{(k)},\quad \dot{\Sigma }_X=\sum _k \frac{1}{16\pi ^2}\dot{\Sigma }_X^{(k)}. \end{aligned}$$

All results in the following are expressed in terms of Passarino–Veltman integrals. The scalar loop functions $A_0$, $B_0$ and $C_0$ are calculated numerically in SPheno according to the standard recipe of Ref. [124]. Tensor integrals are related to the scalar functions according to the famous techniques developed in Ref. [125]. Explicit expressions for derivatives of two-point functions, $\dot{B}_0$, $\dot{B}_1$, which are used by SPheno are given in the appendix of Ref. [75].

### A.1: Wave-function corrections

#### A.1.1: Fermion

(a) *FS* diagram

A.10 $$\begin{aligned} \Sigma ^{(a)}_L =&-2 c^1_L c^2_L B_1(p^2,m_1^2,m_2^2) ,\end{aligned}$$

A.11 $$\begin{aligned} \Sigma ^{(a)}_R =&-2 c^1_R c^2_R B_1(p^2,m_1^2,m_2^2) ,\end{aligned}$$

A.12 $$\begin{aligned} \Sigma ^{(a)}_{SL} =&\,2 c^1_R c^2_L m_1 B_0(p^2,m_1^2,m_2^2) ,\end{aligned}$$

A.13 $$\begin{aligned} \Sigma ^{(a)}_{SR} =&\,2 c^1_L c^2_R m_1 B_0(p^2,m_1^2,m_2^2) ,\end{aligned}$$

A.14 $$\begin{aligned} \dot{\Sigma }^{(a)}_L =&-2 c^1_L c^2_L \dot{B}_1(p^2,m_1^2,m_2^2) ,\end{aligned}$$

A.15 $$\begin{aligned} \dot{\Sigma }^{(a)}_R =&-2 c^1_R c^2_R \dot{B}_1(p^2,m_1^2,m_2^2) ,\end{aligned}$$

A.16 $$\begin{aligned} \dot{\Sigma }^{(a)}_{SL} =&\, 2 c^1_R c^2_L m_1 \dot{B}_0(p^2,m_1^2,m_2^2) ,\end{aligned}$$

A.17 $$\begin{aligned} \dot{\Sigma }^{(a)}_{SR} =&\, 2 c^1_L c^2_R m_1 \dot{B}_0(p^2,m_1^2,m_2^2). \end{aligned}$$

(b) *FV* diagram

A.18 $$\begin{aligned} \Sigma ^{(b)}_L =&-4 c^1_R c^2_R \left( B_1(p^2,m_1^2,m_2^2) + \frac{1}{2} r \right) ,\end{aligned}$$

A.19 $$\begin{aligned} \Sigma ^{(b)}_R =&-4 c^1_L c^2_L \left( B_1(p^2,m_1^2,m_2^2) + \frac{1}{2} r \right) ,\end{aligned}$$

A.20 $$\begin{aligned} \Sigma ^{(b)}_{SL} =&-8 c^1_L c^2_R m_1 \left( B_0(p^2,m_1^2,m_2^2) - \frac{1}{2} r \right) ,\end{aligned}$$

A.21 $$\begin{aligned} \Sigma ^{(b)}_{SR} =&-8 c^1_R c^2_L m_1 \left( B_0(p^2,m_1^2,m_2^2) - \frac{1}{2} r \right) ,\end{aligned}$$

A.22 $$\begin{aligned} \dot{\Sigma }^{(b)}_L =&-4 c^1_R c^2_R \dot{B}_1(p^2,m_1^2,m_2^2) ,\end{aligned}$$

A.23 $$\begin{aligned} \dot{\Sigma }^{(b)}_R =&-4 c^1_L c^2_L \dot{B}_1(p^2,m_1^2,m_2^2) ,\end{aligned}$$

A.24 $$\begin{aligned} \dot{\Sigma }^{(b)}_{SL} =&-8 c^1_L c^2_R m_1 \dot{B}_0(p^2,m_1^2,m_2^2) ,\end{aligned}$$

A.25 $$\begin{aligned} \dot{\Sigma }^{(b)}_{SR} =&-8 c^1_R c^2_L m_1 \dot{B}_0(p^2,m_1^2,m_2^2). \end{aligned}$$

For Majorana fermions, an additional overall factor $\frac{1}{2}$ is present.

#### A.1.2: Scalar

(a) *FF* diagram

A.26 $$\begin{aligned} \Pi ^{(a)}_{SS} =\,&(c^1_L c^2_L + c^1_R c^2_R) G_0(p^2,m_1^2,m_2^2)\nonumber \\&-2 (c^1_L c^2_R + c^1_R c^2_L) B_0(p^2,m_1^2,m_2^2) ,\end{aligned}$$

A.27 $$\begin{aligned} \dot{\Pi }^{(a)}_{SS} =\,&(c^1_L c^2_L + c^1_R c^2_R) \dot{G}_0(p^2,m_1^2,m_2^2)\nonumber \\&-2 (c^1_L c^2_R + c^1_R c^2_L) \dot{B}_0(p^2,m_1^2,m_2^2), \end{aligned}$$

with

A.28 $$\begin{aligned} G_0(p^2,m_1^2,m_2^2) =\,&- A_0(m_1^2) - A_0(m_2^2) \nonumber \\&+ (p^2 - m_1^2 - m_2^2) B_0(p^2,m_1^2,m_2^2) ,\end{aligned}$$

A.29 $$\begin{aligned} \dot{G}_0(p^2,m_1^2,m_2^2) =\,&(p^2 - m_1^2 - m_2^2) \dot{B}_0(p^2,m_1^2,m_2^2)\nonumber \\&+ B_0(p^2,m_1^2,m_2^2). \end{aligned}$$

(b) *SS* diagram

A.30 $$\begin{aligned} \Pi ^{(b)}_{SS} =\,&c^1 c^2 B_0(p^2,m_1^2,m_2^2) ,\end{aligned}$$

A.31 $$\begin{aligned} \dot{\Pi }^{(b)}_{SS} =\,&c^1 c^2 \dot{B}_0(p^2,m_1^2,m_2^2). \end{aligned}$$

(c) *UU* diagram

A.32 $$\begin{aligned} \Pi ^{(c)}_{SS} =\,&-c^1 c^2 B_0(p^2,m_1^2,m_2^2) ,\end{aligned}$$

A.33 $$\begin{aligned} \dot{\Pi }^{(c)}_{SS} =\,&-c^1 c^2 \dot{B}_0(p^2,m_1^2,m_2^2). \end{aligned}$$

(d) *VV* diagram

A.34 $$\begin{aligned} \Pi ^{(d)}_{SS} =\,&4 c^1 c^2 \left( B_0(p^2,m_1^2,m_2^2) - \frac{1}{2} r \right) ,\end{aligned}$$

A.35 $$\begin{aligned} \dot{\Pi }^{(d)}_{SS} =\,&4 c^1 c^2 \dot{B}_0(p^2,m_1^2,m_2^2). \end{aligned}$$

(e) *SV* diagram

A.36 $$\begin{aligned} \Pi ^{(b)}_{SS} =\,&c^1 c^2 F_0(p^2,m_1^2,m_2^2) ,\end{aligned}$$

A.37 $$\begin{aligned} \dot{\Pi }^{(b)}_{SS} =\,&c^1 c^2 \dot{F}_0(p^2,m_1^2,m_2^2) \end{aligned}$$

with

A.38 $$\begin{aligned} F_0(p^2,m_1^2,m_2^2)&= A_0(m_1^2)-2 A_0(m_2^2) \nonumber \\&\quad - (2 p^2 + 2 m_1^2 - m_2^2) B_0(p^2,m_1^2,m_2^2) ,\end{aligned}$$

A.39 $$\begin{aligned} \dot{F}_0(p^2,m_1^2,m_2^2)&= - 2 B_0(p^2,m_1^2,m_2^2) \nonumber \\&\quad - (2 p^2 + 2 m_1^2 - m_2^2) \dot{B}_0(p^2,m_1^2,m_2^2). \end{aligned}$$

(f) *S* diagram

A.40 $$\begin{aligned} \Pi ^{(f)}_{SS} =\,&- c^1 A_0(m_1^2) ,\end{aligned}$$

A.41 $$\begin{aligned} \dot{\Pi }^{(f)}_{SS} =\,&0. \end{aligned}$$

(g) *V* diagram

A.42 $$\begin{aligned} \Pi ^{(f)}_{SS} = \,&c^1 \left( A_0(m_1^2) - \frac{1}{2} r m_1^2\right) ,\end{aligned}$$

A.43 $$\begin{aligned} \dot{\Pi }^{(f)}_{SS} =\,&0. \end{aligned}$$

#### A.1.3: Gauge boson

(a) *FF* diagram

A.44 $$\begin{aligned} \Pi ^{(a)}_{VV} =\,&(c^1_L c^2_L + c^1_R c^2_R) H_0(p^2,m_1^2,m_2^2) \nonumber \\&+ 4(c^1_L c^2_R + c^1_R c^2_L) B_0(p^2,m_1^2,m_2^2) ,\end{aligned}$$

A.45 $$\begin{aligned} \dot{\Pi }^{(a)}_{VV} =\,&(c^1_L c^2_L + c^1_R c^2_R) \dot{H}_0(p^2,m_1^2,m_2^2) \nonumber \\&+ 4(c^1_L c^2_R + c^1_R c^2_L)\dot{B}_0(p^2,m_1^2,m_2^2) \end{aligned}$$

with

A.46 $$\begin{aligned} H_0(p^2,m_1^2,m_2^2) =\,&4 B_{00}(p^2,m_1^2,m_2^2) - A_0(m_1^2) - A_0(m_2^2)\nonumber \\&+ (p^2 - m_1^2 - m_2^2) B_0(p^2,m_1^2,m_2^2) ,\end{aligned}$$

A.47 $$\begin{aligned} \dot{H}_0(p^2,m_1^2,m_2^2) =\,&4 \dot{B}_{00}(p^2,m_1^2,m_2^2)\nonumber \\&+ (p^2 - m_1^2 - m_2^2) \dot{B}_0(p^2,m_1^2,m_2^2)\nonumber \\&+ B_0(p^2,m_1^2,m_2^2). \end{aligned}$$

(b) *SS* diagram

A.48 $$\begin{aligned} \Pi ^{(b)}_{VV} =\,&-4 c^1 c^2 B_{00}(p^2,m_1^2,m_2^2) ,\end{aligned}$$

A.49 $$\begin{aligned} \dot{\Pi }^{(b)}_{VV} =\,&-4 c^1 c^2 \dot{B}_{00}(p^2,m_1^2,m_2^2). \end{aligned}$$

(c) *UU* diagram

A.50 $$\begin{aligned} \Pi ^{(c)}_{VV} =\,&c^1 c^2 B_{00}(p^2,m_1^2,m_2^2) ,\end{aligned}$$

A.51 $$\begin{aligned} \dot{\Pi }^{(c)}_{VV} =\,&c^1 c^2 \dot{B}_{00}(p^2,m_1^2,m_2^2). \end{aligned}$$

(d) *VV* diagram

A.52 $$\begin{aligned} \Pi ^{(d)}_{VV} =\,&-c^1 c^2 V_0(p^2,m_1^2,m_2^2) ,\end{aligned}$$

A.53 $$\begin{aligned} \dot{\Pi }^{(d)}_{VV} =\,&- c^1 c^2 \dot{V}_0(p^2,m_1^2,m_2^2) \end{aligned}$$

with

A.54 $$\begin{aligned} V_0(p^2,m_1^2,m_2^2) =\,&10 B_{00}(p^2,m_1^2,m_2^2) \nonumber \\&+ (m_1^2+m_2^2 + 4p^2) B_0(p^2,m_1^2,m_2^2) \nonumber \\&+ A_0(m_1^2) + A_0(m_2^2) \nonumber \\&- 2r \left( m_1^2-m_2^2 - \frac{1}{3} p^2 \right) ,\end{aligned}$$

A.55 $$\begin{aligned} \dot{V}_0(p^2,m_1^2,m_2^2) =\,&10 \dot{B}_{00}(p^2,m_1^2,m_2^2) \nonumber \\&+ (m_1^2+m_2^2 + 4p^2) \dot{B}_0(p^2,m_1^2,m_2^2) \nonumber \\&+ 4 B_0(p^2,m_1^2,m_2^2). \end{aligned}$$

(e) *SV* diagram

A.56 $$\begin{aligned} \Pi ^{(e)}_{VV} =\,&c^1 c^2 B_0(p^2,m_1^2,m_2^2) ,\end{aligned}$$

A.57 $$\begin{aligned} \dot{\Pi }^{(e)}_{VV} =\,&c^1 c^2 \dot{B}_0(p^2,m_1^2,m_2^2). \end{aligned}$$

(f) *S* diagram

A.58 $$\begin{aligned} \Pi ^{(f)}_{VV} =\,&c^1 A_0(m_1^2) ,\end{aligned}$$

A.59 $$\begin{aligned} \dot{\Pi }^{(f)}_{VV} =\,&0. \end{aligned}$$

(g) *V* diagram

A.60 $$\begin{aligned} \Pi ^{(f)}_{VV} =\,&- (4 c^1_1 + c^1_2 + c^1_3) A_0(m_1^2) + 2 m_1^2 c^1_1 r ,\end{aligned}$$

A.61 $$\begin{aligned} \dot{\Pi }^{(f)}_{VV} =\,&0. \end{aligned}$$

### A.2: Vertex corrections

#### A.2.1: Fermion to fermion and scalar decays

(a) *SFF* diagram

A.62 $$\begin{aligned} M^{(a)}_1 =\,&i \Big [B_0 c^1_R c^2_R c^3_L + C_1 c^1_R c^2_R c^3_L p_0^2 - C_2 c^1_L c^2_L c^3_R p_0 p_1 \nonumber \\&- C_0 c^1_R c^2_R c^3_L p_1^2 - C_1 c^1_R c^2_R c^3_L p_1^2 \nonumber \\&- C_2 c^1_R c^2_R c^3_L p_1^2 + C_0 c^1_R c^2_R c^3_L m_1^2\nonumber \\&+ C_1 c^1_L c^2_R c^3_L p_0 m_2 - C_1 c^1_R c^2_L c^3_R p_1 m_2 \nonumber \\&- C_2 c^1_R c^2_L c^3_R p_1 m_2 +(C_1 c^1_L c^2_R c^3_R p_0 \nonumber \\&- (C_1 + C_2) c^1_R c^2_L c^3_L p_1 \nonumber \\&+ C_0 (c^1_L c^2_R c^3_R p_0 - c^1_R c^2_L c^3_L p_1 + c^1_R c^2_R c^3_R m_2)) m_3\Big ] ,\end{aligned}$$

A.63 $$\begin{aligned} M^{(a)}_2 =\,&M^{(a)}_1 | L \leftrightarrow R, \end{aligned}$$

with $$B_i = B_i(p_2^2, m_2^2, m_3^2)$$ and $$C_i = C_i(p_2^2, p_0^2, p_1^2, m_3^2, m_2^2, m_1^2)$$.

(b) *FSS* diagram

A.64 $$\begin{aligned} M^{(b)}_1 =\,&-i c^3 (C_2 c^1_L c^2_R p_0 + C_1 c^1_R c^2_L p_1 - C_0 c^1_R c^2_R m_1) ,\end{aligned}$$

A.65 $$\begin{aligned} M^{(b)}_2 =\,&M^{(b)}_1 | L \leftrightarrow R, \end{aligned}$$

with $$C_i = C_i(p_1^2, p_2^2, p_0^2, m_1^2, m_3^2, m_2^2)$$.

(c) *VFF* diagram

A.66 $$\begin{aligned} M^{(c)}_1 =\,&-2 i \Big [2 B_0 c^1_R c^2_L c^3_R - c^1_L c^2_L p_0 (C_1 c^3_R m_2 \nonumber \\&+ (C_0 + C_1) c^3_L m_3) \nonumber \\&+ c^1_R (- c^2_L c^3_R (r + 2 C_1 (- p_0^2 + p_1^2) \nonumber \\&+ C_2 (p_0^2 + 3 p_1^2 - p_2^2)\nonumber \\&+ 2 C_0 (p_1 - m_1) (p_1 + m_1)) \nonumber \\&+ 2 C_0 c^2_L c^3_L m_2 m_3 + c^2_R p_1 ((C_1 + C_2) c^3_L m_2 \nonumber \\&+ (C_0 + C_1 + C_2) c^3_R m_3))\Big ] ,\end{aligned}$$

A.67 $$\begin{aligned} M^{(c)}_2 =\,&M^{(c)}_1 | L \leftrightarrow R, \end{aligned}$$

with $$B_i = B_i(p_2^2, m_2^2, m_3^2)$$ and $$C_i = C_i(p_2^2, p_0^2, p_1^2, m_3^2, m_2^2, m_1^2)$$.

(d) *FSV* diagram

A.68 $$\begin{aligned} M^{(d)}_1 =\,&-i c^3 \Big [B_0 c^1_R c^2_L + 2 C_1 c^1_R c^2_L p_0^2 + 2 C_2 c^1_R c^2_L p_0^2 \nonumber \\&-2 C_1 c^1_L c^2_R p_0 p_1 - C_2 c^1_L c^2_R p_0 p_1 + C_1 c^1_R c^2_L p_1^2\nonumber \\&-2 C_1 c^1_R c^2_L p_2^2 - ((2 C_0 + C_2) c^1_L c^2_L p_0 \nonumber \\&+ (- C_0 + C_1) c^1_R c^2_R p_1) m_1 + C_0 c^1_R c^2_L m_1^2\Big ] ,\end{aligned}$$

A.69 $$\begin{aligned} M^{(d)}_2 =\,&M^{(d)}_1 | L \leftrightarrow R, \end{aligned}$$

with $$B_i = B_i(p_2^2, m_2^2, m_3^2)$$ and $$C_i = C_i(p_1^2, p_2^2, p_0^2, m_1^2, m_3^2, m_2^2)$$.

(e) *FVS* diagram

A.70 $$\begin{aligned} M^{(e)}_1 =\,&i c^3 \Big [B_0 c^1_R c^2_R + C_2 c^1_R c^2_R p_0^2 - C_1 c^1_L c^2_L p_0 p_1 \nonumber \\&-2 C_2 c^1_L c^2_L p_0 p_1 + 2 C_1 c^1_R c^2_R p_1^2 + 2 C_2 c^1_R c^2_R p_1^2 \nonumber \\&-2 C_2 c^1_R c^2_R p_2^2 - ((- C_0 + C_2) c^1_L c^2_R p_0 \nonumber \\&+ (2 C_0 + C_1) c^1_R c^2_L p_1) m_1 + C_0 c^1_R c^2_R m_1^2\Big ] ,\end{aligned}$$

A.71 $$\begin{aligned} M^{(e)}_2 =\,&M^{(e)}_1 | L \leftrightarrow R, \end{aligned}$$

with $$B_i = B_i(p_2^2, m_3^2, m_2^2)$$ and $$C_i = C_i(p_1^2, p_2^2, p_0^2, m_1^2, m_3^2, m_2^2)$$.

(f) *FVV* diagram

A.72 $$\begin{aligned} M^{(f)}_1 =\,&2 i c^3 (C_2 c^1_L c^2_L p_0 + C_1 c^1_R c^2_R p_1 + 2 C_0 c^1_R c^2_L m_1) ,\end{aligned}$$

A.73 $$\begin{aligned} M^{(f)}_2 =\,&M^{(f)}_1 | L \leftrightarrow R, \end{aligned}$$

with $$C_i = C_i(p_1^2, p_2^2, p_0^2, m_1^2, m_3^2, m_2^2)$$.

#### A.2.2: Fermion to fermion and gauge boson decays

(a) *SFF* diagram

A.74 $$\begin{aligned} M^{(a)}_1 =\,&-i \Big [B_0 c^1_R c^2_L c^3_R -2 C_{00} c^1_R c^2_L c^3_R - C_0 c^1_R c^2_L c^3_R p_0^2 \nonumber \\&- C_1 c^1_R c^2_L c^3_R p_0^2 - C_2 c^1_R c^2_L c^3_R p_0^2 \nonumber \\&+ C_2 c^1_L c^2_R c^3_L p_0 p_1 + C_1 c^1_R c^2_L c^3_R p_1^2 + C_0 c^1_R c^2_L c^3_R m_1^2\nonumber \\&- C_0 c^1_L c^2_L c^3_R p_0 m_2 \nonumber \\&- C_1 c^1_L c^2_L c^3_R p_0 m_2 - C_2 c^1_L c^2_L c^3_R p_0 m_2 \nonumber \\&- C_0 c^1_R c^2_R c^3_L p_1 m_2 - C_1 c^1_R c^2_R c^3_L p_1 m_2 \nonumber \\&+ (C_1 c^1_L c^2_L c^3_L p_0 + C_2 c^1_L c^2_L c^3_L p_0 \nonumber \\&+ C_1 c^1_R c^2_R c^3_R p_1 \nonumber \\&- C_0 c^1_R c^2_L c^3_L m_2) m_3\Big ] ,\end{aligned}$$

A.75 $$\begin{aligned} M^{(a)}_2 =\,&M^{(a)}_1 | L \leftrightarrow R ,\end{aligned}$$

A.76 $$\begin{aligned} M^{(a)}_3 =\,&2 i \Big [C_{12} (- c^1_L c^2_R c^3_L p_0 + c^1_R c^2_L c^3_R p_1) \nonumber \\&+ c^2_R (- C_{22} c^1_L c^3_L p_0 + (C_0 + C_1) c^1_R c^3_L m_2 \nonumber \\&+ C_2 c^3_L (- c^1_L p_0 + c^1_R m_2) - C_1 c^1_R c^3_R m_3)\Big ] ,\end{aligned}$$

A.77 $$\begin{aligned} M^{(a)}_4 =\,&M^{(a)}_3 | L \leftrightarrow R, \end{aligned}$$

with $$B_i = B_i(p_2^2, m_2^2, m_3^2)$$ and $$C_i = C_i(p_2^2, p_1^2, p_0^2, m_2^2, m_3^2, m_1^2)$$.

(b) *FSS* diagram

A.78 $$\begin{aligned} M^{(b)}_1 =\,&2 i C_{00} c^1_R c^2_L c^3 ,\end{aligned}$$

A.79 $$\begin{aligned} M^{(b)}_2 =\,&M^{(b)}_1 | L \leftrightarrow R ,\end{aligned}$$

A.80 $$\begin{aligned} M^{(b)}_3 =\,&-2 i c^3 \Big [(C_2 + C_{22}) c^1_L c^2_R p_0 \nonumber \\&+ (C_1 + C_{11}) c^1_R c^2_L p_1 \nonumber \\&+ C_{12} (c^1_L c^2_R p_0 + c^1_R c^2_L p_1) \nonumber \\&- (C_0 + C_1 + C_2) c^1_R c^2_R m_1\Big ] ,\end{aligned}$$

A.81 $$\begin{aligned} M^{(b)}_4 =\,&M^{(b)}_3 | L \leftrightarrow R, \end{aligned}$$

with $$C_i = C_i(p_1^2, p_2^2, p_0^2, m_1^2, m_3^2, m_2^2)$$.

(c) *VFF* diagram

A.82 $$\begin{aligned} M^{(c)}_1 =\,&-i \Big [2 B_0 c^1_L c^2_L c^3_L -4 C_{00} c^1_L c^2_L c^3_L - c^1_L c^2_L c^3_L r \nonumber \\&-2 C_0 c^1_L c^2_L c^3_L p_0^2 -2 C_1 c^1_L c^2_L c^3_L p_0^2 \nonumber \\&-4 C_2 c^1_L c^2_L c^3_L p_0^2 -2 C_2 c^1_R c^2_R c^3_R p_0 p_1 \nonumber \\&+ 2 C_1 c^1_L c^2_L c^3_L p_1^2 -2 C_2 c^1_L c^2_L c^3_L p_1^2 \nonumber \\&+ 2 C_2 c^1_L c^2_L c^3_L p_2^2 + 2 C_0 c^1_L c^2_L c^3_L m_1^2 \nonumber \\&-2 C_0 c^1_L c^2_L c^3_R m_2 m_3\Big ] ,\end{aligned}$$

A.83 $$\begin{aligned} M^{(c)}_2 =\,&M^{(c)}_1 | L \leftrightarrow R ,\end{aligned}$$

A.84 $$\begin{aligned} M^{(c)}_3 =\,&-4 i \Big [C_{22} c^1_R c^2_R c^3_R p_0\! + \!C_{12} (c^1_R c^2_R c^3_R p_0 \!-\! c^1_L c^2_L c^3_L p_1)\nonumber \\&+ C_2 c^1_L (- c^2_L c^3_L p_1 + c^2_R c^3_R m_2 + c^2_R c^3_L m_3)\Big ] ,\end{aligned}$$

A.85 $$\begin{aligned} M^{(c)}_4 =\,&M^{(c)}_3 | L \leftrightarrow R, \end{aligned}$$

with $$B_i = B_i(p_2^2, m_2^2, m_3^2)$$ and $$C_i = C_i(p_2^2, p_1^2, p_0^2, m_2^2, m_3^2, m_1^2)$$.

(d) *FSV* diagram

A.86 $$\begin{aligned} M^{(d)}_1 =\,&-i c^3 (- C_2 c^1_L c^2_L p_0 + C_1 c^1_R c^2_R p_1 + C_0 c^1_R c^2_L m_1) ,\end{aligned}$$

A.87 $$\begin{aligned} M^{(d)}_2 =\,&M^{(d)}_1 | L \leftrightarrow R ,\end{aligned}$$

A.88 $$\begin{aligned} M^{(d)}_3 =\,&-2 i C_1 c^1_R c^2_R c^3 ,\end{aligned}$$

A.89 $$\begin{aligned} M^{(d)}_4 =\,&M^{(d)}_3 | L \leftrightarrow R, \end{aligned}$$

with $$C_i = C_i(p_1^2, p_2^2, p_0^2, m_1^2, m_3^2, m_2^2)$$.

(e) *FVS* diagram

A.90 $$\begin{aligned} M^{(e)}_1 =\,&-i c^3 (C_2 c^1_R c^2_L p_0 - C_1 c^1_L c^2_R p_1 + C_0 c^1_L c^2_L m_1) ,\end{aligned}$$

A.91 $$\begin{aligned} M^{(e)}_2 =\,&M^{(e)}_1 | L \leftrightarrow R ,\end{aligned}$$

A.92 $$\begin{aligned} M^{(e)}_3 =\,&-2 i C_2 c^1_L c^2_R c^3 ,\end{aligned}$$

A.93 $$\begin{aligned} M^{(e)}_4 =\,&M^{(e)}_3 | L \leftrightarrow R, \end{aligned}$$

with $$C_i = C_i(p_1^2, p_2^2, p_0^2, m_1^2, m_3^2, m_2^2)$$.

(f) *FVV* diagram

A.94 $$\begin{aligned} M^{(f)}_1 =\,&i c^3 \Big [2 B_0 c^1_L c^2_L + 4 C_{00} c^1_L c^2_L - c^1_L c^2_L r + 2 C_1 c^1_L c^2_L p_0^2 \nonumber \\&+ 3 C_2 c^1_L c^2_L p_0^2 + 3 C_1 c^1_R c^2_R p_0 p_1 \nonumber \\&+ 3 C_2 c^1_R c^2_R p_0 p_1 + 3 C_1 c^1_L c^2_L p_1^2 + 2 C_2 c^1_L c^2_L p_1^2 \nonumber \\&-2 C_1 c^1_L c^2_L p_2^2 -2 C_2 c^1_L c^2_L p_2^2 \nonumber \\&+ 3 C_0 \left( c^1_R c^2_L p_0 + c^1_L c^2_R p_1\right) m_1 + 2 C_0 c^1_L c^2_L m_1^2\Big ] ,\end{aligned}$$

A.95 $$\begin{aligned} M^{(f)}_2 =\,&M^{(f)}_1 | L \leftrightarrow R ,\end{aligned}$$

A.96 $$\begin{aligned} M^{(f)}_3 =\,&-2 i c^3 \Big [- C_1 c^1_R c^2_R p_0 + 2 C_{12} c^1_R c^2_R p_0 \nonumber \\&+ 2 C_{22} c^1_R c^2_R p_0 + 2 C_{11} c^1_L c^2_L p_1 + 2 C_{12} c^1_L c^2_L p_1\nonumber \\&- C_2 c^1_L c^2_L p_1 + 3 (C_1 + C_2) c^1_L c^2_R m_1\Big ] ,\end{aligned}$$

A.97 $$\begin{aligned} M^{(f)}_4 =\,&M^{(f)}_3 | L \leftrightarrow R, \end{aligned}$$

with $$B_i = B_i(p_2^2, m_2^2, m_3^2)$$ and $$C_i = C_i(p_1^2, p_2^2, p_0^2, m_1^2, m_3^2, m_2^2)$$.

#### A.2.3: Scalar to two fermion decays

(a) *FFS* diagram

A.98 $$\begin{aligned} M^{(a)}_1 =\,&i \Big [B_0 c^1_L c^2_R c^3_R + C_2 c^1_L c^2_R c^3_R p_1^2 - C_2 c^1_R c^2_L c^3_L p_1 p_2 \nonumber \\&- C_0 c^1_L c^2_L c^3_R p_1 m_1 - C_2 c^1_L c^2_L c^3_R p_1 m_1 \nonumber \\&+ C_0 c^1_R c^2_R c^3_L p_2 m_1 + C_0 c^1_L c^2_R c^3_R m_1^2 \nonumber \\&- C_2 c^1_R c^2_L c^3_R p_1 m_2 + C_0 c^1_R c^2_R c^3_R m_1 m_2 \nonumber \\&+ C_1 \left( c^1_L c^2_R c^3_R p_0^2 - c^1_L c^2_L c^3_R p_1 m_1 + c^1_R c^2_R c^3_L p_2 m_1 \right. \nonumber \\&\left. - c^1_R c^2_L c^3_R p_1 m_2 + c^1_L c^2_R c^3_L p_2 m_2\right) \Big ] ,\end{aligned}$$

A.99 $$\begin{aligned} M^{(a)}_2 =\,&M^{(a)}_1 | L \leftrightarrow R, \end{aligned}$$

with $$B_i = B_i(p_2^2, m_2^2, m_3^2)$$ and $$C_i = C_i(p_0^2, p_2^2, p_1^2, m_1^2, m_2^2, m_3^2)$$.

(b) *SSF* diagram

A.100 $$\begin{aligned} M^{(b)}_1 =\,&-i c^1 (C_1 c^2_L c^3_R p_1 + C_2 c^2_R c^3_L p_2 - C_0 c^2_R c^3_R m_3) ,\end{aligned}$$

A.101 $$\begin{aligned} M^{(b)}_2 =\,&M^{(b)}_1 | L \leftrightarrow R, \end{aligned}$$

with $$C_i = C_i(p_1^2, p_0^2, p_2^2, m_3^2, m_1^2, m_2^2)$$.

(c) *FFV* diagram

A.102 $$\begin{aligned} M^{(c)}_1 =\,&-2 i \Big [2 B_0 c^1_R c^2_L c^3_R - (C_0 + C_1) c^1_L c^2_L c^3_L p_2 m_1 \nonumber \\&+ c^1_L c^3_R \left( (C_1 + C_2) c^2_R p_1 + 2 C_0 c^2_L m_1\right) m_2 \nonumber \\&+ c^1_R \left( (C_0 + C_1 + C_2) c^2_R c^3_R p_1 m_1 \right. \nonumber \\&+ c^2_L \left( c^3_R \left( - r + 2 C_1 p_0^2 + C_2 \left( p_0^2 + p_1^2 - p_2^2\right) \right. \right. \nonumber \\&\left. \left. \left. + 2 C_0 m_1^2\right) - C_1 c^3_L p_2 m_2\right) \right) \Big ] ,\end{aligned}$$

A.103 $$\begin{aligned} M^{(c)}_2 =\,&M^{(c)}_1 | L \leftrightarrow R, \end{aligned}$$

with $$B_i = B_i(p_2^2, m_2^2, m_3^2)$$ and $$C_i = C_i(p_0^2, p_2^2, p_1^2, m_1^2, m_2^2, m_3^2)$$.

(d) *SVF* diagram

A.104 $$\begin{aligned} M^{(d)}_1 =\,&i c^1 \Big [B_0 c^2_R c^3_R - C_2 c^2_R c^3_R p_0^2 - C_0 c^2_R c^3_R p_1^2 \nonumber \\&+ C_2 c^2_R c^3_R p_1^2 - C_1 c^2_L c^3_L p_1 p_2 -2 C_2 c^2_L c^3_L p_1 p_2\nonumber \\&+ C_0 c^2_R c^3_R m_1^2 - \left( \left( 2 C_0 + C_1\right) c^2_L c^3_R p_1 \right. \nonumber \\&\left. + \left( - C_0 + C_2\right) c^2_R c^3_L p_2\right) m_3\Big ] ,\end{aligned}$$

A.105 $$\begin{aligned} M^{(d)}_2 =\,&M^{(d)}_1 | L \leftrightarrow R, \end{aligned}$$

with $$B_i = B_i(p_2^2, m_3^2, m_2^2)$$ and $$C_i = C_i(p_1^2, p_0^2, p_2^2, m_3^2, m_1^2, m_2^2)$$.

(e) *VSF* diagram

A.106 $$\begin{aligned} M^{(e)}_1 =\,&-i c^1 \Big [B_0 c^2_L c^3_R + C_2 c^2_L c^3_R p_0^2 - C_0 c^2_L c^3_R p_1^2 \nonumber \\&- C_2 c^2_L c^3_R p_1^2- C_2 c^2_R c^3_L p_1 p_2 + C_2 c^2_L c^3_R p_2^2 \nonumber \\&+ C_0 c^2_L c^3_R m_1^2 + C_0 c^2_R c^3_R p_1 m_3 \nonumber \\&-2 C_0 c^2_L c^3_L p_2 m_3 - C_2 c^2_L c^3_L p_2 m_3 \nonumber \\&- C_1 \left( c^2_L c^3_R \left( 2 p_0^2 + p_1^2 -2 p_2^2\right) \right. \nonumber \\&\left. + c^2_R p_1 \left( 2 c^3_L p_2 + c^3_R m_3\right) \right) \Big ] ,\end{aligned}$$

A.107 $$\begin{aligned} M^{(e)}_2 =\,&M^{(e)}_1 | L \leftrightarrow R, \end{aligned}$$

with $$B_i = B_i(p_2^2, m_3^2, m_2^2)$$ and $$C_i = C_i(p_1^2, p_0^2, p_2^2, m_3^2, m_1^2, m_2^2)$$.

(f) *VVF* diagram

A.108 $$\begin{aligned} M^{(f)}_1 =\,&2 i c^1 (C_1 c^2_R c^3_R p_1 + C_2 c^2_L c^3_L p_2 + 2 C_0 c^2_L c^3_R m_3) ,\end{aligned}$$

A.109 $$\begin{aligned} M^{(f)}_2 =\,&M^{(f)}_1 | L \leftrightarrow R, \end{aligned}$$

with $$C_i = C_i(p_1^2, p_0^2, p_2^2, m_3^2, m_1^2, m_2^2)$$.

#### A.2.4: Scalar to two scalar decays

(a) *FFF* diagram

A.110 $$\begin{aligned} M^{(a)} =\,&i \Big [C_1 c^1_R c^2_R c^3_L p_0^2 m_1 + 3 C_2 c^1_R c^2_R c^3_L p_0^2 m_1 \nonumber \\&+ C_1 c^1_L c^2_L c^3_R p_0^2 m_1 \nonumber \\&+ 3 C_2 c^1_L c^2_L c^3_R p_0^2 m_1 + 3 C_1 c^1_R c^2_R c^3_L p_1^2 m_1 \nonumber \\&+ C_2 c^1_R c^2_R c^3_L p_1^2 m_1 + 3 C_1 c^1_L c^2_L c^3_R p_1^2 m_1 \nonumber \\&+ C_2 c^1_L c^2_L c^3_R p_1^2 m_1 - C_1 c^1_R c^2_R c^3_L p_2^2 m_1\nonumber \\&- C_2 c^1_R c^2_R c^3_L p_2^2 m_1 - C_1 c^1_L c^2_L c^3_R p_2^2 m_1\nonumber \\&- C_2 c^1_L c^2_L c^3_R p_2^2 m_1 + C_2 c^1_L c^2_R c^3_L p_0^2 m_2 \nonumber \\&+ C_2 c^1_R c^2_L c^3_R p_0^2 m_2 + 2 C_1 c^1_L c^2_R c^3_L p_1^2 m_2 \nonumber \\&+ C_2 c^1_L c^2_R c^3_L p_1^2 m_2 + 2 C_1 c^1_R c^2_L c^3_R p_1^2 m_2 \nonumber \\&+ C_2 c^1_R c^2_L c^3_R p_1^2 m_2 - C_2 c^1_L c^2_R c^3_L p_2^2 m_2\nonumber \\&- C_2 c^1_R c^2_L c^3_R p_2^2 m_2 + \left( c^1_R c^2_L c^3_L + c^1_L c^2_R c^3_R\right) \nonumber \\&\times \left( 2 C_2 p_0^2 + C_1 \left( p_0^2 + p_1^2 - p_2^2\right) \right) m_3 \nonumber \\&+ 2 B_0 \left( c^1_R \left( c^2_R c^3_L m_1 + c^2_L c^3_R m_2 + c^2_L c^3_L m_3\right) \right. \nonumber \\&\left. + c^1_L \left( c^2_L c^3_R m_1 + c^2_R c^3_L m_2 + c^2_R c^3_R m_3\right) \right) \nonumber \\&+ C_0 m_1 \left( c^1_R \left( c^2_R c^3_L \left( p_0^2 + p_1^2 - p_2^2 + 2 m_1^2\right) \right. \right. \nonumber \\&\left. \left. + 2 c^2_R c^3_R m_2 m_3 + 2 c^2_L m_1 \left( c^3_R m_2 + c^3_L m_3\right) \right) \right. \nonumber \\&+ c^1_L \left( c^2_L c^3_R \left( p_0^2 + p_1^2 - p_2^2 + 2 m_1^2\right) \right. \nonumber \\&\left. \left. + 2 c^2_L c^3_L m_2 m_3 + 2 c^2_R m_1 \left( c^3_L m_2 + c^3_R m_3\right) \right) \right) \Big ] \end{aligned}$$

with $$B_i = B_i(p_2^2, m_2^2, m_3^2)$$ and $$C_i = C_i(p_1^2, p_2^2, p_0^2, m_1^2, m_3^2, m_2^2)$$.

(b) *SSS* diagram

A.111 $$\begin{aligned} M^{(b)} =\,&-i C_0\left( p_1^2, p_2^2, p_0^2, m_1^2, m_3^2, m_2^2\right) c^1 c^2 c^3. \end{aligned}$$

(c) *UUU* diagram

A.112 $$\begin{aligned} M^{(c)} =\,&i C_0\left( p_1^2, p_2^2, p_0^2, m_1^2, m_3^2, m_2^2\right) c^1 c^2 c^3. \end{aligned}$$

(d) *VVV* diagram

A.113 $$\begin{aligned} M^{(d)} =\,&4 i C_0\left( p_1^2, p_2^2, p_0^2, m_1^2, m_3^2, m_2^2\right) c^1 c^2 c^3. \end{aligned}$$

(e) *SSV* diagram

A.114 $$\begin{aligned} M^{(e)} =\,&-i c^1 c^2 c^3 \Big [B_0 + (C_1 + C_2) (p^2_0 - p^2_1)\nonumber \\&+ ( C_2 - C_1) p_2^2 + C_0 \left( p_2^2 - p_0^2 + m_1^2\right) \Big ] \end{aligned}$$

with $$B_i = B_i(p_2^2, m_2^2, m_3^2)$$ and $$C_i = C_i(p_1^2, p_2^2, p_0^2, m_1^2, m_3^2, m_2^2)$$.

(f) *SVS* diagram

A.115 $$\begin{aligned} M^{(f)} =\,&-i c^1 c^2 c^3 \Big [B_0 - (C_1 + C_2) (p^2_0 - p^2_1)\nonumber \\&+ (C_1 - C_2) p_2^2 + C_0 \left( - p_1^2 + p_2^2 + m_1^2\right) \Big ] \end{aligned}$$

with $$B_i = B_i(p_2^2, m_3^2, m_2^2)$$ and $$C_i = C_i(p_1^2, p_2^2, p_0^2, m_1^2, m_3^2, m_2^2)$$.

(g) *VSS* diagram

A.116 $$\begin{aligned} M^{(g)} =\,&-i c^1 c^2 c^3 \Big [B_0 + C_1 p_0^2 + 3 C_2 p_0^2 + 3 C_1 p_1^2\nonumber \\&+ C_2 p_1^2 - (C_1 + C_2) p_2^2 \nonumber \\&+ C_0 \left( 2 \left( p_0^2 + p_1^2 - p_2^2\right) + m_1^2\right) \Big ] \end{aligned}$$

with $$B_i = B_i(p_2^2, m_2^2, m_3^2)$$ and $$C_i = C_i(p_1^2, p_2^2, p_0^2, m_1^2, m_3^2, m_2^2)$$.

(h) *SVV* diagram

A.117 $$\begin{aligned} M^{(h)} =\,&\frac{i}{2} c^1 c^2 c^3 \Big [2 B_0 - C_1 p_0^2 -3 C_2 p_0^2 -3 C_1 p_1^2\nonumber \\&- C_2 p_1^2 + (C_1 + C_2) p_2^2\nonumber \\&+ C_0 \left( p_0^2 + p_1^2 - p_2^2 + 2 m_1^2\right) \Big ] \end{aligned}$$

with $$B_i = B_i(p_2^2, m_2^2, m_3^2)$$ and $$C_i = C_i(p_1^2, p_2^2, p_0^2, m_1^2, m_3^2, m_2^2)$$.

(i) *VSV* diagram

A.118 $$\begin{aligned} M^{(i)} =\,&\frac{i}{2} c^1 c^2 c^3 \Big [2 B_0 + 2 C_1 \left( 2 p_0^2 + p_1^2 -2 p_2^2\right) \nonumber \\&+ C_2 \left( 7 p_0^2 - p_1^2 + p_2^2\right) \nonumber \\&+ 2 C_0 \left( 3 p_0^2 - p_1^2 + p_2^2 + m_1^2\right) \Big ] \end{aligned}$$

with $$B_i = B_i(p_2^2, m_2^2, m_3^2)$$ and $$C_i = C_i(p_1^2, p_2^2, p_0^2, m_1^2, m_3^2, m_2^2)$$.

(j) *VVS* diagram

A.119 $$\begin{aligned} M^{(j)} =\,&\frac{i}{2} c^1 c^2 c^3 \Big [2 B_0 - C_1 p_0^2 + 2 C_2 p_0^2 + 7 C_1 p_1^2 + 4 C_2 p_1^2 \nonumber \\&+ (C_1\! -\!4 C_2) p_2^2 \!+\! 2 C_0 \left( - p_0^2 \!+\! 3 p_1^2 + p_2^2 + m_1^2\right) \Big ] \end{aligned}$$

with $$B_i = B_i(p_2^2, m_3^2, m_2^2)$$ and $$C_i = C_i(p_1^2, p_2^2, p_0^2, m_1^2, m_3^2, m_2^2)$$.

(k) *SS* diagram

A.120 $$\begin{aligned} M^{(k)} =\,&\frac{i}{2} B_0\left( p_0^2, m_1^2, m_2^2\right) c^1 c^2. \end{aligned}$$

(l) *VV* diagram

A.121 $$\begin{aligned} M^{(l)} =\,&i c^1 c^2 \left( 2 B_0\left( p_0^2, m_1^2, m_2^2\right) - r\right) . \end{aligned}$$

(m) *SS* diagram

A.122 $$\begin{aligned} M^{(m)} =\,&\frac{i}{2} B_0\left( p_1^2, m_1^2, m_2^2\right) c^1 c^2. \end{aligned}$$

(n) *VV* diagram

A.123 $$\begin{aligned} M^{(n)} =\,&i c^1 c^2 \left( 2 B_0\left( p_1^2, m_1^2, m_2^2\right) - r\right) . \end{aligned}$$

(o) *SS* diagram

A.124 $$\begin{aligned} M^{(o)} =\,&\frac{i}{2} B_0\left( p_2^2, m_1^2, m_2^2\right) c^1 c^3. \end{aligned}$$

(p) *VV* diagram

A.125 $$\begin{aligned} M^{(p)} =\,&i c^1 c^3 \left( 2 B_0\left( p_2^2, m_1^2, m_2^2\right) - r\right) . \end{aligned}$$

#### A.2.5: Scalar to scalar and gauge boson decays

(a) *FFF* diagram

A.126 $$\begin{aligned} M^{(a)} =\,&-2 i \Big [B_0 c^1_L c^2_R c^3_L + B_0 c^1_R c^2_L c^3_R \nonumber \\&+ C_2 c^1_L c^2_R c^3_L p_0^2 + C_2 c^1_R c^2_L c^3_R p_0^2 \nonumber \\&+ C_1 c^1_L c^2_R c^3_L p_1^2 + C_1 c^1_R c^2_L c^3_R p_1^2 \nonumber \\&+ 2 C_0 c^1_L c^2_R c^3_L m_1^2+ C_1 c^1_L c^2_R c^3_L m_1^2 \nonumber \\&+ C_2 c^1_L c^2_R c^3_L m_1^2 + 2 C_0 c^1_R c^2_L c^3_R m_1^2 \nonumber \\&+ C_1 c^1_R c^2_L c^3_R m_1^2+ C_2 c^1_R c^2_L c^3_R m_1^2 \nonumber \\&+ C_0 c^1_R c^2_R c^3_L m_1 m_2 + C_1 c^1_R c^2_R c^3_L m_1 m_2 \nonumber \\&+ C_2 c^1_R c^2_R c^3_L m_1 m_2+ C_0 c^1_L c^2_L c^3_R m_1 m_2\nonumber \\&+ C_1 c^1_L c^2_L c^3_R m_1 m_2 + C_2 c^1_L c^2_L c^3_R m_1 m_2 \nonumber \\&+ \left( \left( C_0 + C_1 + C_2\right) \left( c^1_L c^2_L c^3_L + c^1_R c^2_R c^3_R\right) m_1 \right. \nonumber \\&\left. + \left( C_1 + C_2\right) \left( c^1_R c^2_L c^3_L + c^1_L c^2_R c^3_R\right) m_2\right) m_3\Big ] \end{aligned}$$

with $$B_i = B_i(p_2^2, m_2^2, m_3^2)$$ and $$C_i = C_i(p_1^2, p_2^2, p_0^2, m_1^2, m_3^2, m_2^2)$$.

(b) *SSS* diagram

A.127 $$\begin{aligned} M^{(b)} =\,&-2 i \left( C_0 + C_1 + C_2\right) c^1 c^2 c^3 \end{aligned}$$

with $$C_i = C_i(p_1^2, p_2^2, p_0^2, m_1^2, m_3^2, m_2^2)$$.

(c) *UUU* diagram

A.128 $$\begin{aligned} M^{(c)} =\,&i \left( C_0 + C_1 + C_2\right) c^1 c^2 c^3 \end{aligned}$$

with $$C_i = C_i(p_1^2, p_2^2, p_0^2, m_1^2, m_3^2, m_2^2)$$.

(d) *VVV* diagram

A.129 $$\begin{aligned} M^{(d)} =\,&-6 i \left( C_0 + C_1 + C_2\right) c^1 c^2 c^3 \end{aligned}$$

with $$C_i = C_i(p_1^2, p_2^2, p_0^2, m_1^2, m_3^2, m_2^2)$$.

(e) *SSV* diagram

A.130 $$\begin{aligned} M^{(e)} =\,&-i \left( C_0 - C_1 - C_2\right) c^1 c^2 c^3 \end{aligned}$$

with $$C_i = C_i(p_1^2, p_2^2, p_0^2, m_1^2, m_3^2, m_2^2)$$.

(f) *SVS* diagram

A.131 $$\begin{aligned} M^{(f)} =\,&i \left( C_0 - C_1 - C_2\right) c^1 c^2 c^3 \end{aligned}$$

with $$C_i = C_i(p_1^2, p_2^2, p_0^2, m_1^2, m_3^2, m_2^2)$$.

(g) *VSS* diagram

A.132 $$\begin{aligned} M^{(g)} =\,&-2 i c^1 c^2 c^3 \Big [4 C_{00} + 5 C_2 p_0^2 + 3 C_{22} p_0^2 + 3 C_2 p_1^2 \nonumber \\&+ C_{22} p_1^2 + 4 C_{12} (p_0^2 + p_1^2) \nonumber \\&-2 C_{12} p_2^2 -3 C_2 p_2^2 - C_{22} p_2^2 \nonumber \\&+ \left( C_0 + C_1 + C_2\right) m_1^2 \nonumber \\&+ 2 C_0 p_0^2 + 3 C_1 p_0^2 + C_{11} p_0^2 \nonumber \\&+ 2 C_0 p_1^2 + 5 C_1 p_1^2 + 3 C_{11} p_1^2 \nonumber \\&- \left( 2 C_0 + 3 C_1 + C_{11}\right) p_2^2\Big ] \end{aligned}$$

with $$C_i = C_i(p_1^2, p_2^2, p_0^2, m_1^2, m_3^2, m_2^2)$$.

(h) *SVV* diagram

A.133 $$\begin{aligned} M^{(h)} =&\, -i c^1 c^2 c^3 \Big [ 4 B_0 -4 C_{00} + C_1 p_0^2 -4 C_{12} p_0^2 \nonumber \\&- C_2 p_0^2 -3 C_{22} p_0^2 - C_1 p_1^2 \nonumber \\&-4 C_{12} p_1^2 + C_2 p_1^2 - C_{22} p_1^2 \nonumber \\&+ \left( -2 \left( C_1 - C_{12} + C_2\right) + C_{22}\right) p_2^2 \nonumber \\&+ C_0 \left( p_2^2 + 4 m_1^2\right) - C_{11} \left( p_0^2 + 3 p_1^2 - p_2^2\right) \Big ] \end{aligned}$$

with $$B_i = B_i(p_2^2, m_2^2, m_3^2)$$ and $$C_i = C_i(p_1^2, p_2^2, p_0^2, m_1^2, m_3^2, m_2^2)$$.

(i) *VSV* diagram

A.134 $$\begin{aligned} M^{(i)} =\,&-i \left( 2 C_0 + C_1 + C_2\right) c^1 c^2 c^3 \end{aligned}$$

with $$C_i = C_i(p_1^2, p_2^2, p_0^2, m_1^2, m_3^2, m_2^2)$$.

(j) *VVS* diagram

A.135 $$\begin{aligned} M^{(j)} =\,&i \left( 2 C_0 + C_1 + C_2\right) c^1 c^2 c^3 \end{aligned}$$

with $$C_i = C_i(p_1^2, p_2^2, p_0^2, m_1^2, m_3^2, m_2^2)$$.

(k) *SV* diagram

A.136 $$\begin{aligned} M^{(k)} =\,&-i c^1 c^2 (B_0 - B_1) \end{aligned}$$

with $$B_i = B_i(p_0^2, m_1^2, m_2^2)$$.

(l) *SV* diagram

A.137 $$\begin{aligned} M^{(l)} =\,&i c^1 c^2 (B_0 - B_1) \end{aligned}$$

with $$B_i = B_i(p_1^2, m_1^2, m_2^2)$$.

(m) *SS* diagram

A.138 $$\begin{aligned} M^{(m)} = 0. \end{aligned}$$

(n) *VV* diagram

A.139 $$\begin{aligned} M^{(n)} = 0. \end{aligned}$$

#### A.2.6: Scalar to two gauge boson decays

(a) *FFF* diagram

A.140 $$\begin{aligned} M^{(a)}_1 =\,&-i \Big [2 B_0 c^1_L c^2_L c^3_L m_1 + 2 B_0 c^1_R c^2_R c^3_R m_1 \nonumber \\&+ C_0 c^1_L c^2_L c^3_L p_0^2 m_1+ C_1 c^1_L c^2_L c^3_L p_0^2 m_1 \nonumber \\&+ 3 C_2 c^1_L c^2_L c^3_L p_0^2 m_1 + C_0 c^1_R c^2_R c^3_R p_0^2 m_1 \nonumber \\&+ C_1 c^1_R c^2_R c^3_R p_0^2 m_1+ 3 C_2 c^1_R c^2_R c^3_R p_0^2 m_1 \nonumber \\&+ C_0 c^1_L c^2_L c^3_L p_1^2 m_1 + 3 C_1 c^1_L c^2_L c^3_L p_1^2 m_1 \nonumber \\&+ C_2 c^1_L c^2_L c^3_L p_1^2 m_1+ C_0 c^1_R c^2_R c^3_R p_1^2 m_1 \nonumber \\&+ 3 C_1 c^1_R c^2_R c^3_R p_1^2 m_1 + C_2 c^1_R c^2_R c^3_R p_1^2 m_1 \nonumber \\&- C_0 c^1_L c^2_L c^3_L p_2^2 m_1- C_1 c^1_L c^2_L c^3_L p_2^2 m_1\nonumber \\&- C_2 c^1_L c^2_L c^3_L p_2^2 m_1 - C_0 c^1_R c^2_R c^3_R p_2^2 m_1 \nonumber \\&- C_1 c^1_R c^2_R c^3_R p_2^2 m_1- C_2 c^1_R c^2_R c^3_R p_2^2 m_1 \nonumber \\&+ 2 C_0 c^1_L c^2_L c^3_L m_1^3 + 2 C_0 c^1_R c^2_R c^3_R m_1^3 \nonumber \\&+ 2 B_0 c^1_R c^2_L c^3_L m_2+ 2 B_0 c^1_L c^2_R c^3_R m_2 \nonumber \\&+ C_2 c^1_R c^2_L c^3_L p_0^2 m_2 + C_2 c^1_L c^2_R c^3_R p_0^2 m_2 \nonumber \\&+ 2 C_1 c^1_R c^2_L c^3_L p_1^2 m_2+ C_2 c^1_R c^2_L c^3_L p_1^2 m_2 \nonumber \\&+ 2 C_1 c^1_L c^2_R c^3_R p_1^2 m_2 + C_2 c^1_L c^2_R c^3_R p_1^2 m_2 \nonumber \\&- C_2 c^1_R c^2_L c^3_L p_2^2 m_2 - C_2 c^1_L c^2_R c^3_R p_2^2 m_2 \nonumber \\&+ 2 C_0 c^1_R c^2_L c^3_L m_1^2 m_2 + 2 C_0 c^1_L c^2_R c^3_R m_1^2 m_2\nonumber \\&-4 C_{00} \left( c^1_L c^2_L c^3_L m_1 + c^1_R c^2_R c^3_R m_1\right. \nonumber \\&\left. + c^1_R c^2_L c^3_L m_2 + c^1_L c^2_R c^3_R m_2\right) \nonumber \\&- \left( \left( c^1_L c^2_R c^3_L + c^1_R c^2_L c^3_R\right) \left( 2 B_0 + 2 C_2 p_0^2 \right. \right. \nonumber \\&\left. + C_1 \left( p_0^2 + p_1^2 - p_2^2\right) + 2 C_0 m_1^2\right) \nonumber \\&\left. + 2 C_0 \left( c^1_R c^2_R c^3_L + c^1_L c^2_L c^3_R\right) m_1 m_2\right) m_3\Big ],\end{aligned}$$

A.141 $$\begin{aligned} M^{(a)}_2 =\,&2 i \Big [C_0 \left( c^1_L c^2_L c^3_L + c^1_R c^2_R c^3_R\right) m_1\nonumber \\&+ \left( 2 C_{12} + 3 C_2 + 2 C_{22}\right) \left( c^1_L c^2_L c^3_L + c^1_R c^2_R c^3_R\right) m_1 \nonumber \\&+ \left( 2 C_{12} + C_2 + 2 C_{22}\right) \left( c^1_R c^2_L c^3_L + c^1_L c^2_R c^3_R\right) m_2\nonumber \\&+ C_1 \left( c^1_L c^2_L c^3_L m_1 + c^1_R c^2_R c^3_R m_1- c^1_L c^2_R c^3_L m_3\right. \nonumber \\&\left. - c^1_R c^2_L c^3_R m_3\right) \Big ] \end{aligned}$$

with $$B_i = B_i(p_2^2, m_2^2, m_3^2)$$ and $$C_i = C_i(p_1^2, p_2^2, p_0^2, m_1^2, m_3^2, m_2^2)$$.

(b) *SSS* diagram

A.142 $$\begin{aligned} M^{(b)}_1 =\,&4 i C_{00} c^1 c^2 c^3 ,\end{aligned}$$

A.143 $$\begin{aligned} M^{(b)}_2 =\,&4 i (C_{12} + C_2 + C_{22}) c^1 c^2 c^3, \end{aligned}$$

with $$C_i = C_i(p_1^2, p_2^2, p_0^2, m_1^2, m_3^2, m_2^2)$$.

(c) *UUU* diagram

A.144 $$\begin{aligned} M^{(c)}_1 =\,&i C_{00} c^1 c^2 c^3 ,\end{aligned}$$

A.145 $$\begin{aligned} M^{(c)}_2 =\,&i (C_{12} + C_2 + C_{22}) c^1 c^2 c^3, \end{aligned}$$

with $$C_i = C_i(p_1^2, p_2^2, p_0^2, m_1^2, m_3^2, m_2^2)$$.

(d) *VVV* diagram

A.146 $$\begin{aligned} M^{(d)}_1 =\,&-\frac{i}{2} c^1 c^2 c^3 \Big [4 B_0 + 20 C_{00} -4 r + C_1 p_0^2 \nonumber \\&+ 4 C_2 p_0^2 + 5 C_1 p_1^2 + 2 C_2 p_1^2 \nonumber \\&- (C_1 + 2 C_2) p_2^2 + C_0 (-4 p_0^2 \nonumber \\&+ 6 p_1^2 + 4 (p_2^2 + m_1^2))\Big ] ,\end{aligned}$$

A.147 $$\begin{aligned} M^{(d)}_2 =\,&-i (5 C_0 + C_1 + 10 (C_{12} + C_2 + C_{22})) c^1 c^2 c^3 \end{aligned}$$

with $$B_i = B_i(p_2^2, m_2^2, m_3^2)$$ and $$C_i = C_i(p_1^2, p_2^2, p_0^2, m_1^2, m_3^2, m_2^2)$$.

(e) *SSV* diagram

A.148 $$\begin{aligned} M^{(e)}_1 =\,&i C_0 c^1 c^2 c^3 ,\end{aligned}$$

A.149 $$\begin{aligned} M^{(e)}_2 =\,&0 \end{aligned}$$

with $$C_i=C_i(p_1^2, p_2^2, p_0^2, m_1^2, m_3^2, m_2^2)$$.

(f) *SVS* diagram

A.150 $$\begin{aligned} M^{(f)}_1 =\,&2 i C_{00} c^1 c^2 c^3 ,\end{aligned}$$

A.151 $$\begin{aligned} M^{(f)}_2 =\,&2 i (C_{12} - C_2 + C_{22}) c^1 c^2 c^3 \end{aligned}$$

with $$C_i=C_i(p_1^2, p_2^2, p_0^2, m_1^2, m_3^2, m_2^2)$$.

(g) *VSS* diagram

A.152 $$\begin{aligned} M^{(g)}_1 =\,&2 i C_{00} c^1 c^2 c^3 ,\end{aligned}$$

A.153 $$\begin{aligned} M^{(g)}_2 =\,&2 i (2 C_0 + 2 C_1 + C_{12} + 3 C_2 + C_{22}) c^1 c^2 c^3 \end{aligned}$$

with $$C_i=C_i(p_1^2, p_2^2, p_0^2, m_1^2, m_3^2, m_2^2)$$.

(h) *SVV* diagram

A.154 $$\begin{aligned} M^{(h)}_1 =\,&i c^1 c^2 c^3 \Big [B_0 - C_{00} - (C_1 + C_2) (p^2_0 - p^2_1) \nonumber \\&+ (C_1 - C_2) p_2^2 + C_0 (- p_1^2 + p_2^2 + m_1^2)\Big ] ,\end{aligned}$$

A.155 $$\begin{aligned} M^{(h)}_2 =\,&i (4 C_1 - C_{12} + C_2 - C_{22}) c^1 c^2 c^3 \end{aligned}$$

with $$B_i = B_i(p_2^2, m_2^2, m_3^2)$$ and $$C_i = C_i(p_1^2, p_2^2, p_0^2, m_1^2, m_3^2, m_2^2)$$.

(i) *VSV* diagram

A.156 $$\begin{aligned} M^{(i)}_1 =\,&i c^1 c^2 c^3 \Big [B_0 - C_{00} + C_1 p_0^2 + 3 C_2 p_0^2 + 3 C_1 p_1^2 + C_2 p_1^2 \nonumber \\&- (C_1 + C_2) p_2^2 + C_0 (2 (p_0^2 + p_1^2 - p_2^2) + m_1^2)\Big ] ,\end{aligned}$$

A.157 $$\begin{aligned} M^{(i)}_2 =\,&-i (2 C_0 -2 C_1 + C_{12} + 3 C_2 + C_{22}) c^1 c^2 c^3 \end{aligned}$$

with $$B_i = B_i(p_2^2, m_2^2, m_3^2)$$ and $$C_i = C_i(p_1^2, p_2^2, p_0^2, m_1^2, m_3^2, m_2^2)$$.

(j) *VVS* diagram

A.158 $$\begin{aligned} M^{(j)}_1 =\,&-i C_0(p_1^2, p_2^2, p_0^2, m_1^2, m_3^2, m_2^2) c^1 c^2 c^3 ,\end{aligned}$$

A.159 $$\begin{aligned} M^{(j)}_2 =\,&0. \end{aligned}$$

(k) *SS* diagram

A.160 $$\begin{aligned} M^{(k)}_1 =\,&\frac{i}{2} B_0(p_0^2, m_1^2, m_2^2) c^1 c^2 ,\end{aligned}$$

A.161 $$\begin{aligned} M^{(k)}_2 =\,&0. \end{aligned}$$

(l) *VV* diagram

A.162 $$\begin{aligned} M^{(l)}_1 =\,&i c^1 c^2 (3 B_0(p_0^2, m_1^2, m_2^2) - r) ,\end{aligned}$$

A.163 $$\begin{aligned} M^{(l)}_2 =\,&0. \end{aligned}$$

(m) *SV* diagram

A.164 $$\begin{aligned} M^{(m)}_1 =\,&-i B_0(p_1^2, m_1^2, m_2^2) c^1 c^2 ,\end{aligned}$$

A.165 $$\begin{aligned} M^{(m)}_2 =\,&0. \end{aligned}$$

(n) *SV* diagram

A.166 $$\begin{aligned} M^{(n)}_1 =\,&-i B_0(p_2^2, m_1^2, m_2^2) c^1 c^3 ,\end{aligned}$$

A.167 $$\begin{aligned} M^{(n)}_2 =\,&0. \end{aligned}$$

## Appendix B: Expressions for real corrections

Following the notation introduced in Sect. 2.6, we present here the formulae employed for the calculation of real corrections (massless gauge boson emission) to the six generic decays under consideration. Common to all processes is the calculation of the group-theory factors, which we describe in the first subsection.

### B.1: Group-theory factors

For real three-body decays, we start with a two-body matrix element $\mathcal {M}_0$ where a particle having four-momentum $p_0$ decays to two others with four-momenta $p_1,p_2$, and we can attach a photon in *four* ways: either to the incoming or outgoing legs, or to the vertex itself. This latter case is only possible when the tree-level vertex involves two scalars and a massive gauge boson; in SARAH such vertices are stored in the form

B.1 $$\begin{aligned}&\mathcal {L} \supset -ic_{ija} (S_i \partial _\mu \overline{S}_j - \overline{S}_j \partial _\mu S_i ) V^{a\,\mu } \nonumber \\&\qquad \rightarrow c_{ija} S_i \overline{S}_j V^{a\,\mu } (p_\mu (S_i) - p_\mu (\overline{S}_j)). \end{aligned}$$

If we attach a massless gauge boson $A_\mu ^b$ to the above term then we can have the coupling

B.2 $$\begin{aligned} \mathcal {L}\supset c_{ijab} S_i \overline{S}_j V^{a\,\mu } A_\mu ^b. \end{aligned}$$

We should now understand that the indices for *V*, *A* correspond to the *unbroken* gauge symmetry under which $A_\mu ^b$ transforms. By insisting on gauge invariance up to linear order we find three sets of conditions:

B.3 $$\begin{aligned} 0=\,&T_{ii'}^c (S_i)c_{i'ja} + T_{jj'}^c (\overline{S}_j)c_{ij'a} + T_{a a'}^c (V) c_{ija'} ,\end{aligned}$$

B.4 $$\begin{aligned} =\,&T_{ii'}^c (S_i)c_{i'jab} + T_{jj'}^c (\overline{S}_j)c_{ij'ab} + T_{a a'}^c (V) c_{ija'b} \end{aligned}$$

B.5 $$\begin{aligned}&+ i f^{cbb'} c_{ijab'} ,= c_{ijab} + c_{ij'a} T^c_{j'j} (\overline{S}_j) - c_{i'ja} T^c_{i'i} (S_i). \end{aligned}$$

Summation is only implied over the primed indices. The first two equations arise from requiring simple gauge invariance of the individual vertices, while the third involves the derivatives of the gauge transformation cancelling against the $A_\mu ^a \rightarrow A_\mu ^a + \partial _\mu \Lambda ^a + \cdots$ part. Importantly, Eq. (B.5) completely determines $c_{ijab}$.

If the gauge group is that of the SM, then the quartic coupling can only be relevant for a *W* boson and a photon, for which case the first two equations simply become charge conservation and Eq. (B.5) implies

B.6 $$\begin{aligned} c_{ijW^-A} =&(Q_{S_i} - Q_{\bar{S}_j}) c_{ijW^-} = (2 Q_{S_i} - 1)c_{ijW^-}. \end{aligned}$$

The above logic can also be used to compute the group-theory factors for the Bremsstrahlung decay cross-sections, where a particle with momentum $p_0$ decays to final states with momenta $p_1, p_2$ and a photon/gluon with momentum *k*.

We first compute all of the relevant processes using only the primitive vertices stripped of group-theory factors, and split up the squared amplitude depending on which leg the photon or gluon propagator has been attached. We first make a general definition of the coupling $$c_{ijk} $$ in the same was as we did in Eqs. (B.1) and (B.2) and strip off any Lorentz indices, so for fields (scalars, fermions or vectors) $$\Phi _i$$ in representations of the gauge group $$R_0 \rightarrow R_1, R_2$$ we have the coupling

B.7 $$\begin{aligned} \mathcal {L}\supset c_{ijk} \Phi _i \overline{\Phi }_j \overline{\Phi }_k \times \mathrm {Lorentz\ part}. \end{aligned}$$

Then the matrix element is

B.8 $$\begin{aligned} (\mathcal {M}_{1})_{j_0 j_1 j_2 a} =&g \sum _{j^\prime _1} T^a(R_0)_{j_0^\prime j_0} c_{j^\prime _0 j_1 j_2} \frac{f_0(p_i)}{-2p_0 \cdot k} \nonumber \\&+ g \sum _{j^\prime _1} T^a(R_1)_{j_1 j^\prime _1 } c_{j_0 j^\prime _1 j_2} \frac{f_1(p_i)}{2p_1 \cdot k} \nonumber \\&+ g \sum _{j^\prime _2} T^a(R_2)_{j_2 j^\prime _2 } c_{j_0 j_1 j_2^\prime } \frac{f_2(p_i)}{2p_2 \cdot k}\nonumber \\&+ c_{j_0 j_1 j_2 a} f_3(p_i) , \end{aligned}$$

where the functions $f_i^\mu (p_i)$ may have spinor or other Lorentz indices, and include the wave-function factors, as appropriate to the final states; and the index *i* labels the leg to which the photon/gluon is attached: 0 for incoming, 1, 2 for the outgoing legs and 3 to denote the vertex in the case of scalar to scalar plus gauge boson decays. We have explicitly included the intermediate propagators to show the potential infrared divergences—and also because they allow easy identification of the appropriate diagram.

Then defining $x_0 \equiv - 2p_0 \cdot k, x_1 \equiv 2 p_1 \cdot k, x_2 \equiv 2p_2 \cdot k, x_3 \equiv 1$, a generic squared matrix element can be expressed as

B.9 $$\begin{aligned} |\mathcal {M}_1|^2 \equiv&\sum _{i,j=0}^3 \tilde{C}_{ij} \sum _\mathrm{spins} \frac{f_i f_j^*}{x_i x_j}. \end{aligned}$$

Now all of the group-theory factors are encoded in $\tilde{C}_{ij}$. However, while $\tilde{C}_{ji} = \tilde{C}_{ij}^*$, it is also clear from the hermiticity of the generators that the $\tilde{C}_{ij}$ are real—for example using $d(R_0)$ as the dimension of representation $R_0$

B.10 $$\begin{aligned} (\tilde{C}_{12})^* =&\frac{1}{d(R_0)} \left( \sum _{i,j,k,i',j'} c_{i'jk} T^a (R_0)_{i'i} T^a(R_1)^*_{jj'} c_{ij'k}^* \right) ^* \nonumber \\ =&\frac{1}{d(R_0)} \sum _{i,j,k,i',j'} c_{i'jk}^* T^a (R_0)_{ii'} T^a(R_1)_{j'j} c_{ij'k} \nonumber \\ =&\frac{1}{d(R_0)} \sum _{i,j,k,i',j'} c_{i'j k} T^a (R_0)_{i'i} T^a(R_1)_{jj'} c_{ij'k}^* \!=\! \tilde{C}_{12} \end{aligned}$$

and therefore we can write

B.11 $$\begin{aligned} |\mathcal {M}_1|^2 = \sum _{i \le j}^3 \tilde{C}_{ij} \omega _{ij}, \quad \omega _{ij} \equiv \left\{ \begin{array}{ll} \sum _\mathrm{spins} \frac{f_i f_j^* + f_j f_i^*}{x_i x_j}, &{} i < j \\ \sum _\mathrm{spins} \frac{f_i f_i^*}{x_i x_j}, &{} i = j \end{array}\right. . \end{aligned}$$

Using the gauge invariance of the Lagrangian terms, we obtain for $i<3$

B.12 $$\begin{aligned} 0=&\,\tilde{C}_{0j} - \tilde{C}_{1j} - \tilde{C}_{2j} = \tilde{C}_{i0} - \tilde{C}_{i1} - \tilde{C}_{i2} \nonumber \\ =&\,\tilde{C}_{i3} - \tilde{C}_{i0} - \tilde{C}_{i1} = \tilde{C}_{3i} - \tilde{C}_{0i} - \tilde{C}_{1i}, \end{aligned}$$

where for $S\rightarrow SV$ decays we define the heavy gauge boson as particle 2. These are almost enough to completely determine the colour factor of the amplitude in terms of the two-body decay factor *C*,

B.13 $$\begin{aligned} c_{ijk} c^*_{i'jk} \equiv C \delta _{ii'}, \end{aligned}$$

because we can write

B.14 $$\begin{aligned} \tilde{C}_{ii} =&\, C_2 (R_i) C. \end{aligned}$$

These conditions are sufficient to determine all of the group factors in terms of *C*, the quadratic Casimir $C_2(R_i)$, and one remaining colour factor. However, we can also use the gauge invariance of the Lagrangian term to second order to obtain

B.15 $$\begin{aligned} 0=&\tilde{C}_{00} + \tilde{C}_{11} + \tilde{C}_{22} - 2\tilde{C}_{01} -2\tilde{C}_{02}\nonumber \\&+ 2\tilde{C}_{12} = -\tilde{C}_{00} + \tilde{C}_{11} + \tilde{C}_{22} + 2\tilde{C}_{12}. \end{aligned}$$

Hence all of the group-theory factors are proportional to *C*, and we can therefore define

B.16 $$\begin{aligned} C_{ij} \equiv \frac{\tilde{C}_{ij}}{C}, \end{aligned}$$

giving

B.17 $$\begin{aligned} C_{ii} =\,&C_{2}(R_i), \qquad i<3 ,\nonumber \\ C_{12} =\,&\frac{1}{2} \left( C_2\left( R_0\right) - C_2\left( R_1\right) - C_2\left( R_2\right) \right) ,\nonumber \\ C_{02} =\,&\frac{1}{2} \left( C_2\left( R_0\right) - C_2\left( R_1\right) + C_2\left( R_2\right) \right) ,\nonumber \\ C_{01} =\,&\frac{1}{2} \left( C_2\left( R_0\right) + C_2\left( R_1\right) - C_2\left( R_2\right) \right) ,\nonumber \\ C_{i3} =\,&C_{3i} = C_{i1} + C_{i2}, \qquad i<3, \nonumber \\ C_{33} =\,&\left( C_2 (R_0) + C_2(R_1)\right) C + 2 C_{01} \nonumber \\ =\,&2C_2(R_0) + 2C_2(R_1) - C_2(R_2), \end{aligned}$$

and we can write for a generic process

B.18 $$\begin{aligned} \Gamma \propto \Omega \equiv \sum _{i \le j}^3 C_{ij} \Omega _{ij}. \end{aligned}$$

In the case of a U(1) gauge boson (the photon), we have $C_{ij} = Q_i Q_j$ and we define $Q_3 \equiv Q_0 + Q_1.$ This also follows from the above expressions, for example

B.19 $$\begin{aligned} C_{12} =&\frac{1}{2} ( Q_0^2 - Q_1^2 - Q_2^2) = \frac{1}{2} \left( (Q_1 + Q_2)^2 - Q_1^2 - Q_2^2\right) \nonumber \\ =&Q_1 Q_2. \end{aligned}$$

### B.2: $F\rightarrow FS$

The real corrections for the fermionic decays $F\rightarrow FS$ are given by

B.20 $$\begin{aligned}&\Gamma _{F\rightarrow FS+\gamma /g}=\frac{c_g^2}{(4\pi )^3m_X}C'_\mathrm{S}\nonumber \\&\quad \times \left[ (c_Lc_L^*+c_Rc_R^*)\Omega ^{LL}+(c_Lc_R^*+c_Rc_L^*)\Omega ^{LR}\right] . \end{aligned}$$

The coupling $c_g$ is the electromagnetic coupling *e* or the strong coupling $g_s$ for photon and gluon emission, respectively. $c_L$ and $c_R$ are the left- and right-handed coupling of the tree-level vertex of $F\rightarrow FS$. We identify $F_{\text {in}}=X$, $F_{\text {out}}=Y_1$ and $S=Y_2$ with the indices 0, 1 and 2, respectively. The individual contributions $\Omega _{ij}^{AB}$ are given by

B.21 $$\begin{aligned} \Omega _{00}^{LR} =\,&-8 I_{00} m_X^3 m_{Y_1} ,\end{aligned}$$

B.22 $$\begin{aligned} \Omega _{01}^{LR} =\,&-8 I_0 m_X m_{Y_1} - 8 I_1 m_X m_{Y_1} \nonumber \\&+ I_{01} \left( -8 m_X^3 m_{Y_1} - 8 m_X m_{Y_1}^3 + 8 m_X m_{Y_1} m_{Y_2}^2\right) ,\end{aligned}$$

B.23 $$\begin{aligned} \Omega _{02}^{LR} =\,&8 I_0 m_X m_{Y_1} + I_{02} \left( 8 m_X^3 m_{Y_1} \right. \nonumber \\&- \left. 8 m_X m_{Y_1}^3 + 8 m_X m_{Y_1} m_{Y_2}^2\right) ,\end{aligned}$$

B.24 $$\begin{aligned} \Omega _{11}^{LR} =\,&-8 I_{11} m_X m_{Y_1}^3 ,\end{aligned}$$

B.25 $$\begin{aligned} \Omega _{12}^{LR} =\,&-8 I_1 m_X m_{Y_1} + I_{12} \left( 8 m_X^3 m_{Y_1} \right. \nonumber \\&-\left. 8 m_X m_{Y_1}^3 - 8 m_X m_{Y_1} m_{Y_2}^2\right) ,\end{aligned}$$

B.26 $$\begin{aligned} \Omega _{22}^{LR} =\,&-8 I_{2} m_X m_{Y_1} - 8 I_{22} m_X m_{Y_1} m_{Y_2}^2 ,\end{aligned}$$

B.27 $$\begin{aligned} \Omega _{00}^{LL} =\,&2 I + I_0 \left( -2 m_X^2 + 2 m_{Y_1}^2 - 2 m_{Y_2}^2\right) \nonumber \\&+ I_{00} \left( -4 m_X^4 - 4 m_X^2 m_{Y_1}^2 + 4 m_X^2 m_{Y_2}^2\right) ,\end{aligned}$$

B.28 $$\begin{aligned} \Omega _{01}^{LL} =\,&-8 I - 2 I_0^1 - 2 I_1^0 + I_0 \left( -2 m_X^2 - 6 m_{Y_1}^2+ 6 m_{Y_2}^2\right) \nonumber \\&+ I_1 \left( -6 m_X^2 - 2 m_{Y_1}^2 + 6 m_{Y_2}^2\right) \nonumber \\&+ I_{01} \left( -4 m_X^4 - 8 m_X^2 m_{Y_1}^2 - 4 m_{Y_1}^4 + 8 m_X^2 m_{Y_2}^2 \right. \nonumber \\&+\left. 8 m_{Y_1}^2 m_{Y_2}^2 - 4 m_{Y_2}^4\right) ,\end{aligned}$$

B.29 $$\begin{aligned} \Omega _{02}^{LL} =\,&4 I - 2 I_0^2 + I_0 \left( 2 m_X^2 + 6 m_{Y_1}^2 - 6 m_{Y_2}^2\right) + 4 I_{2} m_{Y_2}^2 \nonumber \\&+ I_{02} \left( 4 m_X^4 - 4 m_{Y_1}^4 + 8 m_{Y_1}^2 m_{Y_2}^2 - 4 m_{Y_2}^4\right) ,\end{aligned}$$

B.30 $$\begin{aligned} \Omega _{11}^{LL} =\,&2 I + I_1 \left( 2 m_X^2 - 2 m_{Y_1}^2 - 2 m_{Y_2}^2\right) \nonumber \\&+ I_{11} \left( -4 m_X^2 m_{Y_1}^2 - 4 m_{Y_1}^4 + 4 m_{Y_1}^2 m_{Y_2}^2\right) ,\end{aligned}$$

B.31 $$\begin{aligned} \Omega _{12}^{LL} =\,&-\,4 I \!+ \!2 I_1^2 \!-\! 4 I_{2} m_{Y_2}^2 \!+\! I_1 \left( -6 m_X^2 - 2 m_{Y_1}^2 + 6 m_{Y_2}^2\right) \nonumber \\&+ I_{12} \left( 4 m_X^4 - 4 m_{Y_1}^4 - 8 m_X^2 m_{Y_2}^2 + 4 m_{Y_2}^4\right) ,\end{aligned}$$

B.32 $$\begin{aligned} \Omega _{22}^{LL} = \,&4 I + I_{2} \left( -4 m_X^2 - 4 m_{Y_1}^2 + 8 m_{Y_2}^2\right) \nonumber \\&+ I_{22} \left( -4 m_X^2 m_{Y_2}^2 - 4 m_{Y_1}^2 m_{Y_2}^2 + 4 m_{Y_2}^4\right) . \end{aligned}$$

We also implemented real corrections for which $Y_1$ is taken to be massless, if $Y_1$ is charge and colour neutral.

### B.3: $S\rightarrow FF$

The real corrections for the scalar decays $S\rightarrow FF$ are given by

B.33 $$\begin{aligned} \Gamma _{S\rightarrow FF+\gamma /g}&=\frac{c_g^2}{(4\pi )^3m_X}C'_\mathrm{S}\left[ \left( c_Lc_L^*+c_Rc_R^*\right) \Omega ^{LL}\right. \nonumber \\&+\left. \left( c_Lc_R^*+c_Rc_L^*\right) \Omega ^{LR}\right] . \end{aligned}$$

We follow the notation introduced in the previous subsection. However, we now identify $S=X$, $F=Y_1$ and $F=Y_2$ with the indices 0, 1 and 2, respectively. The individual contributions $\Omega _{ij}^{AB}$ are given by

B.34 $$\begin{aligned} \Omega _{00}^{LR} =\,&16 I_{00} m_X^2 m_{Y_1} m_{Y_2} ,\end{aligned}$$

B.35 $$\begin{aligned} \Omega _{01}^{LR} =\,&-16 I_0 m_{Y_1} m_{Y_2} - 16 I_1 m_{Y_1} m_{Y_2} \nonumber \\&+ I_{01} (-16 m_X^2 m_{Y_1} m_{Y_2} - 16 m_{Y_1}^3 m_{Y_2} + 16 m_{Y_1} m_{Y_2}^3) ,\end{aligned}$$

B.36 $$\begin{aligned} \Omega _{02}^{LR} =\,&16 I_0 m_{Y_1} m_{Y_2} + I_{02} (16 m_X^2 m_{Y_1} m_{Y_2} \nonumber \\&- 16 m_{Y_1}^3 m_{Y_2} + 16 m_{Y_1} m_{Y_2}^3) ,\end{aligned}$$

B.37 $$\begin{aligned} \Omega _{11}^{LR} =\,&16 I_{11} m_{Y_1}^3 m_{Y_2} ,\end{aligned}$$

B.38 $$\begin{aligned} \Omega _{12}^{LR} =\,&16 I_1 m_{Y_1} m_{Y_2} + I_{12} (-16 m_X^2 m_{Y_1} m_{Y_2} \nonumber \\&+ 16 m_{Y_1}^3 m_{Y_2} + 16 m_{Y_1} m_{Y_2}^3) ,\end{aligned}$$

B.39 $$\begin{aligned} \Omega _{22}^{LR} =\,&16 I_{2} m_{Y_1} m_{Y_2} + 16 I_{22} m_{Y_1} m_{Y_2}^3 ,\end{aligned}$$

B.40 $$\begin{aligned} \Omega _{00}^{LL} =\,&-8 I_0 m_X^2 + I_{00} (-8 m_X^4 + 8 m_X^2 m_{Y_1}^2 \nonumber \\&+ 8 m_X^2 m_{Y_2}^2) ,\end{aligned}$$

B.41 $$\begin{aligned} \Omega _{01}^{LL} =\,&4 I + 4 I_1^0 + I_1 (12 m_X^2 - 4 m_{Y_1}^2 \nonumber \\&- 12 m_{Y_2}^2) + I_0 (-8 m_{Y_1}^2 - 8 m_{Y_2}^2)\nonumber \\&+ I_{01} (8 m_X^4 - 8 m_{Y_1}^4 - 16 m_X^2 m_{Y_2}^2 + 8 m_{Y_2}^4) ,\end{aligned}$$

B.42 $$\begin{aligned} \Omega _{02}^{LL} =\,&-4 I - 4 I_2^0 + I_{2} (-12 m_X^2 + 12 m_{Y_1}^2 + 4 m_{Y_2}^2) \nonumber \\&+ I_0 (8 m_{Y_1}^2 + 8 m_{Y_2}^2)\nonumber \\&+ I_{02} (-8 m_X^4 + 16 m_X^2 m_{Y_1}^2 - 8 m_{Y_1}^4 + 8 m_{Y_2}^4) ,\end{aligned}$$

B.43 $$\begin{aligned} \Omega _{11}^{LL} =\,&I_1 (4 m_X^2 + 4 m_{Y_1}^2 - 4 m_{Y_2}^2) \nonumber \\&+ I_{11} (-8 m_X^2 m_{Y_1}^2 + 8 m_{Y_1}^4 + 8 m_{Y_1}^2 m_{Y_2}^2) ,\end{aligned}$$

B.44 $$\begin{aligned} \Omega _{12}^{LL} =\,&8 I + 4 I_1^2 + 4 I_2^1 + I_{2} (-12 m_X^2 + 12 m_{Y_1}^2 \nonumber \\&+ 4 m_{Y_2}^2) + I_1 (-12 m_X^2 + 4 m_{Y_1}^2\nonumber \\&+ 12 m_{Y_2}^2) + I_{12} (8 m_X^4 - 16 m_X^2 m_{Y_1}^2 + 8 m_{Y_1}^4 \nonumber \\&- 16 m_X^2 m_{Y_2}^2 + 16 m_{Y_1}^2 m_{Y_2}^2 + 8 m_{Y_2}^4) ,\end{aligned}$$

B.45 $$\begin{aligned} \Omega _{22}^{LL} =\,&I_{2} (4 m_X^2 - 4 m_{Y_1}^2 + 4 m_{Y_2}^2) \nonumber \\&+ I_{22} (-8 m_X^2 m_{Y_2}^2 + 8 m_{Y_1}^2 m_{Y_2}^2 + 8 m_{Y_2}^4). \end{aligned}$$

We also implemented real corrections where one of the final-state fermions can be massless. Again this fermion has to be charge and colour neutral.

### B.4: $F\rightarrow FV$

The real corrections for the fermionic decays $F\rightarrow FV$ are given by

B.46 $$\begin{aligned}&\Gamma _{F\rightarrow FV+\gamma /g}=\frac{c_g^2}{(4\pi )^3m_{Y_2}^2m_X}C'_\mathrm{S}\nonumber \\&\quad \times \left[ \left( c_Lc_L^*+c_Rc_R^*\right) \Omega ^{LL}+\left( c_Lc_R^*+c_Rc_L^*\right) \Omega ^{LR}\right] . \end{aligned}$$

The coupling $c_g$ is the electromagnetic coupling *e* or the strong coupling $g_s$ for photon and gluon emission, respectively. $c_L$ and $c_R$ are the left- and right-handed coupling of the tree-level vertex of $F\rightarrow FV$. The final-state gauge boson $Y_2$ is fixed to one of the two heavy gauge bosons, *Z* or *W*. Clearly only the photon couples to the *W* boson, such that we subsequently present results independently for $F\rightarrow FW+\gamma$ on the one side and $F\rightarrow FZ+\gamma /g$ and $F\rightarrow FW+g$ on the other side. For gluon emission $C_{00} = C_{11} = C_{01} = C_2(R_F), C_{i2} =0$; for photon emission $C_{ij}=Q_iQ_j$, where we again identify $F_{\text {in}}=X$ and $F_{\text {out}}=Y_1$ with indices 0 and 1, respectively.

For $F\rightarrow FW+\gamma$ the individual contributions are given by

B.47 $$\begin{aligned} \nonumber \Omega _{00}^{LR} =\,&-4 I m_X m_{Y_1} - 4 I_0^2 m_X m_{Y_1} + 24 I_0 m_X m_{Y_1} m_{Y_2}^2\\ \nonumber&+ 24 I_{2} m_X m_{Y_1} m_{Y_2}^2 + 24 I_{00} m_X^3 m_{Y_1} m_{Y_2}^2 \\&+ 24 I_{02} m_X^3 m_{Y_1} m_{Y_2}^2\nonumber \\&- 24 I_{02} m_X m_{Y_1}^3 m_{Y_2}^2 + 24 I_{02} m_X m_{Y_1} m_{Y_2}^4 \nonumber \\&+ 24 I_{22} m_X m_{Y_1} m_{Y_2}^4 ,\end{aligned}$$

B.48 $$\begin{aligned} \Omega _{01}^{LR} =\,&12 I m_X m_{Y_1} + 4 I_0^1 m_X m_{Y_1} + 4 I_0^2 m_X m_{Y_1}\nonumber \\&- 48 I_{2} m_X m_{Y_1} m_{Y_2}^2\nonumber \\ \nonumber&- 48 I_{02} m_X^3 m_{Y_1} m_{Y_2}^2 + 48 I_{01} m_X m_{Y_1}^3 m_{Y_2}^2 \\&+ 48 I_{02} m_X m_{Y_1}^3 m_{Y_2}^2- 48 I_{22} m_X m_{Y_1} m_{Y_2}^4 ,\end{aligned}$$

B.49 $$\begin{aligned} \Omega _{11}^{LR} =\,&4 I_1^0 m_X m_{Y_1} + 24 I_1 m_X m_{Y_1} m_{Y_2}^2 \nonumber \\&+ 24 I_{2} m_X m_{Y_1} m_{Y_2}^2\nonumber \\&+ 24 I_{01} m_X^3 m_{Y_1} m_{Y_2}^2 + 24 I_{02} m_X^3 m_{Y_1} m_{Y_2}^2 \nonumber \\&- 24 I_{01} m_X m_{Y_1}^3 m_{Y_2}^2\nonumber \\ \nonumber&- 24 I_{02} m_X m_{Y_1}^3 m_{Y_2}^2 + 24 I_{11} m_X m_{Y_1}^3 m_{Y_2}^2 \\&- 24 I_{01} m_X m_{Y_1} m_{Y_2}^4\nonumber \\&- 24 I_{02} m_X m_{Y_1} m_{Y_2}^4 + 24 I_{22} m_X m_{Y_1} m_{Y_2}^4 ,\end{aligned}$$

B.50 $$\begin{aligned} \nonumber \Omega _{00}^{LL} =\,&2 I m_X^2 + 2 I_0^2 m_X^2 - 4 I_0 m_X^4 - 4 I_{2} m_X^4 - 4 I_{00} m_X^6 \\&- 4 I_{02} m_X^6 + 2 I m_{Y_1}^2 + 2 I_0^2 m_{Y_1}^2\nonumber \\ \nonumber&+ 8 I_0 m_X^2 m_{Y_1}^2 + 8 I_{2} m_X^2 m_{Y_1}^2 + 8 I_{00} m_X^4 m_{Y_1}^2 \\&+ 12 I_{02} m_X^4 m_{Y_1}^2 - 4 I_0 m_{Y_1}^4 - 4 I_{2} m_{Y_1}^4\nonumber \\ \nonumber&- 4 I_{00} m_X^2 m_{Y_1}^4 - 12 I_{02} m_X^2 m_{Y_1}^4 + 4 I_{02} m_{Y_1}^6 + 4 I m_{Y_2}^2 \\&+ 4 I_0^2 m_{Y_2}^2 - 8 I_2^1 m_{Y_2}^2\nonumber \\ \nonumber&- 8 I_{22}^{01} m_{Y_2}^2 - 4 I_0 m_X^2 m_{Y_2}^2 - 4 I_{2} m_X^2 m_{Y_2}^2 \\&- 4 I_{00} m_X^4 m_{Y_2}^2 - 8 I_{02} m_X^4 m_{Y_2}^2\nonumber \\ \nonumber&- 4 I_{22} m_X^4 m_{Y_2}^2 - 4 I_0 m_{Y_1}^2 m_{Y_2}^2 - 4 I_{2} m_{Y_1}^2 m_{Y_2}^2\\&- 4 I_{00} m_X^2 m_{Y_1}^2 m_{Y_2}^2\nonumber \\ \nonumber&+ 8 I_{02} m_X^2 m_{Y_1}^2 m_{Y_2}^2 + 8 I_{22} m_X^2 m_{Y_1}^2 m_{Y_2}^2 \\&- 4 I_{22} m_{Y_1}^4 m_{Y_2}^2 + 8 I_0 m_{Y_2}^4\nonumber \\ \nonumber&+ 8 I_{2} m_{Y_2}^4 + 8 I_{00} m_X^2 m_{Y_2}^4 + 4 I_{02} m_X^2 m_{Y_2}^4 \\&- 4 I_{22} m_X^2 m_{Y_2}^4 - 12 I_{02} m_{Y_1}^2 m_{Y_2}^4\nonumber \\&- 4 I_{22} m_{Y_1}^2 m_{Y_2}^4 + 8 I_{02} m_{Y_2}^6 + 8 I_{22} m_{Y_2}^6 ,\end{aligned}$$

B.51 $$\begin{aligned} \nonumber \Omega _{01}^{LL} =\,&-6 I m_X^2 - 2 I_0^1 m_X^2 - 2 I_0^2 m_X^2 + 8 I_{2} m_X^4\\&+ 8 I_{02} m_X^6 - 6 I m_{Y_1}^2 - 2 I_0^1 m_{Y_1}^2\nonumber \\ \nonumber&- 2 I_0^2 m_{Y_1}^2 - 16 I_{2} m_X^2 m_{Y_1}^2 - 8 I_{01} m_X^4 m_{Y_1}^2\\&- 24 I_{02} m_X^4 m_{Y_1}^2 + 8 I_{2} m_{Y_1}^4\nonumber \\ \nonumber&+ 16 I_{01} m_X^2 m_{Y_1}^4 + 24 I_{02} m_X^2 m_{Y_1}^4 - 8 I_{01} m_{Y_1}^6 \\&- 8 I_{02} m_{Y_1}^6 + 4 I m_{Y_2}^2 - 4 I_0^1 m_{Y_2}^2\nonumber \\ \nonumber&- 4 I_0^2 m_{Y_2}^2 + 16 I_2^0 m_{Y_2}^2 + 16 I_2^1 m_{Y_2}^2\\&+ 16 I_{22}^{01} m_{Y_2}^2 + 8 I_{2} m_X^2 m_{Y_2}^2\nonumber \\ \nonumber&+ 8 I_{02} m_X^4 m_{Y_2}^2 + 8 I_{22} m_X^4 m_{Y_2}^2 \\&+ 8 I_{2} m_{Y_1}^2 m_{Y_2}^2 - 8 I_{01} m_X^2 m_{Y_1}^2 m_{Y_2}^2\nonumber \\ \nonumber&- 16 I_{22} m_X^2 m_{Y_1}^2 m_{Y_2}^2 - 8 I_{01} m_{Y_1}^4 m_{Y_2}^2 \\&- 8 I_{02} m_{Y_1}^4 m_{Y_2}^2 + 8 I_{22} m_{Y_1}^4 m_{Y_2}^2\nonumber \\ \nonumber&- 16 I_{2} m_{Y_2}^4 - 16 I_{02} m_X^2 m_{Y_2}^4 \\&+ 8 I_{22} m_X^2 m_{Y_2}^4 + 16 I_{01} m_{Y_1}^2 m_{Y_2}^4\nonumber \\&+ 16 I_{02} m_{Y_1}^2 m_{Y_2}^4 + 8 I_{22} m_{Y_1}^2 m_{Y_2}^4 - 16 I_{22} m_{Y_2}^6 ,\end{aligned}$$

B.52 $$\begin{aligned} \nonumber \Omega _{11}^{LL} =\,&-2 I_1^0 m_X^2 - 4 I_1 m_X^4 - 4 I_{2} m_X^4 \\&- 4 I_{01} m_X^6 - 4 I_{02} m_X^6 - 2 I_1^0 m_{Y_1}^2\nonumber \\ \nonumber&+ 8 I_1 m_X^2 m_{Y_1}^2 + 8 I_{2} m_X^2 m_{Y_1}^2 + 12 I_{01} m_X^4 m_{Y_1}^2\\&+ 12 I_{02} m_X^4 m_{Y_1}^2 - 4 I_{11} m_X^4 m_{Y_1}^2\nonumber \\ \nonumber&- 4 I_1 m_{Y_1}^4 - 4 I_{2} m_{Y_1}^4 - 12 I_{01} m_X^2 m_{Y_1}^4 \\&- 12 I_{02} m_X^2 m_{Y_1}^4 + 8 I_{11} m_X^2 m_{Y_1}^4\nonumber \\ \nonumber&+ 4 I_{01} m_{Y_1}^6 + 4 I_{02} m_{Y_1}^6 - 4 I_{11} m_{Y_1}^6 \\&- 8 I m_{Y_2}^2 - 4 I_1^0 m_{Y_2}^2 - 16 I_2^0 m_{Y_2}^2\nonumber \\ \nonumber&- 8 I_2^1 m_{Y_2}^2 - 8 I_{22}^{01} m_{Y_2}^2 - 4 I_1 m_X^2 m_{Y_2}^2 \\&- 4 I_{2} m_X^2 m_{Y_2}^2 - 4 I_{22} m_X^4 m_{Y_2}^2\nonumber \\ \nonumber&- 4 I_1 m_{Y_1}^2 m_{Y_2}^2 - 4 I_{2} m_{Y_1}^2 m_{Y_2}^2 \\&- 8 I_{01} m_X^2 m_{Y_1}^2 m_{Y_2}^2 - 8 I_{02} m_X^2 m_{Y_1}^2 m_{Y_2}^2\nonumber \\ \nonumber&- 4 I_{11} m_X^2 m_{Y_1}^2 m_{Y_2}^2 + 8 I_{22} m_X^2 m_{Y_1}^2 m_{Y_2}^2 \\&+ 8 I_{01} m_{Y_1}^4 m_{Y_2}^2 + 8 I_{02} m_{Y_1}^4 m_{Y_2}^2\nonumber \\ \nonumber&- 4 I_{11} m_{Y_1}^4 m_{Y_2}^2 - 4 I_{22} m_{Y_1}^4 m_{Y_2}^2 \\&+ 8 I_1 m_{Y_2}^4 + 8 I_{2} m_{Y_2}^4 + 12 I_{01} m_X^2 m_{Y_2}^4\nonumber \\ \nonumber&+ 12 I_{02} m_X^2 m_{Y_2}^4 - 4 I_{22} m_X^2 m_{Y_2}^4 \\&- 4 I_{01} m_{Y_1}^2 m_{Y_2}^4 - 4 I_{02} m_{Y_1}^2 m_{Y_2}^4\nonumber \\&+ 8 I_{11} m_{Y_1}^2 m_{Y_2}^4 - 4 I_{22} m_{Y_1}^2 m_{Y_2}^4 \nonumber \\&- 8 I_{01} m_{Y_2}^6 - 8 I_{02} m_{Y_2}^6 + 8 I_{22} m_{Y_2}^6. \end{aligned}$$

For $F\rightarrow FZ+\gamma /g$ and $F\rightarrow FW+g$ the individual contributions are given by

B.53 $$\begin{aligned} \Omega _{00}^{LR} =\,&24 I_{00} m_X^3 m_{Y_1} m_{Y_2}^2 ,\end{aligned}$$

B.54 $$\begin{aligned} \nonumber \Omega _{01}^{LR} =\,&8 I m_X m_{Y_1} + 4 I_0^1 m_X m_{Y_1} + 4 I_1^0 m_X m_{Y_1} \\&+ 24 I_0 m_X m_{Y_1} m_{Y_2}^2 + 24 I_1 m_X m_{Y_1} m_{Y_2}^2\nonumber \\&+ 24 I_{01} m_X^3 m_{Y_1} m_{Y_2}^2 + 24 I_{01} m_X m_{Y_1}^3 m_{Y_2}^2 \nonumber \\&- 24 I_{01} m_X m_{Y_1} m_{Y_2}^4 ,\end{aligned}$$

B.55 $$\begin{aligned} \Omega _{11}^{LR} =\,&24 I_{11} m_X m_{Y_1}^3 m_{Y_2}^2 ,\end{aligned}$$

B.56 $$\begin{aligned} \nonumber \Omega _{00}^{LL} =\,&-2 I m_X^2 - 6 I_0 m_X^4 - 4 I_{00} m_X^6 - 2 I m_{Y_1}^2 \\&+ 4 I_0 m_X^2 m_{Y_1}^2 + 8 I_{00} m_X^4 m_{Y_1}^2\nonumber \\ \nonumber&+ 2 I_0 m_{Y_1}^4 - 4 I_{00} m_X^2 m_{Y_1}^4 + 4 I m_{Y_2}^2 \\&- 2 I_0 m_X^2 m_{Y_2}^2 - 4 I_{00} m_X^4 m_{Y_2}^2\nonumber \\&+ 2 I_0 m_{Y_1}^2 m_{Y_2}^2 - 4 I_{00} m_X^2 m_{Y_1}^2 m_{Y_2}^2 \nonumber \\&- 4 I_0 m_{Y_2}^4 + 8 I_{00} m_X^2 m_{Y_2}^4 ,\end{aligned}$$

B.57 $$\begin{aligned} \nonumber \Omega _{01}^{LL} =\,&-2 I_0^1 m_X^2 - 2 I_1^0 m_X^2 + 2 I_0 m_X^4 - 6 I_1 m_X^4\\&- 4 I_{01} m_X^6 - 2 I_0^1 m_{Y_1}^2 - 2 I_1^0 m_{Y_1}^2\nonumber \\ \nonumber&+ 4 I_0 m_X^2 m_{Y_1}^2 + 4 I_1 m_X^2 m_{Y_1}^2 + 4 I_{01} m_X^4 m_{Y_1}^2\\&- 6 I_0 m_{Y_1}^4 + 2 I_1 m_{Y_1}^4 + 4 I_{01} m_X^2 m_{Y_1}^4\nonumber \\ \nonumber&- 4 I_{01} m_{Y_1}^6 - 8 I m_{Y_2}^2 - 4 I_0^1 m_{Y_2}^2 - 4 I_1^0 m_{Y_2}^2 \\&- 2 I_0 m_X^2 m_{Y_2}^2 - 6 I_1 m_X^2 m_{Y_2}^2\nonumber \\ \nonumber&- 6 I_0 m_{Y_1}^2 m_{Y_2}^2 - 2 I_1 m_{Y_1}^2 m_{Y_2}^2 - 16 I_{01} m_X^2 m_{Y_1}^2 m_{Y_2}^2 \\&+ 12 I_0 m_{Y_2}^4 + 12 I_1 m_{Y_2}^4\nonumber \\&+ 12 I_{01} m_X^2 m_{Y_2}^4 + 12 I_{01} m_{Y_1}^2 m_{Y_2}^4 - 8 I_{01} m_{Y_2}^6 ,\end{aligned}$$

B.58 $$\begin{aligned} \nonumber \Omega _{11}^{LL} =\,&-2 I m_X^2 + 2 I_1 m_X^4 - 2 I m_{Y_1}^2 + 4 I_1 m_X^2 m_{Y_1}^2 \\&- 4 I_{11} m_X^4 m_{Y_1}^2 - 6 I_1 m_{Y_1}^4\nonumber \\ \nonumber&+ 8 I_{11} m_X^2 m_{Y_1}^4 - 4 I_{11} m_{Y_1}^6 + 4 I m_{Y_2}^2 \\&+ 2 I_1 m_X^2 m_{Y_2}^2 - 2 I_1 m_{Y_1}^2 m_{Y_2}^2\nonumber \\&- 4 I_{11} m_X^2 m_{Y_1}^2 m_{Y_2}^2 - 4 I_{11} m_{Y_1}^4 m_{Y_2}^2 \nonumber \\&- 4 I_1 m_{Y_2}^4 + 8 I_{11} m_{Y_1}^2 m_{Y_2}^4. \end{aligned}$$

### B.5: $S\rightarrow VV$

The real corrections for the decays $S\rightarrow VV$ are given by

B.59 $$\begin{aligned} \Gamma _{S\rightarrow VV+\gamma }=\frac{e^2}{(4\pi )^3m_{Y_1}^2m_{Y_2}^2m_X}C'_Scc^*\Omega . \end{aligned}$$

The coupling *c* is the tree-level coupling of the vertex of $S\rightarrow VV$. Given that both final-state particles *V* are heavy gauge bosons only photon emission is of relevance for this process. We identify $S=X$, $V=Y_1$ and $V=Y_2$ with the indices 0, 1 and 2, respectively. The individual contributions $\Omega _{ij}$ are given by

B.60 $$\begin{aligned} \nonumber \Omega _{00} =\,&-6 I m_X^2 - 6 I_0 m_X^4 - 2 I_{00} m_X^6 + 4 I m_{Y_1}^2 \\&+ 8 I_0 m_X^2 m_{Y_1}^2 + 4 I_{00} m_X^4 m_{Y_1}^2 - 2 I_0 m_{Y_1}^4\nonumber \\ \nonumber&- 2 I_{00} m_X^2 m_{Y_1}^4 + 4 I m_{Y_2}^2 + 8 I_0 m_X^2 m_{Y_2}^2 \\&+ 4 I_{00} m_X^4 m_{Y_2}^2 - 20 I_0 m_{Y_1}^2 m_{Y_2}^2\nonumber \\&- 20 I_{00} m_X^2 m_{Y_1}^2 m_{Y_2}^2 - 2 I_0 m_{Y_2}^4 - 2 I_{00} m_X^2 m_{Y_2}^4 ,\end{aligned}$$

B.61 $$\begin{aligned} \nonumber \Omega _{01} =\,&8 I m_X^2 - 2 I_1^0 m_X^2 + 4 I_0 m_X^4 - 4 I_1 m_X^4 - 2 I_{01} m_X^6 \\&- 4 I m_{Y_1}^2 + 2 I_1^0 m_{Y_1}^2 - 4 I_0 m_X^2 m_{Y_1}^2\nonumber \\ \nonumber&+ 4 I_1 m_X^2 m_{Y_1}^2 + 2 I_{01} m_X^4 m_{Y_1}^2 + 2 I_{01} m_X^2 m_{Y_1}^4 \\&- 2 I_{01} m_{Y_1}^6 - 4 I m_{Y_2}^2 + 2 I_1^0 m_{Y_2}^2\nonumber \\ \nonumber&- 4 I_0 m_X^2 m_{Y_2}^2 + 8 I_1 m_X^2 m_{Y_2}^2 + 6 I_{01} m_X^4 m_{Y_2}^2\\&- 20 I_1 m_{Y_1}^2 m_{Y_2}^2 - 20 I_{01} m_X^2 m_{Y_1}^2 m_{Y_2}^2\nonumber \\&- 18 I_{01} m_{Y_1}^4 m_{Y_2}^2 - 4 I_1 m_{Y_2}^4 - 6 I_{01} m_X^2 m_{Y_2}^4\nonumber \\&+ 18 I_{01} m_{Y_1}^2 m_{Y_2}^4 + 2 I_{01} m_{Y_2}^6 ,\end{aligned}$$

B.62 $$\begin{aligned} \nonumber \Omega _{02} =\,&8 I m_X^2 - 2 I_2^0 m_X^2 + 4 I_0 m_X^4 - 4 I_{2} m_X^4 - 2 I_{02} m_X^6 \\&- 4 I m_{Y_1}^2 + 2 I_2^0 m_{Y_1}^2 - 4 I_0 m_X^2 m_{Y_1}^2\nonumber \\ \nonumber&+ 8 I_{2} m_X^2 m_{Y_1}^2 + 6 I_{02} m_X^4 m_{Y_1}^2 - 4 I_{2} m_{Y_1}^4 \\&- 6 I_{02} m_X^2 m_{Y_1}^4 + 2 I_{02} m_{Y_1}^6 - 4 I m_{Y_2}^2\nonumber \\ \nonumber&+ 2 I_2^0 m_{Y_2}^2 - 4 I_0 m_X^2 m_{Y_2}^2 + 4 I_{2} m_X^2 m_{Y_2}^2 \\&+ 2 I_{02} m_X^4 m_{Y_2}^2 - 20 I_{2} m_{Y_1}^2 m_{Y_2}^2\nonumber \\&- 20 I_{02} m_X^2 m_{Y_1}^2 m_{Y_2}^2 + 18 I_{02} m_{Y_1}^4 m_{Y_2}^2 \nonumber \\&+ 2 I_{02} m_X^2 m_{Y_2}^4 - 18 I_{02} m_{Y_1}^2 m_{Y_2}^4 - 2 I_{02} m_{Y_2}^6 ,\end{aligned}$$

B.63 $$\begin{aligned} \nonumber \Omega _{11} =\,&-2 I m_X^2 + 2 I_1^0 m_X^2 + 2 I_1 m_X^4 + 4 I m_{Y_1}^2 + 6 I_1^0 m_{Y_1}^2 \\&+ 4 I_{11}^{00} m_{Y_1}^2 - 2 I_{11} m_X^4 m_{Y_1}^2\nonumber \\ \nonumber&- 2 I_1 m_{Y_1}^4 + 4 I_{11} m_X^2 m_{Y_1}^4 - 2 I_{11} m_{Y_1}^6\\&+ 4 I m_{Y_2}^2 - 2 I_1^0 m_{Y_2}^2 - 4 I_1 m_X^2 m_{Y_2}^2\nonumber \\&+ 4 I_{11} m_X^2 m_{Y_1}^2 m_{Y_2}^2 - 20 I_{11} m_{Y_1}^4 m_{Y_2}^2 \nonumber \\&+ 2 I_1 m_{Y_2}^4 - 2 I_{11} m_{Y_1}^2 m_{Y_2}^4 ,\end{aligned}$$

B.64 $$\begin{aligned} \nonumber \Omega _{12} =\,&-8 I m_X^2 - 2 I_1^2 m_X^2 - 2 I_2^1 m_X^2 + 4 I_1 m_X^4 \\&+ 4 I_{2} m_X^4 - 2 I_{12} m_X^6 + 4 I m_{Y_1}^2 - 6 I_1^2 m_{Y_1}^2\nonumber \\ \nonumber&+ 2 I_2^1 m_{Y_1}^2 - 4 I_1 m_X^2 m_{Y_1}^2 - 8 I_{2} m_X^2 m_{Y_1}^2 \\&+ 6 I_{12} m_X^4 m_{Y_1}^2 + 4 I_{2} m_{Y_1}^4 - 6 I_{12} m_X^2 m_{Y_1}^4\nonumber \\ \nonumber&+ 2 I_{12} m_{Y_1}^6 + 4 I m_{Y_2}^2 + 2 I_1^2 m_{Y_2}^2 - 6 I_2^1 m_{Y_2}^2 \\&- 8 I_1 m_X^2 m_{Y_2}^2 - 4 I_{2} m_X^2 m_{Y_2}^2\nonumber \\ \nonumber&+ 6 I_{12} m_X^4 m_{Y_2}^2 + 20 I_1 m_{Y_1}^2 m_{Y_2}^2\\&+ 20 I_{2} m_{Y_1}^2 m_{Y_2}^2 - 28 I_{12} m_X^2 m_{Y_1}^2 m_{Y_2}^2\nonumber \\&+ 22 I_{12} m_{Y_1}^4 m_{Y_2}^2 + 4 I_1 m_{Y_2}^4 - 6 I_{12} m_X^2 m_{Y_2}^4 \nonumber \\&+ 22 I_{12} m_{Y_1}^2 m_{Y_2}^4 + 2 I_{12} m_{Y_2}^6 ,\end{aligned}$$

B.65 $$\begin{aligned} \nonumber \Omega _{22} =\,&-4 I m_X^2 - 2 I_2^1 m_X^2 + 2 I_{2} m_X^4 + 6 I m_{Y_1}^2 \\&+ 2 I_2^1 m_{Y_1}^2 - 4 I_{2} m_X^2 m_{Y_1}^2 + 2 I_{2} m_{Y_1}^4\nonumber \\ \nonumber&+ 2 I m_{Y_2}^2 + 2 I_2^1 m_{Y_2}^2 + 4 I_{22}^{11} m_{Y_2}^2 \\&- 2 I_{22} m_X^4 m_{Y_2}^2 + 4 I_{22} m_X^2 m_{Y_1}^2 m_{Y_2}^2\nonumber \\&- 2 I_{22} m_{Y_1}^4 m_{Y_2}^2 - 2 I_{2} m_{Y_2}^4 + 4 I_{22} m_X^2 m_{Y_2}^4 \nonumber \\&- 20 I_{22} m_{Y_1}^2 m_{Y_2}^4 - 2 I_{22} m_{Y_2}^6. \end{aligned}$$

### B.6: $S\rightarrow SS$

The real corrections for the decays $S\rightarrow SS$ are given by

B.66 $$\begin{aligned} \Gamma _{S\rightarrow SS+\gamma /g}=\frac{c_g^2}{(4\pi )^3m_X}C'_Scc^*\Omega . \end{aligned}$$

The coupling $c_g$ is the electromagnetic coupling *e* or the strong coupling $g_s$ for photon and gluon emission, respectively. *c* is the coupling of the tree-level vertex of $S\rightarrow SS$. Therein we identify $S=X$, $S=Y_1$ and $S=Y_2$ with the indices 0, 1 and 2, respectively. The individual contributions $\Omega _{ij}$ are given by:

B.67 $$\begin{aligned} \Omega _{00} =&-4 I_0 - 8 I_{00} m_X^2 ,\end{aligned}$$

B.68 $$\begin{aligned} \Omega _{01} =&-4 I_0 - 4 I_1 + I_{01} (-8 m_X^2 - 8 m_{Y_1}^2 + 8 m_{Y_2}^2) ,\end{aligned}$$

B.69 $$\begin{aligned} \Omega _{02} =&-4 I_0 - 4 I_{2} + I_{02} (-8 m_X^2 + 8 m_{Y_1}^2 - 8 m_{Y_2}^2) ,\end{aligned}$$

B.70 $$\begin{aligned} \Omega _{11} =&-4 I_1 - 8 I_{11} m_{Y_1}^2 ,\end{aligned}$$

B.71 $$\begin{aligned} \Omega _{12} =&4 I_1 + 4 I_{2} + I_{12} (-8 m_X^2 + 8 m_{Y_1}^2 + 8 m_{Y_2}^2) ,\end{aligned}$$

B.72 $$\begin{aligned} \Omega _{22} =&-4 I_{2} - 8 I_{22} m_{Y_2}^2. \end{aligned}$$

### B.7: $S\rightarrow SV$

The real corrections for the decays $S\rightarrow SV$ are given by

B.73 $$\begin{aligned} \Gamma _{S\rightarrow SV+\gamma /g}=\frac{c_g^2}{(4\pi )^3m_{Y_2}^2m_X}C'_Scc^*\Omega . \end{aligned}$$

The coupling $c_g$ is the electromagnetic coupling *e* or the strong coupling $g_s$ for photon and gluon emission, respectively. In contrast to previous decays, $S\rightarrow SV+\gamma /g$ also has a contribution from a four-point interaction, which accordingly does not contribute to the infrared divergent part of the real corrections, but yields a finite contribution. The sum over the gauge factors thus includes four indices $0,\ldots ,3.$ Since we assume a non-extended gauge sector in this work, the four-point vertex is only present for photon emission, so we can simplify the calculation of the group-theory factors to $C_{ij}=Q_iQ_j$ for $i,j\in \lbrace 0,1,2\rbrace$, where the charges of the incoming and outgoing scalars are $Q_0$ and $Q_1$ and the charge of the outgoing gauge boson is $Q_2$; the factors involving the four-point interaction are then

B.74 $$\begin{aligned} C_{03}&= Q_0(Q_0+Q_1),&C_{13} = Q_1(Q_0+Q_1),\end{aligned}$$

B.75 $$\begin{aligned} C_{23}&= Q_2(Q_0+Q_1),&C_{33} = (Q_0+Q_1)^2. \end{aligned}$$

For the emission of a gluon we can use $Q_2=0$ and $Q_0=Q_1=1$. However, we stress that the results below are true in general for any gauge groups; for extended gauge sectors we would just need to employ Eq. (B.17). The individual contributions $\Omega _{ij}$ are given by

B.76 $$\begin{aligned} \nonumber \Omega _{00} =&-4 I m_X^2 + I_0 \left( -8 m_X^4 + 8 m_X^2 m_{Y_1}^2 + 8 m_X^2 m_{Y_2}^2\right) \\&+ I_{00} \left( -4 m_X^6 + 8 m_X^4 m_{Y_1}^2 - 4 m_X^2 m_{Y_1}^4 + 8 m_X^4 m_{Y_2}^2 \right. \nonumber \\&+\left. 8 m_X^2 m_{Y_1}^2 m_{Y_2}^2 - 4 m_X^2 m_{Y_2}^4\right) ,\end{aligned}$$

B.77 $$\begin{aligned} \nonumber \Omega _{01} =\,&I_0^1 \left( 4 m_X^2 - 4 m_{Y_1}^2 + 4 m_{Y_2}^2\right) \\&+ \,I_1^0 \left( -4 m_X^2 + 4 m_{Y_1}^2 + 4 m_{Y_2}^2\right) \nonumber \\&+ I \left( 4 m_X^2 + 4 m_{Y_1}^2 + 4 m_{Y_2}^2\right) \nonumber \\ \nonumber&+ I_1 \left( -8 m_X^4 + 8 m_X^2 m_{Y_1}^2 + 16 m_X^2 m_{Y_2}^2 \right. \\&+\left. 8 m_{Y_1}^2 m_{Y_2}^2 - 8 m_{Y_2}^4\right) + I_0 \left( 8 m_X^2 m_{Y_1}^2 - 8 m_{Y_1}^4\right. \nonumber \\ \nonumber&+ \left. 8 m_X^2 m_{Y_2}^2 + 16 m_{Y_1}^2 m_{Y_2}^2 - 8 m_{Y_2}^4\right) \\&+ I_{01} \left( -4 m_X^6 + 4 m_X^4 m_{Y_1}^2 + 4 m_X^2 m_{Y_1}^4 - 4 m_{Y_1}^6\right. \nonumber \\&+ 12 m_X^4 m_{Y_2}^2 + 8 m_X^2 m_{Y_1}^2 m_{Y_2}^2 + 12 m_{Y_1}^4 m_{Y_2}^2 \nonumber \\&-\left. 12 m_X^2 m_{Y_2}^4 - 12 m_{Y_1}^2 m_{Y_2}^4 + 4 m_{Y_2}^6\right) ,\end{aligned}$$

B.78 $$\begin{aligned} \nonumber \Omega _{02} =&\, I_0^2 \left( -2 m_X^2 + 2 m_{Y_1}^2 - 2 m_{Y_2}^2\right) \\&+ I \left( 2 m_X^2 + 6 m_{Y_1}^2 + 2 m_{Y_2}^2\right) + I_0 \left( 2 m_X^4 + 4 m_X^2 m_{Y_1}^2\right. \nonumber \\&-\left. 6 m_{Y_1}^4 + 4 m_X^2 m_{Y_2}^2 + 12 m_{Y_1}^2 m_{Y_2}^2 - 6 m_{Y_2}^4\right) \nonumber \\&+ I_{2} \left( -4 m_X^4 + 8 m_X^2 m_{Y_1}^2 - 4 m_{Y_1}^4\right. \nonumber \\ \nonumber&\left. + 4 m_{Y_2}^4\right) + I_{02} \left( -4 m_X^6 + 12 m_X^4 m_{Y_1}^2 - 12 m_X^2 m_{Y_1}^4 \right. \\&+ 4 m_{Y_1}^6 + 4 m_X^4 m_{Y_2}^2 + 8 m_X^2 m_{Y_1}^2 m_{Y_2}^2\nonumber \\&-\left. 12 m_{Y_1}^4 m_{Y_2}^2 + 4 m_X^2 m_{Y_2}^4 + 12 m_{Y_1}^2 m_{Y_2}^4 - 4 m_{Y_2}^6\right) ,\end{aligned}$$

B.79 $$\begin{aligned} \nonumber \Omega _{03} =&I \left( 2m_X^2 - 2m_{Y_1}^2 + 2m_{Y_2}^2\right) \\&+ I_0^2 \left( 2m_X^2 - 2m_{Y_1}^2 + 2m_{Y_2}^2\right) + I_0 \left( 2m_X^4 - 4 m_X^2 m_{Y_1}^2\right. \nonumber \\&+ \left. 2m_{Y_1}^4 - 4 m_X^2 m_{Y_2}^2 - 4 m_{Y_1}^2 m_{Y_2}^2 + 2 m_{Y_2}^4\right) ,\end{aligned}$$

B.80 $$\begin{aligned} \nonumber \Omega _{11} =&\, -4 I m_{Y_1}^2 + I_1 \left( 8 m_X^2 m_{Y_1}^2 - 8 m_{Y_1}^4 \right. \\&+\left. 8 m_{Y_1}^2 m_{Y_2}^2\right) + I_{11} \left( -4 m_X^4 m_{Y_1}^2 + 8 m_X^2 m_{Y_1}^4\right. \nonumber \\&- 4 m_{Y_1}^6 + 8 m_X^2 m_{Y_1}^2 m_{Y_2}^2 \nonumber \\&+\left. 8 m_{Y_1}^4 m_{Y_2}^2 - 4 m_{Y_1}^2 m_{Y_2}^4\right) ,\end{aligned}$$

B.81 $$\begin{aligned} \nonumber \Omega _{12} =&I \left( -6 m_X^2 - 2 m_{Y_1}^2 - 2 m_{Y_2}^2\right) + I_1^2 \left( -2 m_X^2 + 2 m_{Y_1}^2 \right. \\&+\left. 2 m_{Y_2}^2\right) + I_{2} \left( 4 m_X^4 - 8 m_X^2 m_{Y_1}^2\right. \nonumber \\ \nonumber&+\left. 4 m_{Y_1}^4 - 4 m_{Y_2}^4\right) + I_1 \left( 6 m_X^4 - 4 m_X^2 m_{Y_1}^2 - 2 m_{Y_1}^4 \right. \\&-\left. 12 m_X^2 m_{Y_2}^2 - 4 m_{Y_1}^2 m_{Y_2}^2 + 6 m_{Y_2}^4\right) \nonumber \\ \nonumber&+ I_{12} \left( -4 m_X^6 + 12 m_X^4 m_{Y_1}^2 - 12 m_X^2 m_{Y_1}^4 \right. \\&+ 4 m_{Y_1}^6 + 12 m_X^4 m_{Y_2}^2 - 8 m_X^2 m_{Y_1}^2 m_{Y_2}^2\nonumber \\&- 4 m_{Y_1}^4 m_{Y_2}^2 - 12 m_X^2 m_{Y_2}^4 \nonumber \\&-\left. 4 m_{Y_1}^2 m_{Y_2}^4 + 4 m_{Y_2}^6\right) ,\end{aligned}$$

B.82 $$\begin{aligned} \nonumber \Omega _{13} =&I \left( -2 m_X^2 + 2 m_{Y_1}^2 + 2 m_{Y_2}^2\right) + I_1^2 \left( -2 m_X^2 + 2 m_{Y_1}^2 \right. \\&+\left. 2 m_{Y_2}^2\right) + I_1 \left( 2 m_X^4 - 4 m_X^2 m_{Y_1}^2\right. \nonumber \\&+ \left. 2 m_{Y_1}^4 - 4 m_X^2 m_{Y_2}^2 - 4 m_{Y_1}^2 m_{Y_2}^2 + 2 m_{Y_2}^4\right) ,\end{aligned}$$

B.83 $$\begin{aligned} \nonumber \Omega _{22} =&I \left( -2 m_X^2 - 2 m_{Y_1}^2 - m_{Y_2}^2\right) + 8 I_2^1 m_{Y_2}^2 \\&+ 8 I_{22}^{11} m_{Y_2}^2 + I_{2} \left( 8 m_X^2 m_{Y_2}^2 + 8 m_{Y_1}^2 m_{Y_2}^2 - 8 m_{Y_2}^4\right) \nonumber \\&+ I_{22} \left( -4 m_X^4 m_{Y_2}^2 + 8 m_X^2 m_{Y_1}^2 m_{Y_2}^2 - 4 m_{Y_1}^4 m_{Y_2}^2 \right. \nonumber \\&\left. + 8 m_X^2 m_{Y_2}^4 + 8 m_{Y_1}^2 m_{Y_2}^4 - 4 m_{Y_2}^6\right) ,\end{aligned}$$

B.84 $$\begin{aligned} \Omega _{23} =&I \left( 2 m_X^2 - 2 m_{Y_1}^2 - 4 m_{Y_2}^2\right) - 8 I_2^1 m_{Y_2}^2 ,\end{aligned}$$

B.85 $$\begin{aligned} \Omega _{33} =&I m_{Y_2}^2. \end{aligned}$$

## Appendix C: Infrared divergent parts of Passarino–Veltman integrals

Subsequently we present the infrared divergent parts of the Passarino–Veltman integrals relevant for our purposes regularised through a photon or gluon mass $m_\Lambda$:

C.1 $$\begin{aligned}&\dot{B}_0(p^2,m_\Lambda ^2,p^2)=\dot{B}_0(p^2,p^2,m_\Lambda ^2)\nonumber \\&\quad =-\dot{B}_1(p^2,p^2,m_\Lambda ^2) = -\frac{1}{2p^2}\log \left( \frac{m_\Lambda ^2}{p^2}\right) ,\end{aligned}$$

C.2 $$\begin{aligned}&C_0\left( p_0^2,p_1^2,p_2^2,m_\Lambda ^2,p_0^2,p_2^2\right) = \frac{1}{\lambda (p_0^2,p_1^2,p_2^2)} \nonumber \\&\quad \times \log \left( \frac{p_0^2-p_1^2+p_2^2+\lambda (p_0^2,p_1^2,p_2^2)}{2p_0p_2}\right) \log \left( \frac{m_\Lambda ^2}{p_0p_2}\right) ,\end{aligned}$$

C.3 $$\begin{aligned}&C_0\left( p_0^2,p_1^2,p_2^2,p_0^2,m_\Lambda ^2,p_1^2\right) = \frac{1}{\lambda (p_0^2,p_1^2,p_2^2)}\nonumber \\&\qquad \times \log \left( \frac{p_0^2+p_1^2-p_2^2+\lambda (p_0^2,p_1^2,p_2^2)}{2p_0p_1}\right) \log \left( \frac{m_\Lambda ^2}{p_0p_1}\right) ,\end{aligned}$$

C.4 $$\begin{aligned}&C_0\left( p_0^2,p_1^2,p_2^2,p_2^2,p_1^2,m_\Lambda ^2\right) = \frac{1}{\lambda (p_0^2,p_1^2,p_2^2)}\nonumber \\&\quad \times \log \left( \frac{-p_0^2+p_1^2+p_2^2+\lambda (p_0^2,p_1^2,p_2^2)}{2p_1p_2}\right) \log \left( \frac{m_\Lambda ^2}{p_1p_2}\right) ,\end{aligned}$$

C.5 $$\begin{aligned}&C_1\left( p_0^2,p_1^2,p_2^2,p_0^2,m_\Lambda ^2,p_1^2\right) = -C_0\left( p_0,p_1,p_2,p_0,m_\Lambda ,p_1\right) ,\end{aligned}$$

C.6 $$\begin{aligned}&C_2\left( p_0^2,p_1^2,p_2^2,p_2^2,p_1^2,m_\Lambda ^2\right) = -C_0\left( p_0,p_1,p_2,p_2,p_1,m_\Lambda \right) ,\end{aligned}$$

C.7 $$\begin{aligned}&C_{11}\left( p_0^2,p_1^2,p_2^2,p_0^2,m_\Lambda ^2,p_1^2\right) = C_0(p_0,p_1,p_2,p_0,m_\Lambda ,p_1) ,\end{aligned}$$

C.8 $$\begin{aligned}&C_{22}\left( p_0^2,p_1^2,p_2^2,p_2^2,p_1^2,m_\Lambda ^2\right) = C_0(p_0,p_1,p_2,p_2,p_1,m_\Lambda ). \end{aligned}$$

Therein we use the Källén function given in Eq. (2.2). The result for $C_0$ is consistent with the infrared divergent part of Eq. (B.5) of Ref. [126]. For the cases $p_1^2,p_2^2\ll p_0^2$ and $p_2^2\ll p_1^2,p_0^2$, which is of particular relevance for scalar decays into light fermions, we also implemented formulae

C.9 $$\begin{aligned}&C_0(p_0^2,p_1^2,p_2^2,m_\Lambda ^2,p_0^2,p_2^2) \nonumber \\&\quad = \frac{1}{p_2^2}\log \left( \frac{m_\Lambda ^2}{-p_2^2} \right) \log \left( \frac{p_0 p_1}{-p_2^2} \right) ,\end{aligned}$$

C.10 $$\begin{aligned}&C_0(p_0^2,p_1^2,p_2^2,m_\Lambda ^2,p_0^2,p_2^2)\nonumber \\&\quad = \frac{1}{p_2^2 - p_1^2} \log \left( \frac{p_0(p_1^2-p_2^2)}{m_\Lambda ^2 p_1} \right) \log \left( \frac{p_1^3-p_2^2}{p_0 p_1} \right) . \end{aligned}$$

These are equivalent to Eqs. (B.8) and (B.9) of Ref. [126].

## Appendix D: Goldstone boson vertices

For decays into scalars and fermions, the cancellation of infrared divergences is straightforward: they correspond to summing the real emission of a massless gauge boson with the virtual process of the same massless gauge boson in the loop. Because the current of the unbroken gauge symmetries are necessarily flavour diagonal, the only vertices involved are the original two-body decay vertex and the gauge couplings of the external states. Therefore, when we want to use loop-corrected external masses to cancel the infrared divergences for this case it is straightforward to either put all masses of internal and external states to the loop-corrected values, or (as we do here) to subtract off the infrared divergent parts separately from the Bremsstrahlung and virtual corrections before multiplying by a kinematic factor employing loop-corrected masses.

However, for decays with a massive gauge boson in the final state, the cancellation between the real and virtual infrared divergences is a little subtle. The reason is that a would-be Goldstone boson can propagate as an internal state. While we perform the Bremsstrahlung calculation in the unitary gauge (so there are no Goldstone bosons), for the virtual corrections for practical purposes the default choice is Feynman–’t Hooft gauge, and we must therefore sum the diagrams with an internal massive gauge boson with those having a massive Goldstone boson. We show the relevant virtual corrections for the processes $F\rightarrow FV$, $S\rightarrow SV$ and $S\rightarrow VV$ (for decays to massive vectors) in Figs. 21, 22 and 23, respectively. Denoting the heavy gauge boson as “*V*”, the massless one as “$\gamma$,” and the Goldstone boson as “*G*,” we see that we have the gauge coupling of the unbroken group appearing in $FF\gamma , SS\gamma$ and $VV\gamma$ vertices, but we must maintain a relationship between the *FFV* and *FFG* vertices, between the *SSV* and *SSG* vertices, the $VV\gamma$ and $VG\gamma$ vertices, in order for these cancellations to occur. For the $S\rightarrow VV$ process, we require a relationship between the three sets of vertices *SVV*, *SGV* and *SGG*.

The required relations follow from the Slavnov–Taylor identities, or alternatively we could examine the infrared divergent part of the loop amplitudes. However, here we will more simply derive the conditions imposed by symmetry when inspecting the lagrangian at tree level. We first identify the would-be Goldstone boson by writing the symmetry transformations of real scalars $S_i^0$ (before spontaneous symmetry breaking, and not necessarily in a mass-diagonal basis) as

D.1 $$\begin{aligned} \delta S_i^0 \equiv \epsilon ^G \alpha _i^G = \epsilon ^G a_{ij}^G S_j^0, \end{aligned}$$

where $\epsilon ^G$ is the gauge transformation parameter for each broken direction *G* and $a_{ij}^G$ are numbers for a linearly realised gauge symmetry (as always assumed in SARAH). Then

D.2 $$\begin{aligned} a_{ij}^G = t_{ij}^G \end{aligned}$$

where $t_{ij}^G$ are the generators of the gauge symmetry that will be broken by appropriate vacuum expectation values; since we are working with real scalars, the $t_{ij}^G$ are antisymmetric and real (to translate to complex fields we require roughly $t_{ij}^G \rightarrow i T^G_{ij}$). Then we find that the Goldstones *G* are defined by

D.3 $$\begin{aligned} G = N_G \alpha _j^G S_j^0, \qquad S_i^0 = N_G \alpha _i^G G + \cdots . \end{aligned}$$

The vectors should be chosen to be orthogonal with normalisation 1, so $N_G = 1/\sqrt{\sum _i (\alpha _i^G)^2}$.

Looking at the vector-mass term we find for the scalars $S_i^0$ having vacuum expectation values $v_i$

D.4 $$\begin{aligned}&-\frac{1}{2} D_\mu S_i^0 D^\mu S_i^0 \supset g \partial _\mu S_i^0 V^{a\, \mu } t^a_{ij} v_j\nonumber \\&\qquad - \frac{g^2}{2} t_{ij}^a v_j t_{ik}^b v_k V_\mu ^a V^{b\, \mu } - g^2 t_{ij}^a S_j^0 t_{ik}^b v_k V_\mu ^a V^{b\, \mu }\nonumber \\&\qquad +\frac{g}{2} t_{ij}^a V^{a\, \mu } (S_j^0 \partial _\mu S_i^0 - S_i^0 \partial _\mu S_j^0) \nonumber \\&\quad \supset g \alpha _i^G \partial _\mu S_i^0 V^{G\, \mu } - \frac{g^2}{2} \alpha _i^G \alpha _i^{G'} v_k V_\mu ^G V^{G'\, \mu } \nonumber \\&\qquad - g^2 \alpha _i^G t_{ij}^a S_j^0 V_\mu ^a V^{G\, \mu } \nonumber \\&\quad \supset - \frac{g^2}{2} \sum _G \frac{1}{N_G^2} V_\mu ^G V^{G\, \mu }, \end{aligned}$$

and we see that

D.5 $$\begin{aligned} N_G =&\frac{g}{m_V^G}, \end{aligned}$$

where $m_V^G$ is the mass of the vector, and we define the *SSV* coupling when we diagonalise the scalars to mass eigenstates through $S_i^0 \equiv R_{ik} S_k$ (where $R_{Gj} = N_G \alpha _j^G$):

D.6 $$\begin{aligned} \mathcal {L}\supset&\frac{1}{2} c_{ij}^a V^{a\, \mu } (S_j \partial _\mu S_i - S_i \partial _\mu S_j) \rightarrow c_{ij}^a = g R_{ik} R_{jl} t_{kl}^a. \end{aligned}$$

For the *SGV* coupling and *SVV* couplings, we read off

D.7 $$\begin{aligned}&\mathcal {L}\supset \frac{1}{2} c_{iG}^{G'} V^{G'\, \mu } (G \partial _\mu S_i - S_i \partial _\mu G) + c_i^{GG'} V_{\mu }^G V^{G'\mu } \nonumber \\&\quad \rightarrow c_{iG}^{G'} = -\frac{g^2}{m_V^G} R_{ki} \alpha _j^G t_{jk}^{G'}, \qquad c_i^{GG'} = - g^2 R_{ki} \alpha _j^G t^a_{jk} \nonumber \\&\quad \rightarrow c_{iG}^{G'} = \frac{1}{m_V^G} c_i^{GG'}. \end{aligned}$$

This is the relationship that we enforce between the on-shell vertices to ensure that infrared divergences are cancelled.

We can also read off the *GVV* coupling

D.8 $$\begin{aligned} - g^2 \alpha _i^G T_{ij}^a S_j^0 V_\mu ^a V^{G\, \mu } \supset&- g^2 N_G \alpha _i^{G'} t_{ij}^a \alpha _j^G G V_\mu ^a V^{G'\, \mu }. \end{aligned}$$

For any unbroken gauge groups, the Goldstones must transform as

D.9 $$\begin{aligned} \mathcal {L}\supset&\frac{g}{2} N_G^2 \alpha _i^G t_{ij}^a \alpha _j^{G'} \gamma ^{a\, \mu } (G' \partial _\mu G - G \partial _\mu G') \nonumber \\ =&\frac{g}{2} t_{GG'}^a \gamma ^{a\, \mu } (G' \partial _\mu G - G \partial _\mu G') \end{aligned}$$

and we therefore identify the $\alpha _i^{G'} t_{ij}^a \alpha _j^G$ factor in Eq. (D.8) with $T_{GG'}^a$ to obtain

D.10 $$\begin{aligned} \mathcal {L}\supset - g m_V^G t^a_{G'G} G' \gamma _\mu ^a V^{G\, \mu }. \end{aligned}$$

For the photon, this just becomes the familiar vertex

D.11 $$\begin{aligned} \mathcal {L}\supset - e m_W G^+ \gamma _\mu ^a W^{-\,\mu } + \text {h.c.}. \end{aligned}$$

Now the $VV\gamma$ coupling will be given just in terms of the gauge coupling of the unbroken gauge group, so the electromagnetic coupling *e* here; indeed from decomposing the kinetic terms of the gauge bosons we will find

D.12 $$\begin{aligned}&\mathcal {L}\supset c^{aGG'} \bigg (\partial ^\mu \gamma _\nu ^a V^{G}_\mu V^{G'\,\nu } - V_\mu ^G \partial _\nu \gamma ^{a\,\mu } V^{G'\,\nu } \nonumber \\&\quad - V_\mu ^G \gamma ^{a\,\mu } \partial _\nu V^{G'\,\nu } + \gamma ^{a}_\mu V^{G}_\nu \partial ^\mu V^{G'\,\nu } \bigg ). \end{aligned}$$

Of these, the first two terms vanish in the limit where the massless gauge boson is soft, while we identify the last term as the conventional gauge current and

D.13 $$\begin{aligned} c^{aGG'} =&-g t^{a}_{GG'}. \end{aligned}$$

This leads to the relation between the $\gamma GV$ and $\gamma VV$ vertices of simply a factor of $m_V^G$.

To find the relationship between the Goldstone coupling to scalars and the gauge boson coupling, we can use Eq. (2.32) of Ref. [127] to find the derivative of the (effective) potential *V* with respect to the scalars:

D.14 $$\begin{aligned} \frac{\partial ^3V}{\partial G \partial S_i^0 \partial S_j^0} =&-N \frac{\partial \alpha _k^G}{\partial \phi _i} \frac{\partial ^2 V}{\partial \phi _k \partial \phi _j} -N\frac{\partial \alpha _k^G}{\partial \phi _j} \frac{\partial ^2 V}{\partial \phi _k \partial \phi _i} \nonumber \\ =&- \frac{1}{m_V} \bigg [ gt_{ki}^G \mathcal {M}_{kj}^2 + \mathcal {M}_{ik}^2 gt_{kj}^G \bigg ]. \end{aligned}$$

When we diagonalise the masses this gives

D.15 $$\begin{aligned} c_{ijG} \equiv - \frac{\partial ^3V}{\partial G \partial S_i \partial S_j} = \frac{1}{m_V^G} \left( m_i^2 - m_j^2\right) c_{ij}^G. \end{aligned}$$

Again we enforce this relationship between the *GSS* coupling and the *SSV* coupling when we use loop-corrected masses in order to cancel infrared divergences.

The *GGS* coupling is a special case of the above, with the understanding that we must use zero for the Goldstone boson mass to obtain

D.16 $$\begin{aligned} c_{iGG'} = \frac{m_i^2}{m_V^G m_V^{G'}} c_i^{GG'}. \end{aligned}$$

We also find a similar relationship for fermions, which can in that case also be derived from the fact that the masses are Yukawa couplings; writing in terms of Weyl fermions we have

D.17 $$\begin{aligned} \mathcal {L}\supset&-\frac{1}{2} Y_{i IJ} S_i^0 \psi _I^0 \psi _J^0 \nonumber \\ =&-\frac{1}{2}\mathcal {M}_{IJ} \psi _I^0 \psi _J^0 - \frac{g}{2m_V} Y_{iIJ} t^G_{ij} v_j G\psi _I^0 \psi _J^0 + \cdots . \end{aligned}$$

The gauge invariance of the Yukawa coupling gives

D.18 $$\begin{aligned} Y_{i' IJ} t^a_{i'i} (i) + Y_{iI'J} t^a_{I'I} (I) + Y_{iIJ'} t^a_{J'J} (J) =&0 \end{aligned}$$

and so

D.19 $$\begin{aligned}&- \frac{g}{2m_V} Y_{iIJ} t^G_{ij} (i) v_j \end{aligned}$$

D.20 $$\begin{aligned}&\quad = \frac{g}{2m_V} Y_{iI'J} v_i t^G_{I'I} (I) + \frac{g}{2m_V} Y_{iIJ'} v_i t^G_{J'J}(J) \nonumber \nonumber \\&\quad = \frac{g}{2m_V} \bigg ( \mathcal {M}_{I'J} t^G_{I'I} + \mathcal {M}_{IJ'} t^G_{J'J}(J) \bigg ). \end{aligned}$$

Now we write down the gauge coupling

D.21 $$\begin{aligned} \mathcal {L}\supset&g\overline{\psi }_I \overline{\sigma }^\mu \psi _J t^a_{IJ} V^a \end{aligned}$$

and we diagonalise into $\psi _I^0 = R_{IJ} \psi _J$ so we obtain

D.22 $$\begin{aligned} \mathcal {L}\supset&\, c_{IJ}^G \overline{\psi }_I \overline{\sigma }^\mu \psi _J V^{G\, \mu } + \frac{1}{2} c_{IJ G} \psi _I \psi _J G ,\nonumber \\ c_{IJG} =\,&\frac{m_I - m_J}{m_V^G} c_{IJ}^G. \end{aligned}$$

If we now split the fermions into left- and right-handed states with separate rotation matrices *L* and *R*, then the gauge couplings are given in Dirac notation by

D.23 $$\begin{aligned}&\mathcal {L}\supset g \overline{F}_I \gamma ^\mu P_L F_J (L^\dagger t^a L)_{IJ} V^a \nonumber \\&\quad + g \overline{F}_M \gamma ^\mu P_R F_N (R^T t^a R^*)_{MN} V^a, \end{aligned}$$

while the Goldstone couplings are

D.24 $$\begin{aligned} \mathcal {L}\supset&\frac{g}{m_V} \bigg ( \mathcal {M}_{I'J} t^G_{I'I} + \mathcal {M}_{IJ'} t^G_{J'J}(J) \bigg ) G \psi _I^L \psi _J^R + \text {h.c.} \nonumber \\ \rightarrow&\frac{g}{m_V} \bigg ( - m_J (R^T t^G R)_{IJ} + (L^T t^G L)_{IJ} m_I \bigg ) G \overline{F}_I P_L F_J \nonumber \\&+ \frac{g}{m_V} \bigg ( m_I^* (R^\dagger t^G R^*)_{IJ} \!-\! (L^\dagger t^G L^*)_{IJ} m_J^* \bigg ) G \overline{F}_I P_R F_J\, . \end{aligned}$$

If we work in a basis where the fermion masses are complex and the rotation matrices *R*, *L* are real, then we find the relationship between the Goldstone boson couplings and the gauge couplings

D.25 $$\begin{aligned} c_{IJG}^{L} =&\frac{1}{m_V^G} \left[ m_I c_{IJ}^{G, L} - m_J c_{IJ}^{G, R} \right] ,\nonumber \\ c_{IJG}^{R} =&-\frac{1}{m_V^G} \left[ m_J^* c_{IJ}^{G, L} - m_I^* c_{IJ}^{G, R} \right] . \end{aligned}$$

## Footnotes

1 In the calculation of one-loop decays we introduced a minimal allowed mass for fermions and scalars of $10^{-15}$ GeV. Smaller masses are set to zero to stabilise numerics, see Sect. 5.3.1 for a discussion in the context of *R*-parity violation.

2
FormCalc works with constrained differential renormalisation [95], which equals dimensional reduction at the one-loop level [94].

3 Also on-shell schemes can lead to finite shifts at higher orders, see e.g. Refs. [65, 105, 106] for examples in the neutralino and chargino sector.

4 It differs by the prescription for how the self-energies $\Pi$ are renormalised.

5 In a private discussion with one author of SFOLD the origin of the problem could not be identified. It might be a numerical problem with the high-rank loop integrals which appear when performing the calculation in $R_\xi$ gauge.

6 Even if SARAH will use the new routines to obtain also loop-induced decay width for Higgs states, one should still use the old results for the diphoton and digluon rates. The latter also include the full model dependence at LO but in addition also higher-order QCD corrections, see Ref. [122].
